# How do changes in flow magnitude due to hydropower operations affect fish abundance and biomass in temperate regions? A systematic review

**DOI:** 10.1186/s13750-021-00254-8

**Published:** 2022-02-04

**Authors:** Meagan Harper, Trina Rytwinski, Jessica J. Taylor, Joseph R. Bennett, Karen E. Smokorowski, Julian D. Olden, Keith D. Clarke, Tom Pratt, Neil Fisher, Alf Leake, Steven J. Cooke

**Affiliations:** 1 Fish Ecology and Conservation Physiology Laboratory, Department of Biology, Carleton University, Ottawa, ON Canada; 2 Canadian Centre for Evidence-Based Conservation, Institute of Environmental and Interdisciplinary Science, Carleton University, Ottawa, ON Canada; 3 Department of Biology and Institute of Environmental and Interdisciplinary Science, Carleton University, Ottawa, ON Canada; 4 Great Lakes Laboratory for Fisheries and Aquatic Sciences, Fisheries and Oceans Canada, Sault Ste. Marie, ON Canada; 5 School of Aquatic and Fishery Sciences, University of Washington, Seattle, WA USA; 6 Fisheries and Oceans Canada, Northwest Atlantic Fisheries Centre, St. John’s, NF Canada; 7 Freshwater Institute, Fisheries and Oceans Canada, 501 University Crescent, Winnipeg, MB Canada; 8 BC Hydro Environment, Burnaby, BC Canada

**Keywords:** Anthropogenic impacts, Dam, Discharge, Evidence synthesis, Fish density, Flow modification, Hydroelectric power

## Abstract

**Background:**

Altering the natural flow regime, an essential component of healthy fluvial systems, through hydropower operations has the potential to negatively impact freshwater fish populations. Establishing improved management of flow regimes requires better understanding of how fish respond to altered flow components, such as flow magnitude. Based on the results of a recent systematic map on the impacts of flow regime changes on direct outcomes of freshwater or estuarine fish productivity, evidence clusters on fish abundance and biomass responses were identified for full systematic review. The primary goal of this systematic review is to address one of those evidence clusters, with the following research question: how do changes in flow magnitude due to hydropower operations affect fish abundance and biomass?

**Methods:**

This review follows the guidelines of the Collaboration for Environmental Evidence. It examined commercially published and grey literature originally identified during the systematic map process and a systematic search update. All articles were screened using an a priori eligibility criteria at two stages (title and abstract, and full-text) and consistency checks were performed at all stages. All eligible articles were assessed for study validity and specifically designed data extraction and study validity tools were used. A narrative synthesis included all available evidence and meta-analysis using the standardized mean difference (Hedges’ *g*) was conducted where appropriate.

**Review findings:**

A total of 133 studies from 103 articles were included in this systematic review for data extraction and critical appraisal. Most studies were from North America (60%) and were conducted at 146 different hydropower dams/facilities. Meta-analysis included 268 datasets from 58 studies, separated into three analyses based on replication type [temporal (within or between year replication) or spatial]. Fish abundance (226 datasets) and biomass (30 datasets) had variable responses to changes in flow magnitude with estimated overall mean effect sizes ranging from positive to negative and varying by study design and taxa. In studies with temporal replication, we found a detectable effect of alterations to the direction of flow magnitude, the presence of other flow components, sampling methods, season, and fish life stage. However, we found no detectable effect of these moderators for studies with spatial replication. Taxonomic analyses indicated variable responses to changes in flow magnitude and a bias towards salmonid species.

**Conclusions:**

This synthesis did not find consistent patterns in fish abundance or biomass responses to alterations or changes in flow magnitude. Fish responses to flow magnitude alterations or changes were highly variable and context dependent. Our synthesis suggests that biotic responses may not be generalizable across systems impacted by hydroelectric power production and operations, where specific features of the system may be highly influential. Site-specific and adaptive management may be necessary. To improve study validity and interpretability, studies with long-term continuous monitoring, and both temporal and spatial replication are needed. When this gold standard is unfeasible, studies should strive, at minimum, to maximize replication within both intervention and comparator groups for either temporal or spatial designs. To further address knowledge gaps, studies are needed that focus on non-salmonids, multiple seasons, and systems outside of North America.

**Supplementary Information:**

The online version contains supplementary material available at 10.1186/s13750-021-00254-8.

## Background

Humans are an agent of significant change on Earth, altering ecosystems in diverse, often detrimental ways [1]. Alterations of ecosystems span terrestrial and aquatic systems but are exceptionally apparent in fluvial systems (i.e., rivers and streams) where dams, constructed to provide flood control, water storage and/or to generate hydroelectricity, have dramatically altered ecosystem structure and function (e.g., Nilsson et al. [2]; Winemiller et al. [3]). Globally, there are currently over 8600 hydropower dams higher than 15 m and future hydropower projects, in development, are expected to double hydropower generation capacity over the next 50 years [4]. Consequently, a better understanding of the impacts and ecosystem alterations associated with these projects is increasingly essential for effective management of both hydropower production and riverine conservation.

Almost half of all rivers globally are altered by river regulation or fragmentation [5], and hydropower dams are a major contributor to these alterations. Hydroelectric power production (HPP) is recognized as a continuing threat to freshwater species [6], especially with the increasing construction of both large and small HPP facilities [4, 7]. With hydropower expected to continue to represent a large portion of many countries’ energy portfolios [8] and to be increasingly utilized with the global move towards greener energy sources [4, 9, 10], understanding how alterations of specific flow components impact fish responses is essential. The effective management of flow regimes to provide flow characteristics that support both fish productivity and energy production in systems affected by HPP, requires a better understanding of how fish respond to flow component alterations at hydroelectric dams, and may even require a re-evaluation of how modified river flows are designed (e.g., Soininen et al. [11]; Tonkin et al. [12]).

Understanding hydrological and hydrodynamic alterations caused by HPP and related operations requires careful examination of riverine flow regimes, including magnitude, duration, frequency, timing and rate of change. Natural flow regimes have shaped both the geology and biology of riverine fluvial systems through time [13–15] and aquatic biota have evolved and adapted to the specific dynamics of their environment [13–16]. One of the critical components of a flow regime is flow magnitude (also called discharge), the measure of the volume of water passing a fixed location per unit of time (e.g., m^3^/s) [13]. Alterations to flow magnitude associated with HPP result in either increases or decreases in flow magnitude and can disrupt natural processes resulting in a variety of environmental and species responses [14]. Understanding how these alterations impact fluvial systems is important for water resource and fisheries management.

**Fig. 1:**
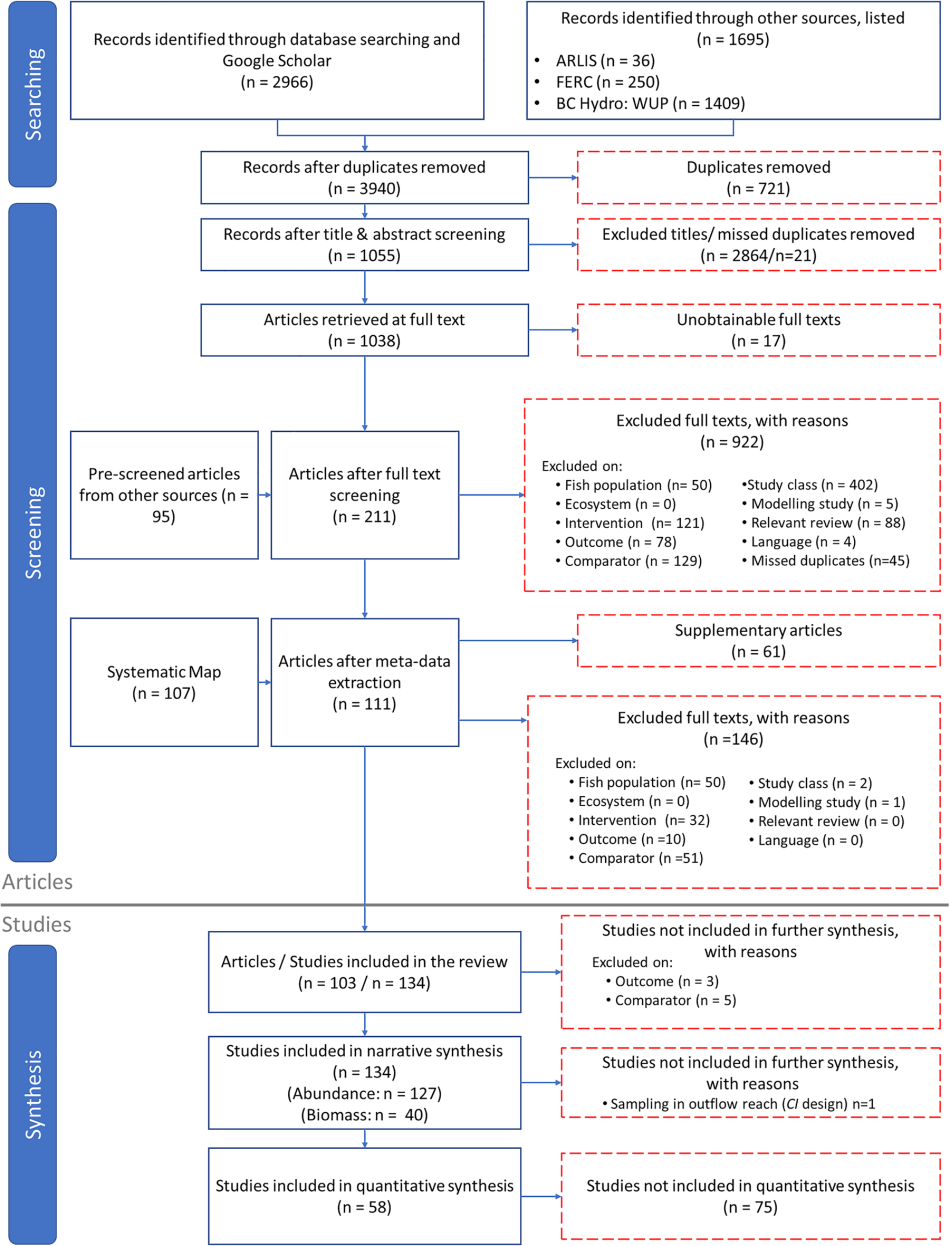
ROSES flow diagram [35] showing results of the literature search and study selection process showing the final number of studies included in the systematic review. Blue indicates articles/studies proceeded to next stage of review; red dashed lines indicate articles/studies were removed from consideration at that stage

The effects of HPP on fish living in or traveling through fluvial systems can include alterations to fish abundance (number of individuals, often quantified in terms of fish per area or catch) and biomass (total mass of individuals by area or volume) which may decrease or increase in response to these changes in flow (see Fig. 1 in our a priori systematic review protocol [17] for a simple conceptual model). Studies have shown that community abundance and biomass can differ between areas that are regulated by HPP facilities and those that are not (e.g., Kinsolving and Bain [18]; Guénard et al. [19]). Fish abundance has also been found to decrease after decreases in flow magnitude [20, 21], but conversely, may increase after the establishment of positive changes such as increases in minimum-flow releases (e.g., fluvial specialists increased in density compared to before-minimum conditions; Travnichek et al. [22]). Additionally, systems with flow magnitude that more closely mimics natural flow may have greater abundance than those with more altered flow magnitudes (e.g., systems with a legislated minimum flow magnitude compared to those without; Göthe et al. [23]). These studies indicate that fish responses may be dependent on the type of HPP facility, the type of designed flow regime (Acreman et al. [24]), and the magnitude of deviation or shift from the natural flow regime.

Available evidence syntheses on the impacts of HPP on fish often focus on the effects of passage on behaviour, injury and/or mortality of fish due to HPP facilities [25–27], or on the alteration in abundance and diversity of fish populations resulting from specific types of hydropower operation (i.e., hydropeaking; Melcher et al. [28]) or design (i.e., impoundments; Turgeon et al. [29]). While reviews on ecological responses to altered flows have been done in the past [21, 30, 31] including a recent summary of the various aspects of ecohydrology river alterations on fish [32], there remains a need to update our understanding of specific fish-flow interactions using robust systematic review techniques. Additionally, there is a need to reduce the uncertainty surrounding how fish respond to alterations in specific flow components such as flow magnitude [33].

A systematic review of how flow components such as magnitude, altered by HPP, affects fish abundance and biomass could help support effective flow management decision-making. Here, we use a systematic review approach, based on our a priori protocol [17] including meta-analysis, to evaluate the existing literature base to assess the consequences of alterations to flow magnitude by HPP on fish abundance and biomass. We also identify to what extent factors such as dam size, operational regime, direction of flow magnitude alteration, and fish life history characteristics influence the response of fish abundance and biomass to alterations in flow magnitude.

## Identification of review topic and stakeholder engagement

At the request of Canadian stakeholders [i.e., Fisheries and Oceans Canada (DFO)], a systematic map was recently conducted (Rytwinski et al. [33]) to provide a summary of the existing literature base on the impacts of flow regime changes on direct outcomes of freshwater or estuarine fish productivity (i.e., the map described the quantity and key characteristics of the available evidence, identified evidence clusters and knowledge gaps, but did not synthesize results). A total of 1368 relevant studies describing a variety of flow regime alterations and fish productivity responses were identified. The map focused on global temperate regions to ensure relevance to Canadian stakeholders and followed the Collaboration for Environmental Evidence (CEE) guidelines for systematic mapping [34].

From the systematic map, 11 potential topic clusters were identified as areas that had sufficient coverage to allow for systematic reviewing. The subtopic “the effect of alterations to flow magnitude due to hydroelectric power production on fish abundance” was identified as a candidate for full systematic reviewing based on the presence of sufficient evidence and interest from Canadian stakeholders.

An Advisory Team made up of stakeholders and experts including academic scientists from Canada and USA (four members), staff from DFO, specifically the Fish and Fish Habitat Protection Program (FFHPP) (one member), and the Science Branch (three members), as well as staff from the hydropower industry (one member), was established and consulted during this review process. The Advisory Team was consulted in the development of the inclusion criteria for article screening and data extraction strategy and participated throughout the course of this systematic review.

## Objective of the review

The objective of this systematic review was to clarify, from existing literature, how fish abundance and biomass are impacted by alterations or changes of flow magnitude due to hydroelectric power production (or related operations) to better inform decisions in water resource and fisheries management downstream of these facilities.

### Primary question

How do changes in flow magnitude due to hydroelectric power production affect fish abundance and biomass in temperate regions?

### Components of the primary question

The primary study question can be broken into the following PICO (Population, Intervention, Comparator, Outcome) components:

*Subject (population)—*freshwater and estuarine fish in temperate regions.

*Intervention/exposure—*changes to (or manipulation of) flow magnitude due to hydroelectric power production.

*Comparator—*no intervention or alternate levels of intervention.

*Outcomes—*measures of changes in abundance (e.g., abundance, density, catch per unit effort), and biomass (e.g., biomass, yield).

## Methods

This review followed the CEE guidelines and standards for systematic reviews [34] and conformed to ROSES reporting standards [35] (see Additional file 1). The methods of this review follow those published in an a priori systematic review protocol (Harper et al*.* [17]). We summarize the methods here and describe any deviations from the protocol made during the conduct of the review, below.

### Searching for articles

#### Selection of studies identified in the systematic map

Much of the evidence for this systematic review was identified in the recent Rytwinski et al. [33] systematic map on fish productivity and flow alteration. The systematic mapping process searched for commercially published and grey literature using six bibliographic databases (performed in July 2017), one search engine (July 2017) and 29 specialist websites (Feb. 2017). No date restrictions were applied for searches (see the Additional files from Rytwinski et al. [33] for full search details of the systematic map). Relevant reviews (297) and all accepted articles were also hand-searched for relevant titles not found using the search strategy. Calls for evidence to target grey literature were issued through relevant mailing lists, social media and the networks and colleagues of Advisory Team members. A total of 1368 relevant studies (published between 1940 and 2017) were identified by this map, with 74 considering flow magnitude alterations and fish abundance and 24 considering fish biomass metrics. All potentially relevant studies identified by the systematic map were included for this review at the data extraction stage and then screened on the specific eligibility criteria of this review.

#### Search update

##### Search terms and language

Search terms used in the systematic map prior to this systematic review, that identified studies considering the impacts of alteration to any component of flow on fish productivity, can be found in Additional file 2: Table S10.

An updated search was conducted on a subset of the search terms used in the systematic map (Table 1). These terms were used to query bibliographic databases (see section “Publication databases”) and the search engine, Google Scholar (see section “Search engines”). The updated search covered literature published from 2017 through 2019. Search terms were limited to English language due to project resource restrictions; however, no language, geographic, or document type restrictions were applied during the search. The search string was modified depending on the search functionality of different databases or the search engine (see Additional file 2). Full details on search settings and subscriptions used to access articles can be found in Additional file 2.

**Table 1:** Search string used to update searches from 2017 through 2019

Component	Search string
Population terms	((Fish*) AND (“Fresh water” OR Freshwater OR Stream$ OR Water$ OR River$ OR Fluvial OR Estuar* OR Reservoir$ OR Impoundment$ OR "Hydro electric*" OR Hydroelectric* OR "Hydro dam*" OR Hydrodam* OR "Hydro power" OR Hydropower OR "Hydro" OR Dam$))
	AND
Intervention/exposure terms	(Flow* OR Discharg*)
	AND
Outcome terms	(Productivity OR Biomass OR Abundance$ OR Densit* OR Yield$ OR “Ecological response” OR “Ecosystem response” OR “Biotic response”)
	NOT
Exclusionary terms	(Mining OR "Mine site" OR Aquaculture OR "Wastewater treatment" OR Carbon)

##### Publication databases

To ensure sufficient coverage and specificity during searching, the principal search system ISI Web of Science Core Collection [36] was used and an additional five bibliographic databases were accessed. All databases (listed below) were originally searched in the map and search updates occurred in November–December 2019 using Carleton University’s institutional subscriptions (see Additional file 2):

1. ISI Web of Science Core Collection
2. ProQuest Dissertation and Theses Global
3. Scopus
4. Federal Science Library (Canada)
5. Science.gov
6. AGRICOLA (Agricultural Research Database)

##### Search engines

To supplement our principal searches and identify potentially useful documents not already found by database searches, the same search engine originally used in the systematic map, Google Scholar, was searched January 2020 (first 500 hits sorted by relevance). Potentially relevant documents were recorded and included to be screened for appropriate fit with the review question. Customized search strings were used due to limited search capability of the search engine (see Additional file 2).

##### Specialist websites

Twenty-nine specialist organization websites were searched in the systematic map using abbreviated search terms (see Rytwinski et al. [33]). A search update was not conducted for these websites because it is often not possible to specifically filter by date using the built-in search functions of these websites.

##### Supplemental searches

Reference sections of accepted articles and 110 relevant reviews (2 relevant reviews were removed as duplicates) were hand-searched to evaluate relevant titles, published from 2017 forward, not identified using the search update strategy (see Additional file 3 for a list of relevant reviews). Stakeholders were consulted for advice for new sources of information. We also issued a call for evidence to experts and practitioners in the field to target grey literature through relevant mailing lists (see Additional file 2) and through social media (e.g., Twitter) in December 2019. The call for evidence was also distributed by the Advisory Team to relevant networks and colleagues. If experts and practitioners suggested websites or databases not already captured during the systematic mapping exercise or search update, these sites were either hand-searched (and articles included for screening at full-text) or, where possible, searched using built-in functions and modified keywords (see Additional file 2). These included:

1. ARLIS—Alaska Resources Library and Information Services
2. FERC Online eLibrary—US Federal Energy Regulatory Commission eLibrary

In cases where articles were found using built-in search functions, articles were included for eligibility screening at the title and abstract stage (see Additional file 2). To increase the chance of capturing previously missed unpublished information from expert and practitioner recommendations, no date restrictions were applied. Additionally, in one case, experts and practitioners suggested a specific set of documents from a website already accessed during the systematic map (BC Hydro, Water Use Plans). Water Use Plan projects were screened at title and abstract (e.g., we removed projects on ineligible topics such as archeology). Articles within applicable projects were accessed and checked against the results of the systematic map for duplicates. To ensure all relevant articles were captured, articles from applicable projects not previously identified were included for eligibility screening at title and abstract, and full-text screening.

##### Estimating comprehensiveness of the search

For this review, we did not repeat tests for comprehensiveness originally performed in the systematic map (i.e., the search results were checked against a benchmark list of 13 relevant papers provided by the advisory team to ensure all articles were captured using the search strategy). Because the review followed the same basic search strategy and used a search string similar to the systematic map [33], further comprehensiveness checks were not necessary. Most articles included as relevant in the systematic map (using a broader eligibility criteria than this review) were identified through databases and search engines (88%), reference sections of reviews and included articles, or through calls for evidence (9%), with only 3% being identified through website searches. We therefore considered it sufficient to base the search update on the same databases and search engine used in the systematic map, complemented with the supplemental searches described above. More specifically, we increased the likelihood of capturing relevant literature not identified from our search strategy by screening bibliographies of: (1) 110 individual relevant reviews identified at title and abstract or full-text; (2) accepted articles. We searched these reference lists until the reviewer (MH) deemed that the number of relevant returns had significantly decreased.

### Search record databases

Once all searches were complete and references were compiled, individual databases were exported to EPPI-reviewer (eppi.io.ac.uk/eppireviewer4) as one database. Prior to screening, duplicates were identified using the duplicate checking function in EPPI Reviewer and then manually removed by one reviewer (MH). All references, regardless of their perceived relevance to the systematic review, were included in the database. Results of supplemental searches were compiled in MS Excel and screened separately. Duplicate checks between the EPPI-reviewer database and supplementary searches were conducted by one reviewer (MH). When duplicates were missed during any previous stage, they were removed at later stages in the review.

### Article screening and study eligibility criteria

#### Screening process

Articles found by database searches and the search engine (including those suggested during calls for literature) were screened at two stages: (i) title and abstract, and (ii) full-text. Other articles found through supplemental searches were screened at full-text. No articles found through supplemental searches were included in consistency checks. Prior to screening all articles, a consistency check was done at the title and abstract stage where two reviewers (JJT and MH) independently screened 181/1810 articles [10% of the articles included in the EPPI Reviewer (which did not include evidence items found through supplemental searches or literature identified by the systematic map)]. The reviewers agreed on 93.34% of the articles (kappa = 0.59; moderate agreement). Any disagreements between reviewers were discussed and the inclusion criteria clarified, before moving forward. Following consistency checks, articles were screened by one reviewer (MH). Reviewers did not screen (at title and abstract or full-text) any article to which they were an author. Attempts were made to retrieve full-texts of all articles included at title and abstract screening using the Carleton University library subscriptions or interlibrary loans. Authors of unpublished references or works that were unobtainable through library licenses or interlibrary loans were contacted to gain access to electronic copies. It was not possible to request physical copies of articles not available in electronic form due to COVID-19 public health restrictions at time of searching, which we acknowledge as a potential bias [37].

A consistency check was also done at full-text screening with 12/122 articles [10% of articles included in EPPI Reviewer (which did not include items found through supplemental searches or literature identified by the systematic map)]. After independent review, reviewers (TR and MH) agreed on 83.33% of articles (kappa = 0.57; moderate agreement). Upon discussion, an inconsistency due to a missed detail in one article was resolved. Since this missed detail did not require a different application of the eligibility criteria, final agreement was actually 91.67% (kappa = 0.75; substantial agreement) and full-text screening proceeded. Full-text screening was conducted by a single reviewer (MH). A list of all articles excluded at full-text screening, with reasons for exclusion, is provided in Additional file 3. Articles identified from the systematic mapping exercise were screened for eligibility at the data extraction stage by a single reviewer (MH). Any article excluded during data extraction, along with reasons, is included in the full list of excluded articles (Additional file 3).

### Eligibility criteria

All articles had to meet the following criteria, modified from the systematic map, to be included in the review.

#### Eligible populations

Relevant subjects included any life stage of resident (i.e., non-migratory) or migratory fish, including diadromous species (i.e., fish that migrate between fresh and salt water), in North (23.5° N–66.5° N) or South (23.5° S–66.5° S) temperate regions. Populations could include those that were once stocked (but no longer being actively stocked) or invasive and that are established in the waterbody. Only articles considering fish species in freshwater or estuarine fluvial ecosystems (i.e., water moving via gravity) impacted by HPP systems (such as lakes, rivers and streams), were included.

#### Eligible interventions/exposures

Articles that described a change in, or modification to, the magnitude of downstream flow as a direct result of HPP facilities were included (whether flow varied directly because of hydropower production or due to related operational changes such as spilling for safety or water management). Magnitude, defined here as the amount of water moving past a fixed location per unit time, can be a direct measure of discharge, or expressed as a relative or absolute change [13]. Only downstream fluvial effects of changes to flow magnitude were considered. Changes in, or modifications to, flow magnitude upstream of HPP facilities (e.g., due to impoundment) were not considered. Articles considering other flow component alterations or changes (i.e., frequency, duration, timing or rate of change) were excluded if magnitude was not also considered. Relevant causes of flow alteration included HPP facilities where water moved via gravity (i.e., hydropeaking, impoundment or diversion/run-of-river) or by active pumping (i.e., pumped storage power). Operations that may have impacted flow magnitude, but that were not related to HPP, were excluded. These included but were not limited to: (i) nuclear facilities; (ii) dams without hydropower; (iii) hydrokinetic systems (i.e., energy from waves/currents); and (iv) water withdrawal/diversion systems not associated with HPP. Studies that considered environmental flow augmentation were included if they were associated with HPP facilities. Changes in flow magnitude due to other environmental alterations (i.e., land-use change) or natural causes (i.e., climate change or extreme weather events) without also including the impact of a HPP facility were excluded. Articles with flow magnitude changes due to natural causes were identified for an upcoming systematic review [38] but were not considered in this review. At the request of stakeholders, articles that did not specify a flow component [e.g., the study compared an unregulated stream or stream section to a regulated stream (i.e., regulated via a hydro dam)] or reported unspecified multiple components of flow but did not report the effects separately to isolate individual impacts of the flow components, were included.

#### Eligible comparators

Relevant comparators included: (i) similar sections of the same waterbody with no intervention (e.g., upstream conditions); (ii) separate but similar waterbodies with no intervention; (iii) Before intervention data within the same waterbody (i.e., pre-construction/modification/operation); (iv) alternative levels of intervention on the same or different waterbody; and (v) controlled flume studies (note, no articles of this type were identified during the review process). When authors stated that the comparator was downstream of the HPP site, articles were excluded at the initial data extraction stage to determine a count of this type of article. Based on Advisory Team feedback, we assumed that any site along the full distance of a river experiences the effects of hydropower modification upstream, but with a time delay in relation to upstream sites. Although authors sometimes reported a return to ‘near normal’ flows at downstream control sites, this is not considered to be comparable to control sites that never experience an impact from a HPP system. Therefore, we did not include studies with downstream controls, even if explicitly identified as a control by the authors. Additionally, if upstream comparator sampling sites (i.e., sites in the same waterbody and in upstream conditions) were mostly in free flowing sections of the river, but a minority of sampling sites were in a ‘transition’ zone between the free-flowing section of the river and a reservoir (i.e., reservoir tails), all sampling sites were considered as controls but this study design characteristic was acknowledged during study validity assessment (i.e., the study was assessed as having intervention and control sites that were moderately matched; see Additional file 4: Table S1). If, however, comparator sites were primarily within reservoirs or reservoir tails and no free-flowing sites were considered, these sites could not be considered an upstream comparator and the article was excluded.

#### Eligible outcomes

Included articles considered outcomes that indicated the potential for a change in fish abundance (broadly defined to include fish biomass). Outcomes included those related to: (i) abundance: abundance (number of individuals), density (number of individuals per sampled area), catch per unit effort (CPUE), number of eggs (when considered an age class and not part of a spawning event) and presence/absence, and (ii) biomass: biomass and yield. Fish passage studies that determined the number of fish passing a particular HPP system were included only if they also considered abundance measured below the HPP facility in relation to a change in flow magnitude (i.e., measured numbers or types of fish before and after a flow change below the HPP facility). Passage studies that reported changes in number of individuals above and below the HPP facility or a downstream barrier and used these counts as indicators of fish passage (i.e., the difference in number of fish above and below a natural barrier before and after a change in flow) were excluded as it was not possible to determine if this was a true change in population abundance or simply a change in the number of fish moving from one site to another. Articles were also excluded if they only considered other direct responses of fish productivity (e.g., growth, survival, migration) or evaluated indirect links between measured outcomes and altered flow magnitude (e.g., growth of aquatic plants) and potential responses of fish (e.g., diversity).

#### Eligible types of study designs

This review considered primary, field-based studies including quantification of fish abundance and biomass outcomes using Before/After (*BA*), Control/Impact (*CI*), Before/After/Control/Impact (*BACI*), Reference Conditional Approach (*RCA*), Normal Range (NR), or Randomized Controlled Trials (*RCT*; e.g., small in-field manipulations). Also considered were *CI* designs comparing two levels of intervention on different water bodies (*ALT-CI*) and *CI* designs using a gradient of intervention intensity that included a “zero-control” site (i.e., unimpacted site) (*CI*-gradient). *CI*-gradient studies were originally considered for inclusion but were either converted to: (i) *CI* designs with pseudoreplication, if there were sub-samples taken in the same river, or (ii) multiple, but non-independent CI studies, if studies compared multiple independent rivers with different HPP impacts to a “zero-control” site. Studies were excluded if they used: (i) temporal trends looking at the relationship/correlation between fish abundance or biomass and changes in flow magnitude across time without a ‘true’ Before intervention time period; (ii) spatial trends that do not include “zero-control” site: (a) across waterbodies [e.g., survey fish abundance in six different streams (i.e., of different morphology) and relate to flow magnitude]; or (b) within a waterbody [e.g., survey of fish abundance in different sections of the same stream that differ in morphology (e.g., riffle and run), or where downstream comparators were considered]; (iii) > 1 After-treatment time periods but no change/modification to flow magnitude occurred across time periods [i.e., repeat visits with no Before-treatment; After-only (*A*-only)]; (iv) > 1 impact sites but no change in flow magnitude across impact sites occurred [i.e., multiple impact sites but no control site or Before-treatment data; Impact-only (I-only)]; (v) a single point in time with no comparison to another site; or (vi) a single impact site with no Before-treatment data. Theoretical modeling, reviews and policy discussions were excluded.

#### Language

Only English-language literature was included during the screening stage.

### Study validity assessment

All studies found to be relevant to this review at the full-text screening stage underwent a study validity assessment using a critical appraisal tool, informed by previous tools (e.g., Macura et al. [39]; Martin et al*.*, [40]), developed specifically for this review (see Additional file 4 for further details). Each study (see definition in Table 2) was critically appraised for internal validity (i.e., susceptibility to bias) and study clarity using the predefined criteria outlined in Additional file 4: Table S1. The appraisal tool was made in consultation with the Advisory Team to ensure that it incorporated the components of a well-designed study. External validity (study generalizability) was not directly assessed; instead, generalizability was captured during the screening stage, during data extraction or otherwise noted as a comment in the critical appraisal tool. In accordance with CEE guidelines [34], reviewers would not have assessed study validity or conducted critical appraisal on studies for which they were an author; however, this situation never arose.

**Table 2:** Definition of terms used throughout the systematic review

Term	Definitions
Article	An independent publication (i.e., the primary source of relevant information). Can be from commercially published or grey literature sources. Used throughout the review
Site	A specific hydroelectric facility (i.e., hydro dam) where observations or experiments were conducted and reported in one or more articles. Used throughout the review
Study	An experiment or observation that was undertaken over a specific time period at particular sites reported as separate waterbodies that were not treated as replicates within a single article. Used throughout the review
Project	Individual investigations within a study that differ with respect to ≥ 1 aspect of the study validity criteria (e.g., replication). Used in the review descriptive statistics and narrative review
Case	Situationally defined in text/visual aids (e.g., separate counts of fish life stages) within an independent study. Used in review descriptive statistics and narrative review
Dataset	(1) A single independent study from a single article; or (2) when a single independent study reported separate relevant comparisons for the same or different species and different: (a) operating conditions (e.g., different flow magnitudes/intensities, operational regime); (b) outcome categories (i.e., biomass or abundance); (c) life stages for the same outcome category (e.g., the abundance of eggs for species X and the abundance of age-0 for species X) but otherwise with the same meta-data; (d) outcome metrics within a particular outcome category (i.e., abundance and density or CPUE; or biomass and yield) but otherwise the same meta-data; (e) sampling methods but otherwise with the same meta-data; (f) years and/or seasons post-treatment within a given outcome category (i.e., if for a given outcome category, multiple after time periods were monitored and reported separately for a *CI* study design or within-in year variation post-treatment for a *BA* design), and/or (g) sites downstream of a hydro dam within a single river sampled using a *BA* design but otherwise the same meta-data. The number of datasets was considered during quantitative analysis

Study validity assessment took place at the same time as data extraction and was performed by a single reviewer (MH). A consistency check on the meta-data extraction/quantitative data extraction and study validity assessment was conducted by two reviewers (MH and TR) on 5/103 articles (5%) and quantitative data extraction was further tested on an additional three articles as extraction criteria were refined during data extraction. Meta-data extraction and study validity assessment were done by both reviewers and discrepancies were discussed. When necessary, refinements to the meta-data extraction sheet and validity assessment tool were made to improve clarity of coding and the criteria (additions made to the tool since the protocol are outlined in Additional file 4: Table S1). No study was excluded on the basis of study validity assessments; however, a sensitivity analysis was carried out to investigate the influence of study validity categories (see “Sensitivity analyses”).

### Data coding and extraction strategy

#### General data-extraction strategy

All articles identified from the search update that were included on the basis of full-text assessment underwent meta-data extraction. Articles identified as potentially relevant from the mapping exercise were further screened at this stage; if an article met the full eligibility criteria for this review, it underwent meta-data extraction. If an article was not deemed relevant, it was excluded from the review and recorded with the list of articles excluded at the full-text screening stage, along with reasons (Additional file 3). Data extraction was conducted with a review-specific data extraction form (Additional file 5), following the general structure of our PICO framework. The following key variables of interest were developed through consultation with the advisory team: (i) bibliographical information; (ii) study location and details (e.g., geographic location, waterbody name and type); (iii) hydropower facility information (e.g., type, size, operational capacity); (iv) broad study objective; (v) study design and length; (vi) intervention/exposure (see Table 3 for definitions); (vii) comparator type; (viii) potential confounders (e.g., alterations to other flow components); (ix) outcome type; (x) sampling method(s); (xi) species (or species groups; common and Latin names crosschecked with FishBase [41] or Eschemeyer’s Catalog of Fishes [42]) and life stage(s) studied; and (xii) study validity assessment decisions. Coding within these key variables was based on codes previously developed during the systematic map [33] and expanded through a partially iterative process as options were encountered during scoping and extraction.

**Table 3:** Types of interventions, flow magnitude alterations considered (including elements and direction) and their definitions

Intervention	Description	Code**
Alterations to flow magnitude elements due to hydropower	Any change in the amount of water moving past a fixed location per unit time (e.g., m^3^/s), separated into four general elements:	
	1.Peak flow (reported as alterations in flood, peak, or high flow)	Peakflow
	2.Base flow (reported as alterations in base flow*, low flow or drought conditions)	Baseflow
	3.Average discharge (reported as alterations in total flow or mean flow for any time period)	AvgDischarge
	4.Short-term variation (reported as a change in magnitude that occurred over a period of hours or less than a day)	ShortVar
	5.Unspecified (no specified flow magnitude or direction of change [e.g., the study compares an unregulated stream (or section of a stream) to a regulated stream (i.e., regulated via a hydro dam)]; 2) reported as unspecified multiple flow magnitude elements and flow magnitude direction (i.e., do not report effects of elements separately to isolate individual impacts of flows magnitude elements)	UNSPEC
Increase flow magnitude	An increase in any flow magnitude element, either qualitatively or quantitatively reported by authors (e.g., increase in peak flow)	_Inc
Decrease flow magnitude	A decrease in any flow magnitude element, either qualitatively or quantitatively reported by authors (e.g., a change from 5 m^3^/s base flow to 3 m^3^/s base flow)	_Dec

*Base flow: Here used as a hydroelectrical operational term describing a minimum percentage of average flow, or the minimum allowable flow release from the hydropower facility, regardless of flow required for power generation needs. Our definition does not include baseflow from groundwater sources

**Each intervention is a combination of flow magnitude alteration and direction (e.g., Peakflow_Inc or Baseflow_Dec)

Although we attempted to extract quantitative data on flow magnitude alterations (e.g., Δ change in flow magnitude) the complexity and variation of flow magnitude alterations in the eligible studies made extracting comparable quantitative intervention data impracticable due to limitations in time and resources. Based on stakeholder input, we generalized and characterized flow magnitude alteration by assigning categorical descriptors that capture both the primary change in flow magnitude element [i.e., changes to base flow, peak flow, average discharge or short-term variation (see Table 3 for definitions)] and the general direction of change (e.g., increase or decrease). Although this did not allow us to capture the amount of change in flow magnitude (i.e., the difference between two measures of flow magnitude), it did allow us to consider the impact of the direction of flow magnitude alteration and the type of flow magnitude change (i.e., changes to peak flow, base flow etc.).

Attempts were made to identify supplementary articles (i.e., articles that reported data that could be found elsewhere, that contained portions of information that could be used in combination with another more complete source, or articles that were yearly continuations of a previously established study) and combine them with the most comprehensive article (i.e., the primary source) during data extraction. Although separate laboratory experiments (flume studies) were originally considered potentially relevant, no laboratory experiments were identified during screening. When alternative *CI* studies occurred (*ALT-CI*), a comparator that was most similar to other *CI* studies with “zero-control” sites (i.e., natural or free-flowing rivers or stream sections) was selected by the reviewer. In the one study where this occurred (i.e., Göthe et al. [23]), the systems altered by hydropower that had regulated minimum discharges were considered the comparator (because they were most similar to an unimpacted system) while river systems altered by hydropower that had no regulated minimum discharges were considered the intervention. This enabled us to include *ALT-CI* studies during quantitative analysis while ensuring that the direction of expected change was similar to other *CI* studies.

Additionally, all articles included on the basis of full-text assessment underwent quantitative data extraction when possible. No study was excluded from quantitative data extraction based on study validity. Sample size (i.e., number of rivers or sites within a single river) and outcome (reported abundance or biomass metrics) were extracted as presented in tables or text. When studies reported outcomes from multiple sites within comparator or intervention (i.e., different waterbodies or waterbody sections), we averaged these results to obtain a single value. When multiple sampling years (i.e., different After years in a Before/After study) or seasons (i.e., sampling seasons within a *CI* study design) were reported separately, we extracted each separately. Data from figures were extracted using the data extraction software WebPlotDigitizer [43] when necessary, or authors were contacted to request access to data not otherwise accessible in figures or supplementary files.

#### Data extraction considerations

Following full-text screening of articles by the review team, relevant studies and datasets were extracted from included articles (see Table 2 for definitions). For full details of considerations made in defining our database of information during data extraction, see “Data extraction considerations” in Additional file 6.

Two types of replication within studies (i.e., group sample size) were considered separately to make use of as much data during quantitative synthesis as possible. First, spatial replication in Control/Impact studies was considered at two levels: (i) independent intervention areas (i.e., separate waterbodies receiving treatments—true replicates) and (ii) sub-sampled data within rivers, referred to as pseudoreplicates (e.g., multiple samples made upstream and downstream of a dam). Pseudoreplicates have reported variances for the variability among sub-samples within a true replicate, rather than the variability among true replicates. For true-replicates, we recorded the number of independent intervention and comparator rivers at the level of true replication, while for pseudoreplicates, we recorded the number of pseudoreplicated samples at the plot or sub-sample level within the intervention and comparator areas of a single river (i.e., non-independent replicates). We accounted for pseudoreplicated data by making appropriate adjustments during quantitative synthesis (see “Adjustment accounting for pseudoreplication”; Additional file 7).

Second, temporal replication (i.e., for Before/After intervention study designs) was treated separately (see “BA data extraction considerations” for further details; Additional file 6). Temporal replication was considered here since no *BA* studies included spatial replication (i.e., used a *BA* study design with > 1 replicate waterbodies). If only spatial replication was considered for quantitative synthesis, all *BA* studies would have been ineligible for meta-analysis due to lack of replication. Because of this difference in replication between *CI* and *BA* study designs, separate quantitative analyses were conducted for each type of replication (see “Quantitative synthesis”). Temporal replication was considered at two levels: (i) within-year (n = # months), and (ii) interannual (n = # years). For within year variation, each After year was extracted as a separate row (i.e., different datasets from the same study), with the mean fish outcome and variation for each After time period coming from within-year sampling (e.g., averaged across sampling months or seasons). If fish were sampled for only one Before year (but for > 1 month or season), that Before within-year mean and variation were used as the comparator for each separate After year. If there were multiple within-year time periods (i.e., > 1 year and each year fish were sampled in > 1 month), we used the most recent Before time period (within-year mean and variation) and recorded this for each separate After period. We accounted for multiple comparisons to the same Before year during quantitative analysis. When fish outcome data were available for more than one year in a Before/After design, interannual replication and calculation of interannual variation allowed us to include these data in separate analyses even if no usable information was available on within-year variation (i.e., when a single fish sampling period occurred per year over > 1 years, or when only a total fish abundance for multiple within-year sampling periods was reported for > 1 years). Treating within-year and interannual variation separately ensured we did not introduce bias by considering only interannual variation, if within-year and interannual variation differed. Calculations of interannual variation followed two scenarios. If (a) fish outcomes were only sampled once per year, or (b) studies only reported total fish abundance from multiple sampling seasons within a given year, then mean fish abundance and variation were calculated by averaging these data across all Before years (n = # Before years), and all After years (n = # After years). Alternatively, if fish abundance was sampled/reported more than once per year, average abundance was calculated per year (or used in the case where authors reported this average), then averaged across all Before years (n = # Before years) and all After years (n = # After years). In the latter case, we were able to make use of studies that reported average fish abundance (from multiple within-year samples) but did not provide any information on within-year variation which would have precluded inclusion in the within-year variation analysis above.

#### Data extraction consistency checking

As described previously (see “Study validity assessment”), to ensure meta-data coding, quantitative data extraction and study validity assessments were extracted in a consistent manner, two reviewers (MH and TR) independently piloted the extraction form by coding and assessing information from 5/103 of the same articles (5%) at the beginning of the process. An additional three articles were used to further test quantitative data extraction. Any disagreements (e.g., what constitutes a high, low or very low head dam; see Additional file 5 for definitions) were discussed and additional detailed guidance was added to the extraction codebook to improve clarity. Coding proceeded with one reviewer (MH) and any queries were discussed with a second reviewer (TR) and a consensus decision made. If a decision could not be reached by the two reviewers (MH and TR) uncertainties were discussed and reconciled with the broader research team and refinements to the coding were made in the extraction codebook as required. Reviewers did not extract data from any study on which they were an author.

### Potential effect modifiers and reasons for heterogeneity

For all articles included on the basis of full-text assessment, we recorded information on the following key sources of potential heterogeneity, if available:

- waterbody type (e.g., river, estuary or canals and diversion channels),
- dam size (i.e., high, low or very low head),
- hydropower operational regime (i.e., run-of-river/modified run-of-river, storage or peaking),
- direction of flow magnitude alteration (i.e., increases/decreases in average, peak, base flow magnitude, increases/decreases in short-term variation, and any combination of these changes in flow magnitude; see Table 3 for types of changes and their definitions),
- alterations to other flow components (i.e., frequency, duration, timing, rate of change, or surrogates of flow alteration, or any combinations of alterations),
- sampling methods [i.e., active or passive gear (electrofishing, net samples, trapping), angling, telemetry, mark-recapture, visual, passive integrated transponders (PIT tags) or others],
- sampling seasons,
- type of comparator [temporal or spatial (upstream of dam, no hydropower—separate but similar waterbodies without HPP, or alternative hydro—separate but similar waterbodies with a different HPP regime)],
- time since intervention (years),
- monitoring duration (years), and
- life stage [i.e., egg: eggs, nests and redds; larvae: larvae, alevins, free embryos; age-0: fry, parr (0 +), age-0 + , YOY; juveniles: age-1 + , parr (1 +), juvenile, fingerling (if specific developmental stage is not identified), smolt; adult: adult, spawner, kelt; mixed: assorted life stages].

Potential effect modifiers were selected in consultation with the Advisory Team. When sufficient data were reported and sample size allowed, these potential modifiers were used in meta-analysis (see the “Quantitative synthesis” section) to account for differences among datasets via subgroup analyses or meta-regression (see Table 2 for definitions of terms such as datasets).

### Data synthesis and presentation

#### Descriptive statistics and narrative synthesis

All relevant studies included on the basis of full-text assessments were included in a database providing meta-data on each study. All meta-data were recorded in a MS-Excel database (Additional file 5) and were used to develop descriptive statistics and narrative synthesis of the evidence, including figures and tables. No studies were excluded from narrative synthesis based on study validity.

#### Quantitative synthesis

##### Eligibility and initial data preparation for meta‑analysis

Despite inclusion in the database, some studies were considered unsuitable for meta-analysis and were not included in the quantitative synthesis. These were studies that: (i) lacked replication in the intervention and/or comparator group (i.e., either spatial or temporal); and (ii) did not report measures of outcome variability (i.e., for calculated medians) and/or data on sample sizes and these data could not be otherwise calculated. When possible, imputation (i.e., replacing missing data with calculated substitute values) was used to calculate missing variances (see Additional file 7). Additionally, when only presence/absence data were available for a study or dataset, the study could not be used for quantitative analysis but was retained for the narrative synthesis. Measures of variability were converted to standard deviations when not reported as such (e.g., standard error or confidence intervals) using RevMan Calculator [44].

##### Data preparation—Combining data across multiple comparisons within a study

To reduce multiple effect size estimates from the same study and avoid giving studies with multiple estimates more weight in analyses, select datasets from a given study were aggregated (see Additional file 7 for full description). These aggregations occurred in five instances when studies sharing all other meta-data reported: (i) responses from multiple life stages separately within the same outcome (e.g., the abundance of eggs for species X and the abundance of age-0 for species X, separately) (seven studies); (ii) years and/or seasons post-treatment within a given outcome category [i.e., if for a given outcome category, multiple seasons were monitored and reported separately for a *CI* study design (one study; not included for meta-analysis due to lack of replication) or within-year variation post-treatment for a *BA* design] (four studies); (iii) different sampling methods (no studies), (iv) sites downstream of a hydro dam within a single river sampled using a *BA* design (13 studies), and (v) for one study in which data for both resident and non-resident individuals of the same species were reported. Given one of our objectives was to determine whether generalized fish-flow relationships could be identified from the available literature [which would include null hypothesis testing regarding heterogeneity parameters (e.g., *Q* test to determine whether individual effect sizes estimate a common population mean)], and our small database of studies for quantitative analysis in *CI* and *BA* replication type subsets, we were limited in our ability to use other approaches such as robust variance estimation or multi-level meta-analysis [45–49].

##### Data preparation — Handling dependence from multiple group comparisons

In our database of effect sizes, there were a few instances of multiple group comparisons whereby related studies used a single group of control sites and more than one operational regime [e.g., peaking, run-of-the river, storage (two studies each)] or had multiple interventions comparable to both the initial Before period and to previous After periods (two studies). In some cases, a single comparator site upstream of a diversion and outflow reach was compared to these two intervention types (four studies). In such cases, the comparator group was used to compute more than one effect size and, in consequence, the estimates of these effect sizes are correlated. Because we were interested in testing for the association between operational regime and effect size, we did not aggregate multiple group comparisons within a single study across these regimes. To reduce such case dependencies, we would have removed datasets for a given operational regime where there were insufficient combinable data (i.e., < 3 datasets from < 2 sites); however, this did not occur so no datasets were removed. For cases of multiple group comparisons, we performed sensitivity analyses to compare models fitted with and without such cases to examine differences in pooled effect sizes.

##### Effect size calculation

Because outcomes (e.g., abundance, density, CPUE, or biomass, yield) were not always reported in comparable units or on the same scale, we used the standardized mean difference, Hedges’ *g* [50], as our effect size measure rather than raw mean differences. Hedges’ *g* was calculated using the steps in Borenstein et al. [51], as shown below.

Starting with Cohen’s $d$ to take account of differences in measurements across studies [52], we calculated the standardized mean difference by dividing the mean difference in each study [i.e., the difference between mean fish responses to an intervention and the mean fish response to a lack of intervention (the comparator)] by the study’s pooled standard deviation:

1 $$d=\frac{{\overline{X} }_{G2}- {\overline{X} }_{G1}}{{S}_{\mathrm{pooled}}}$$

where ${\overline{X} }_{G1}$ was the mean of the group 1 ($G1$= the comparator group) and ${\overline{X} }_{G2}$ was the mean of group 2 ($G2$= the intervention group). ${S}_{pooled}$ was the pooled standard deviation of groups 1 and 2:

2 $${S}_{pooled}= \sqrt{\frac{\left({n}_{G2}-1\right){S}_{G2}^{2}+\left({n}_{G1}-1\right){S}_{G1}^{1}}{{n}_{G1}{+n}_{G2}-2}}$$

where $S$ = standard deviation, and $n$ is the sample size. The variance for $d$ is given by:

3 $${V}_{d}=\frac{{n}_{G1}+{n}_{G2}}{{n}_{G1}{n}_{G2}}+\frac{{d}^{2}}{2\left({n}_{G1}+{n}_{G2}\right)}$$

Then, to convert Cohen’s $d$ to Hedges’ $g$, we used a correction factor ($J$) that decreases small sample bias in $d$:

4 $$J=1-\frac{3}{4\left({n}_{G1}+{n}_{G2}-2\right)-1}$$

Finally, we calculated Hedges’ $g$ and the associated variance (${V}_{g}$) as:

5 $$Hedge{s}^{\mathrm{^{\prime}}}g=J \times d$$

6 $${V}_{g}= {J}^{2} \times {V}_{d}$$

From this, a negative Hedges’ $g$ indicates that fish outcomes (abundance or biomass) are lower after the intervention (i.e., sites impacted by a change in flow magnitude or the After period of a Before/After study), than in the associated comparator (i.e., sites unimpacted by a change in flow magnitude or the Before period of a Before/After study). Adjustments were made to these equations when conducting calculations for studies with pseudoreplicates (see “Adjustments accounting for pseudoreplication” in Additional file 7). All effect size calculations were done in MS Excel.

##### Quantitative synthesis

All meta-analyses (i.e., random effects and mixed-effects models) were conducted in R 4.0.3 [53] using the *rma.mv* function in the metafor package (2.4-0) [54]. To determine if changes in flow magnitude had an effect, on average, on fish abundance and biomass outcome metrics, fish responses were compared to controls by conducting random-effects meta-analyses using restricted maximum-likelihood (REML) to compute weighted summary effect sizes for each outcome (i.e., abundance and biomass) within a given replication type (i.e., spatial replication for *CI* study designs, within-year and interannual temporal replication for *BA* study designs) separately. A random-effect model assumes that there is no true effect size that is fixed for all studies and instead assumes that effect sizes will not be identical across studies and that the effect sizes are a random sample from a population of effect sizes [55, 56].

For within-year *BA* comparisons, models were developed for each of the first four years after a change in flow magnitude [i.e., comparing the most recent or only Before year with (i) After year-1 only, (ii) After year-2 only, (iii) After year-3 only, and (iv) After year-4 only], as well as the average of years 1–4 after a change in flow magnitude (see Additional file 7 for full description). The first four years after a change in magnitude were selected since there were insufficient sample sizes in the available evidence base beyond this time frame.

To account for species outcomes reported from the same site but from different studies (see Additional file 7 for full adjustment summary), Study ID was included as a random factor in each model. The summary effect size was considered significantly different from zero when the 95% confidence interval (CI) did not overlap with zero. Heterogeneity in effect sizes was calculated using the *Q* statistic, compared to the chi-square (*χ*^*2*^) distribution to determine if the total variation in observed effect sizes (*Q*_*T*_) was more heterogeneous than expected due to sampling error alone (*Q*_*E*_) (i.e., *Q*_*T*_ is significantly greater than expected from *Q*_*E*_) [57]. A statistically significant *Q* indicates greater heterogeneity in effect sizes (i.e., individual effect sizes do not estimate a common population mean), which suggests there are differences among effect sizes that arise from causes other than sampling error. We produced forest plots to visualize mean effect sizes and 95% CI from each comparison using the *forest* function of the metafor package (2.4–0) [54]. Summary effect sizes were used to identify general trends in the evidence base and the impact of the intervention. It is important to note that a lack of significance does not indicate no significant patterns within the evidence base. Furthermore, a lack of significance can only be interpreted as a lack of evidence for an effect if there is no indication of heterogeneity.

Although we attempted to reduce publication bias by including data from available grey literature, publication bias could still impact results if publishing is biased towards a particular type of result, such as statistically significant outcomes. Therefore, we examined publication bias for global analysis models (as described above) by testing for this bias using funnel plots and fail-safe numbers. Visual assessment of funnel plots (scatter plots of included studies’ effect sizes versus a measure of precision such as sample size, standard error, or sampling variance; Light and Pillemer [58]) was used to determine if bias was present. If no bias was present, the funnel plot should be funnel-shaped, with wider spread of effect sizes with lower precision (i.e., smaller studies) and less spread as precision increases (i.e., larger studies) [58]. We used funnel plots where precision was based on 1/square root of sample size (*k*), because funnel plots based on sample size are less susceptible to distortion than those based on standard error [59]. In these plots, as sample size increases and $1/\sqrt{k}$ decreases, the variance in the effect sizes is expected to decrease if publication bias is not present. Funnel plots were produced using the generic *plot* function in R 4.0.3 [53]. In addition to funnel plots, we used fail-safe numbers to test the robustness of our results against publication bias using the method described in Rosenberg [60], and the *fsn* function in the metafor R package (2.4–0) [54]. Fail-safe numbers indicate the number of nonsignificant, unpublished (or missing) studies needed to eliminate a significant overall effect size [60, 61]. The failsafe number is considered robust if it is greater than $5k+10$, where $k$ is the number of effect sizes in the analysis (i.e., it is unlikely that the number of unretrieved studies is five times that which are considered in the review and the minimum number likely missed is set at 10; Rosenthal [62]).

To test for associations between effect size and moderators, we used mixed-effects models for categorical moderators [i.e., (i) waterbody type, (ii) dam size, (iii) hydropower operational regime, (iv) direction of flow magnitude alteration, (v) alterations to other flow components, (vi) sampling methods, (vii) sampling season, (viii) type of comparator (temporal/spatial), (ix) study class (manipulative vs. nonmanipulative), (x) time since intervention, (xi) monitoring duration (*CI* only), and (xii) life stage] and meta-regression for continuous moderators (i.e., monitoring duration; *BA* only), when possible. We estimated heterogeneity in these models using REML. We only performed analyses for categorical moderators when there were sufficient combinable datasets (i.e., ≥ 3 datasets from at least 2 studies) for each moderator category (e.g., at least 3 datasets from at least 2 studies for each of the operational regimes Peaking and Storage). In some cases, there were insufficient numbers of datasets in different moderator categories; therefore, categories were either combined with similar categories to increase sample size [e.g., when there were insufficient datasets with the same alterations to flow magnitude elements (i.e., Baseflow or AvgDischarge), flow elements were combined into larger groups based on the direction of alteration (increase or decrease)] or datasets were deleted if they did not meet sample size criteria (see details in “Review findings”). For example, studies with comparator sites in waterbodies with alternative levels of hydropower could not be combined with studies where comparator sites were in waterbodies without hydropower; therefore, these datasets were deleted from analyses.

Because studies did not always report all moderators of interest, it was not possible to combine all moderators into a single model simultaneously, nor did sample size allow this. Therefore, to test associations between effect size and moderators, we first conducted random-effects models (unmoderated models) using subsets of responses (e.g., a subset of abundance or biomass effect sizes for a given replication type) that maximized the number of effect sizes that could be used to test the influence of the moderator of interest. We then used these subsets in mixed-effects models or meta-regression, including the moderator of interest. To further account for multiple study comparisons within a study site, and species outcomes being reported for the same site, in all models, Study ID was included as a random variable. We restricted the number of fitted parameters ($j$) in any mixed model such that $k/j$ where $k$ is the number of effect sizes, was greater than five to ensure reasonable model stability and sufficient precision of coefficients [63]. This limited the number of moderators and categories that could be included in a single model. Given that all moderators were highly correlated (see results of Pearson’s ${\chi }^{2}$ test of moderators; Additional file 8: Tables S1 and S2), it was not possible to add more than one moderator into a given model, nor would sample size allow for this.

For all moderator analyses, total heterogeneity (${Q}_{T}$) was partitioned into the heterogeneity explained by the model (${Q}_{M}$) and heterogeneity not explained by the model (${Q}_{E}$, error due to sampling); therefore, ${Q}_{T}={Q}_{m}+{Q}_{E}$. The statistical significance of ${Q}_{M}$ and ${Q}_{E}$ were tested against a chi-square (${\chi }^{2}$) distribution. For *CI* studies, monitoring duration was treated as a categorical variable because of low variability in studies of short duration and few representative longer-term studies (i.e., one study at five years and one study at 36 years duration). Because of two outliers, it was not possible to transform monitoring duration for *BA* studies to meet model assumptions and reduce skewness while maintaining all datasets; therefore, we conducted mixed-effects models including monitoring duration with and without outliers. Results did not differ (see Additional file 8) and we only present results without outliers below.

##### Sensitivity analyses

Sensitivity analyses were carried out to investigate the influence of: (i) study validity categories; (ii) imputing missing variances (i.e., replacing missing data with calculated substitute values); (iii) inclusion of studies where the waterbodies may be influenced by fish stocking; (iv) inclusion of studies with pseudoreplication (i.e., studies where sub-samples were taken in the same river; *CI* studies only); (v) inclusion of multiple group comparisons where a single comparator group was compared to more than one intervention group within the same study and outcome/type of replication subgroup; (vi) inclusion of articles that did not specify a flow magnitude component or reported unspecified multiple components of flow (*CI* studies only); (vi) inclusion of deficient *BA* or *BACI* study designs (*BA* studies only); (vii) inclusion of yearly averages, averaged for the Before or After period (i.e., averages of averages; *BA* studies only), and (viii) inclusion of outflow reaches (i.e., when studies included both areas impacted by diversion and an outflow reach downstream of where water was returned to the system; *BA* studies only). First, models were fit with only those studies assessed as ‘Medium’ validity (see Additional file 4; Table S1). Second, separate models were fit using only studies with variances that did not require imputation during data preparation (see Additional file 7 for more detail). Third, separate models were fit using only studies where stocking was not known to be a potential confounder (i.e., we did not include studies where authors indicated that stocking may have occurred in the system but did not include sufficient information to determine if stocking was ongoing). Fourth, separate models were fit using only studies with true replication for studies with spatial replication (*CI* studies only; see Additional file 6 for more detail). Fifth, separate models were fit with only studies with a single intervention and a single comparator. Sixth, we ran separate models with studies that specified flow magnitude (i.e., we did not include studies that compared an unregulated stream or stream section to a regulated stream). Seventh, we ran separate models (for *BA* studies only) that did not use *DEF_BA* designs. Finally, we ran separate models (for *BA* studies only) that did not include both a diversion and an outflow reach. In all analyses, the results were compared to the overall model fit to examine differences in pooled effect sizes.

## Review findings

### Review descriptive statistics

#### Literature searches and screening

Updated searches of six databases and Google Scholar resulted in 2966 individual records (Fig. 1). Other websites and databases suggested by experts and practitioners identified 1695 individual records. This resulted in 3940 unique records after duplicate removal. A total of 1055 articles remained after title and abstract screening. Of these articles, 17 were not obtainable because of insufficient bibliographical information or articles were not accessible with Carleton University’s subscriptions, leaving a total of 1038 articles for full-text screening. An additional 95 articles from pre-screened sources were included at this stage from searching the bibliographies of (a) relevant articles identified (24 articles), and (b) the 110 relevant reviews found with searches (nine articles), and grey literature sources and submissions obtained via social media/email (62 articles).

Full-text screening removed 922 additional articles, most of which were excluded due to irrelevant study designs (i.e., spatial or temporal trends), comparators (i.e., downstream or lacking comparators) or interventions (i.e., dams without hydro) and were primarily from grey literature sources (763/878 articles; 86%) (see section “Eligibility criteria” for inclusion/exclusion requirements). Of the 211 articles included at full-text screening, 157 were from websites/databases suggested by experts, 24 from reference lists of included articles, 21 from database and the search engine and nine submitted by experts. All articles excluded and unobtainable at full text are listed with an exclusion decision in Additional file 3. An additional 107 articles identified from the Rytwinski et al. [33] systematic map were included for data extraction and screened for inclusion at the data extraction stage.

A total of 318 articles were initially included for data extraction. An additional 146 articles were excluded at this stage, including 67 articles that were supplementary to another excluded article (Fig. 1). A total of 103 articles with 134 studies were included in the narrative analysis for abundance and biomass (see Additional file 5 for a list of included articles). One *CI* study was later excluded prior to narrative and quantitative analysis to ensure independence of studies. Of the remaining 103 articles and 133 studies, all were used in the narrative synthesis and 46 articles with 58 studies were included in quantitative synthesis.

#### Study validity assessment

**Fig. 2:**
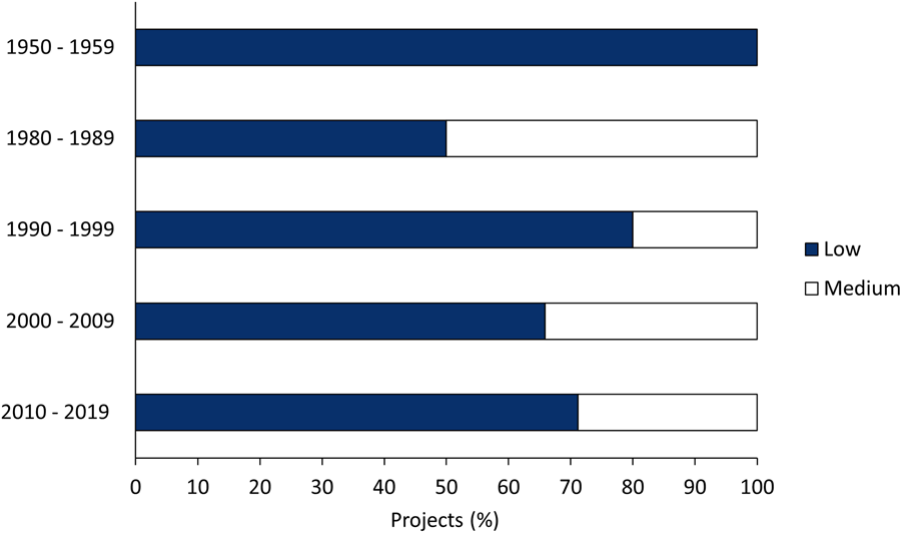
Study validity of 185 projects in relation to the decade of publication, reported as a percentage of all projects for that decade. Projects from 1960 to 1979 were not present in the database

Validity assessments of the 133 studies resulted in 185 individual projects (Additional file 9). Most projects were assigned an overall ‘Low’ study validity (131 projects; 71%), with the remaining projects being assigned an overall ‘Medium’ study validity (54 projects; 29%). No project was assigned an overall ‘High’ study validity. For all decades considered, 50% or more of relevant projects had ‘Low’ validity (Fig. 2).

Among the projects that received an overall ‘Low’ validity score, most (52%) had confounding factors (i.e., manipulations of other flow regime components) or there was a lack of information to judge whether confounders were present. An additional 35% of projects that received a ‘Low’ validity score lacked replication (either spatial and/or temporal). This included studies that: (i) had no replication (29%), or (ii) lacked sufficient information to judge replication (5%). Among projects that received a ‘Medium’ validity score (all other studies), the most common reason (25%) was a lack of true replication (i.e., experimental/observational units were pseudoreplicates) and an additional 12% of projects lacked quantitative measures of flow magnitude alterations. Of the 12 studies (20 projects) that used a *BACI* design, none had a ‘High’ validity score primarily because of insufficient information about the intervention [i.e., no quantitative measure of flow magnitude (nine projects) or compared unregulated to regulated systems but did not specify a change in flow magnitude (10 projects)]. Other reasons for ‘Low’ and ‘Medium’ scores for *BACI* studies were distributed relatively equally in all validity categories. Three *BACI* projects from two *BACI* studies, while having sufficient replication in the intervention zone, lacked sufficient information on interventions and confounders to receive a ‘High’ validity score. These two also lacked sufficient data for the control site before the intervention occurred (i.e., single year of data) to be treated as *BACI* studies in quantitative analysis.

#### Publication year

**Fig. 3:**
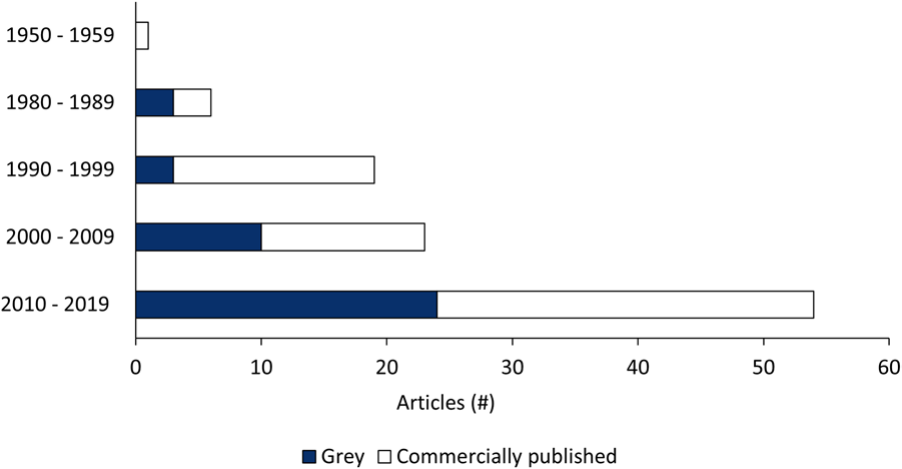
Frequency of grey and commercially published literature considering abundance and biomass and included for data extraction and critical appraisal in each decade. Articles from 1960 to1979 were not present in the database

Articles included for abundance and biomass metrics were published from 1958 to 2019. The number of publications increased over time, with more than twice the number in the most recent decade compared to any previous decade (Fig. 3). From 2000 to 2019, the quantity of grey literature increased and made up just under half (44%) of all articles.

### Narrative synthesis

The narrative synthesis is based on all 133 studies from 103 articles that considered abundance and biomass, regardless of study validity. A database of these studies with descriptive meta-data, coding and qualitative/quantitative data is available in Additional file 5.

#### Study location

**Fig. 4:**
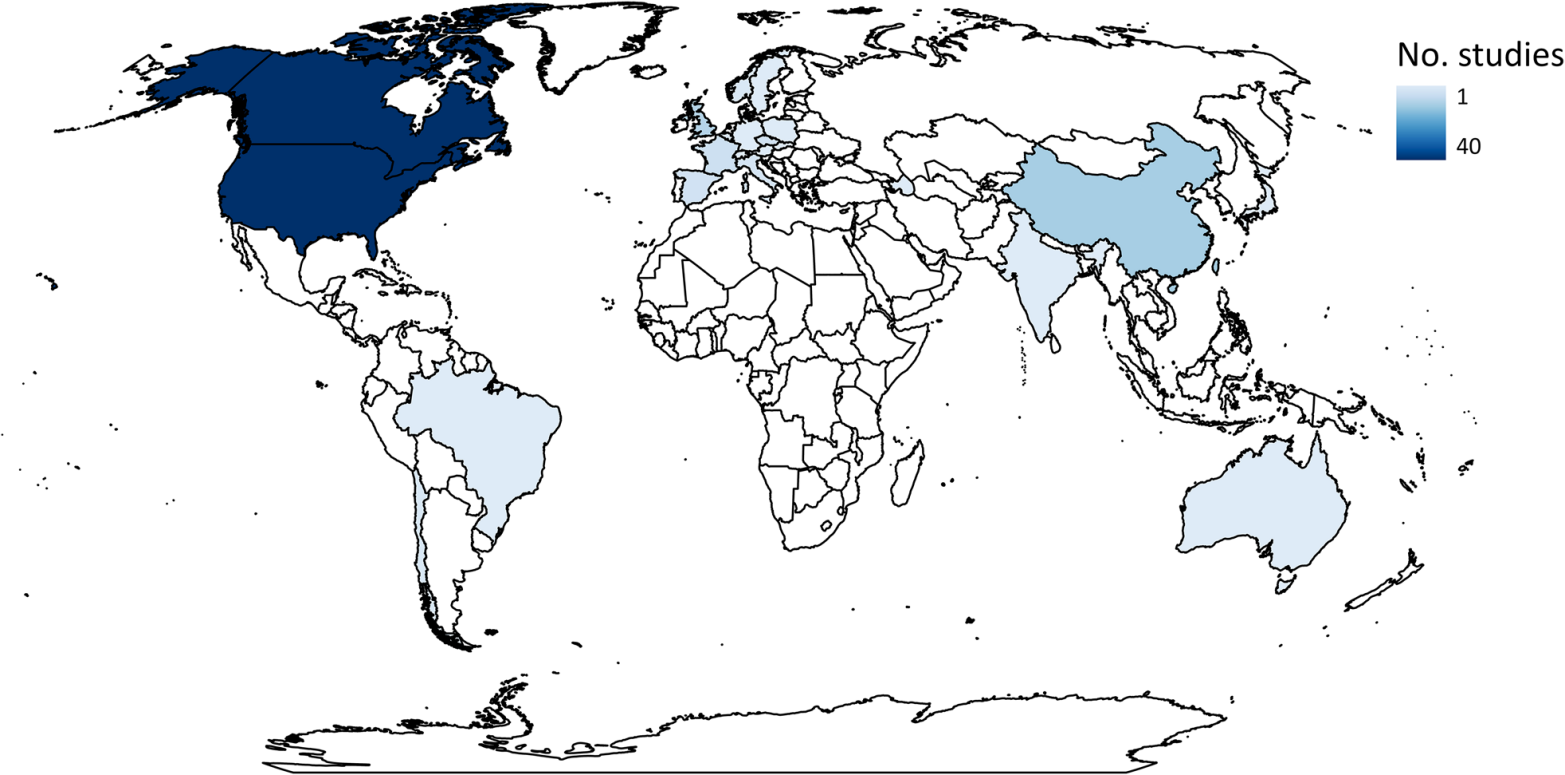
Number of studies considering fish abundance and/or biomass metrics per country

**Fig. 5:**
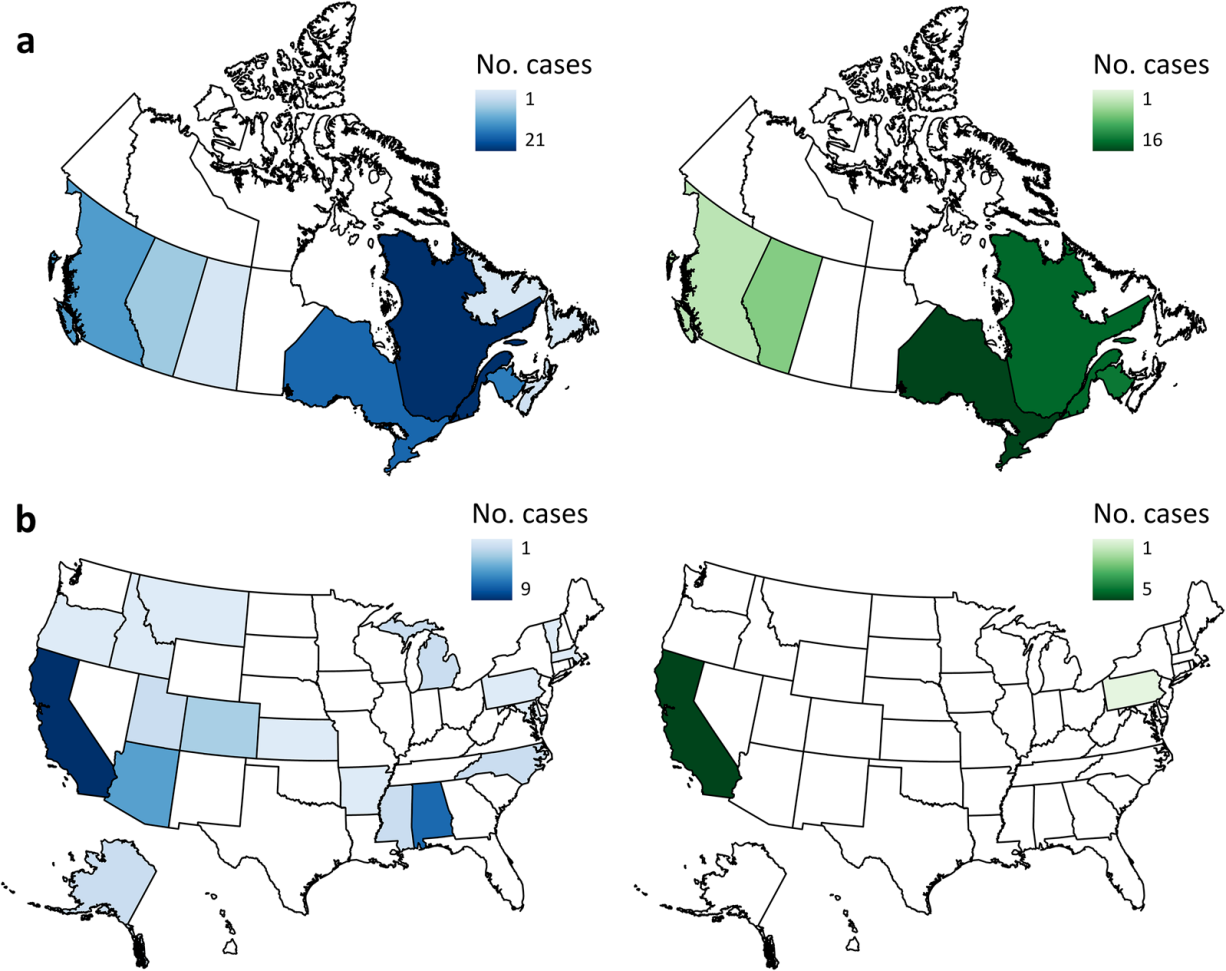
Number of cases considering fish abundance (blue) and biomass (green) metrics per state/province in (**a**) Canada and (**b**) the United States. Note the different colour ranges in each map

Studies occurred in 22 countries (Fig. 4), with most studies conducted in North America (60%); 40 studies were conducted in each of the United States (30%) and Canada (30%). The two most represented Canadian provinces were Quebec and Ontario, respectively, and the most represented American states were California, followed by Alabama (Fig. 5a, b). Some studies had sampling sites in more than one province or state, resulting in more cases than total number of studies. Of the remaining 40% of studies, 26% were conducted in Europe, 10% in Asia, 2% in South America, and 1% in each of Eurasia and Oceania (Fig. 4).

All studies were field based and occurred in river systems, although a single study sampled in both river and estuary environments. Studies reported a total of 111 named hydroelectric power dams/facilities. Several studies considered more than one hydroelectric power dam/facility (i.e., one study considered a total of 17 hydropower facilities and two additional unnamed facilities across 16 waterbodies), resulting in 146 cases. Several studies did not report the name of any dam or facility (23 studies). A total of eight hydroelectric power dams/facilities were included in three or more studies (Additional file 10: Fig. S1), nine were considered in two studies and the remainder occurred in a single study each.

#### Population

**Fig. 6:**
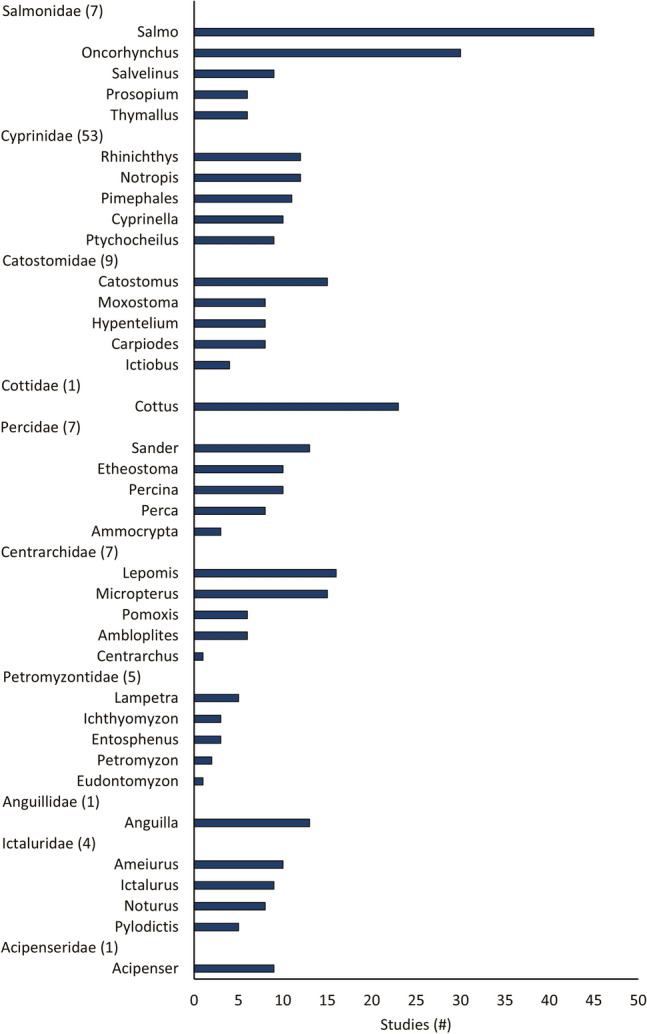
The number of studies per family and genus of the top 10 most studied families, and their associated top five studied genera. The number of genera per family is reported in parentheses adjacent to family name. The number of studies shown exceeds the number of included studies because many studies considered multiple genera

Most studies (75%; 100/133) conducted species-specific investigations (i.e., provided data for individual species rather than grouped/pooled over broader categories of species, genus, or family). Of studies that reported species-specific data, 37% (37/100) considered only one species. A total of 47 families were investigated by studies considering the impact of flow magnitude changes on specific species. This represented 124 genera and 333 species (see Additional file 10: Table S1 for a full species list). Studies also reported four unidentified families, 17 unidentified genera from identified families and five unidentified species from identified genera. The top 10 families and their top studied genera are shown in Fig. 6. All other families were included in fewer than 20 studies each. The most studied species were *Salmo trutta* (41 studies), *Oncorhynchus mykiss* (24), *Micropterus salmoides* (13), and *Salmo salar* and *Lepomis macrochirus* which were reported in 12 studies each.

#### Intervention

**Fig. 7:**
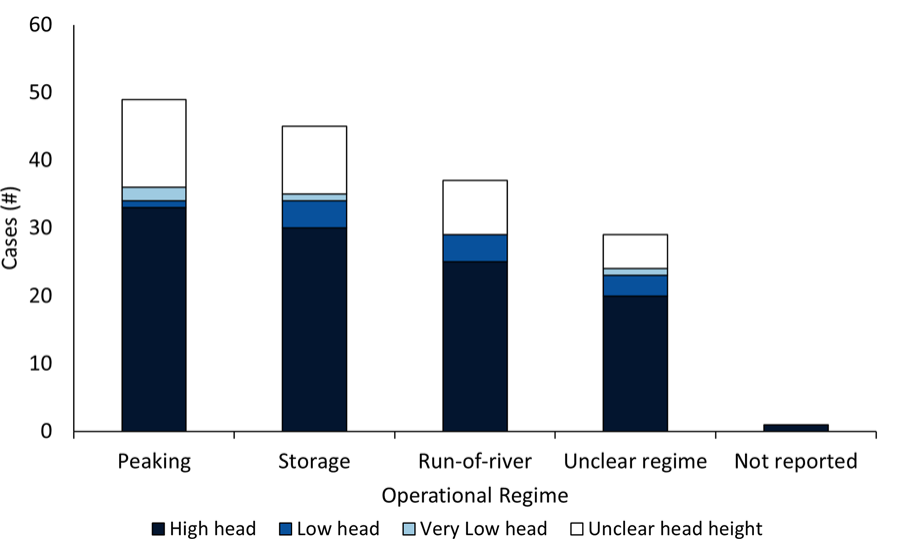
Number of cases of hydroelectric power production facility operations (peaking, storage, run-of-river) in relation to dam head height: high; low; and very low head height. Unclear regime: type of hydropower production facility operation was not clearly enough described to be classed with other operational regimes; Not reported: no information on type of operational regime included

A total of 70 hydropower facilities/dams were reported independently (i.e., no more than one dam considered within the study) and of these seven dams/facilities were considered by more than two studies (Additional file 10: Fig. S2). We separated dam type based on head height and operational regime (i.e., peaking, run-of-river, storage). Due to incomplete reporting in many studies, head height was grouped qualitatively as: (i) ‘high head’ dams (109 cases), (ii) ‘low head’ (12 cases) and (iii) ‘very low head’ dams based on author descriptions (four cases). In 36 cases, no dam head height was included and there was insufficient information to determine the dam head height from other sources. All operational regimes were represented in the database (Fig. 7), although 30 cases did not report an operational regime. One study considered more than one operational regime due to the inclusion of a diversion (run-of-river) and outflow reach (peaking), resulting in 134 cases from 133 studies.

**Fig. 8:**
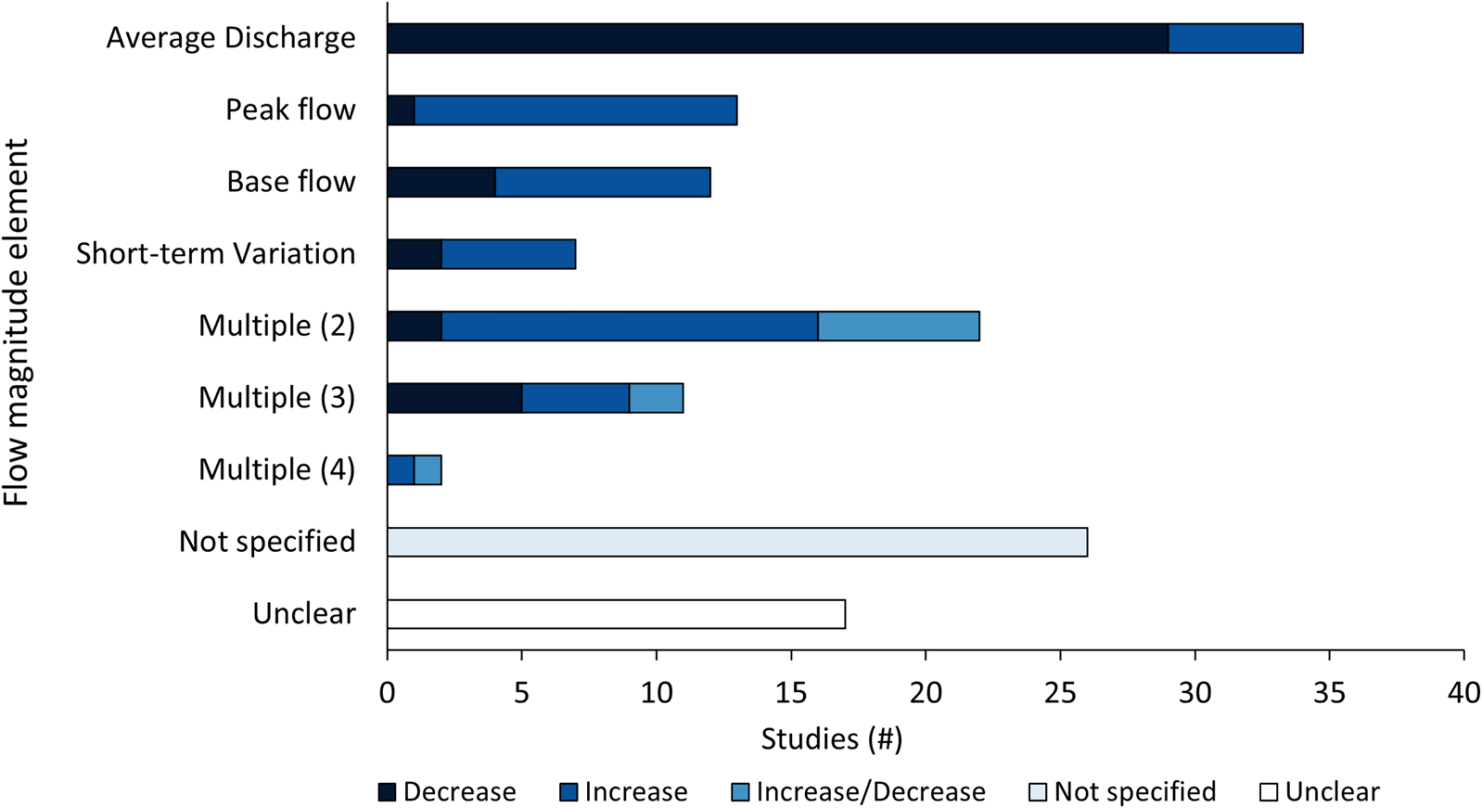
Number of studies with alterations to the four flow magnitude (and their combinations) elements and the direction of alteration (refer to Table 3 for definitions). Multiple indicates that more than one flow element was changed. Flow magnitude elements could be increased or decreased. In cases where multiple changes occurred, individual elements could increase and/or decrease separately (i.e., increase/decrease). Unclear indicates descriptions were provided by authors but were insufficient, while not specified indicates that no descriptions of flow magnitude element or direction of change were provided

Alterations to flow magnitude (when compared to flow conditions normally found in individual systems) were generally increases (49 studies), primarily to peak flow (12 studies) or to two or more flow magnitude elements (19 studies) (Fig. 8; see Table 3 for definitions). A total of 43 studies considered decreases in flow magnitude elements, while 26 did not specify any flow element or direction of alteration (Fig. 8). When flow elements were changed together, this normally resulted in either increasing or decreasing all elements considered, although for eight studies both increases and decreases in flow elements occurred.

#### Study design and comparator

**Fig. 9:**
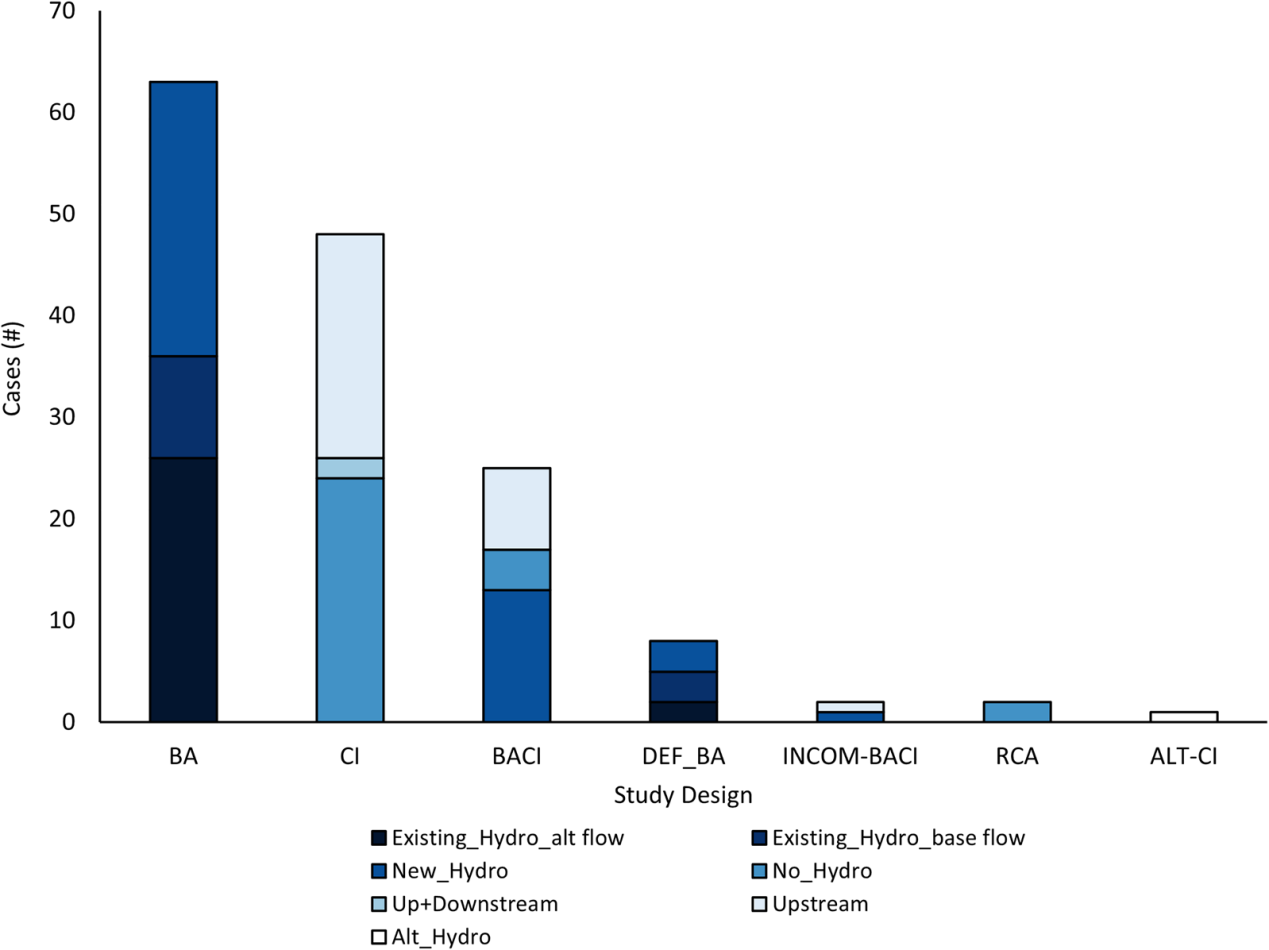
Number of cases by study design and comparator. Study design codes: *BA*: Before/After; *CI*: Control/Impact; *BACI*: Before/After/Control/Impact; *DEF_BA*: deficient Before/After, *INCOM-BACI*: incomplete Before/After/Control/Impact; *RCA*: Reference Conditional Approach; *ALT-CI*: alternative Control/Impact. Temporal comparator codes: *Existing_Hydro_alt flow*: existing HPP where one flow magnitude Before is compared to a new level After intervention; *Existing_Hydro_base flow*: existing HPP where one base flow magnitude Before is compared to a new base flow level After intervention; *New_Hydro*: flow prior to the installation of a new HPP. Spatial comparator: *No_Hydro*: a different nearby waterbody with no HPP; *Upstream*: upstream conditions in unmodified sections of the study waterbody; *Up + Downstream*: both up and downstream unmodified sections of the study waterbody; *ALT_Hydro*: a different nearby waterbody with HPP operating at a different but unmodified flow magnitude. Three studies reported more than one study design; therefore, the number of cases exceeds the number of studies

Most cases had *BA* designs (*BA*: 63 cases; *DEF_BA*: eight cases), followed by *CI* study designs (*CI*: 48 cases, *ALT-CI*: one case), and *BACI* designs (25 cases) (Fig. 9). There were no laboratory, randomized control trials (*RCT*) or normal range (*NR*) studies included.

The most common temporal comparator used by *BA* or *BACI* studies was periods before the installation of a new HPP facility (42 studies), followed by Before periods where altered flow previously existed in the system (41 studies) (Fig. 9). Comparator sites in systems without HPP were most used for *CI* studies (24 cases) and were the only comparator used for *RCA* studies (two cases). *CI* study designs also used upstream comparators (22 cases) and two cases combined upstream/downstream comparators. *BACI* and incomplete *BACI* studies used only Before periods prior to the installation of a new hydropower system (14 cases) but used both upstream comparators (nine cases) or systems without HPP facilities (four cases).

#### Outcomes

**Fig. 10:**
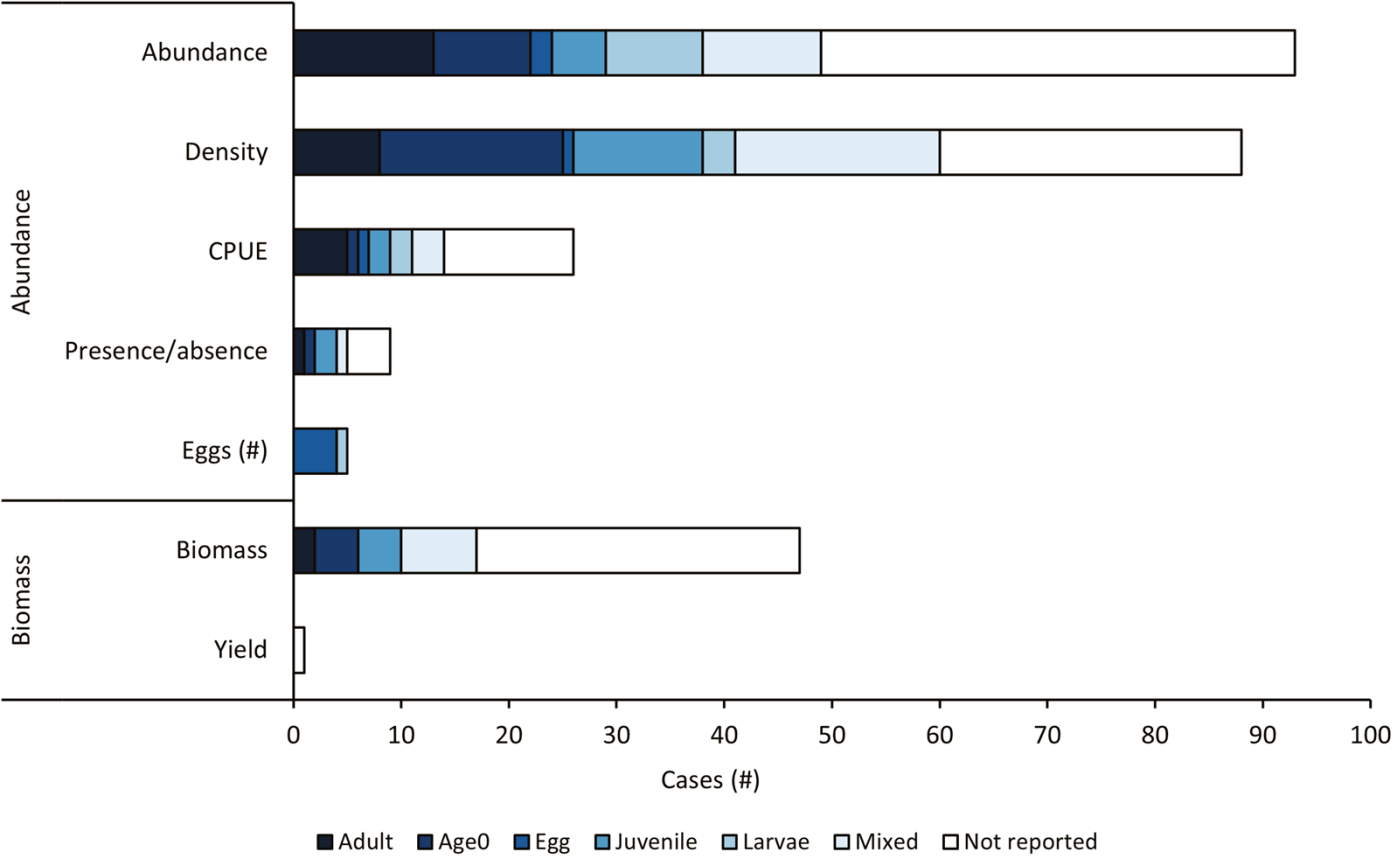
Frequency of reported fish outcomes and life stage. Note: several studies reported more than one outcome and life stage separately. Mixed life stages include any combination of other life stages. *CPUE*: catch per unit effort

Studies often recorded more than one fish response (i.e., abundance and biomass in the same study) or more than one outcome metric (e.g., abundance and density) resulting in 269 cases from 133 studies. Most cases considered abundance outcomes (82%), with the majority using abundance (42%) and density (40%) metrics (Fig. 10). Biomass outcomes (including the metrics of biomass and yield) accounted for 18% of all cases, with only a single case considering yield. The most reported life stages for abundance was age-0 fish (28 cases), and for biomass they were juveniles and age-0 fish (four cases each).

Fish were sampled with a variety of sampling methods with many studies using more than one method (143 cases from 133 studies). The most used method was gear (e.g., electrofishing, gill-, fyke-, seine-netting, trapping; with no mark-recapture techniques) (94 cases). Other methods included: visual techniques (nine cases), angling (five), mark-recapture + gear (three), a combination of techniques (19) or other techniques (i.e., historical or commercial catch data, hydroacoustics; 12 cases). No study used telemetry to sample fish. Some studies sampled different species or life stages with more than one type of method, resulting in 167 cases from 133 studies. Both fish abundance and biomass were sampled primarily with gear (113 cases) or some combination of techniques (32 cases).

Studies generally reported data for a single sampling season (62 studies/133 studies), with 29 sampling in summer, 25 in fall, five in spring and three in winter only. Several studies conducted sampling during more than one season (53 studies), resulting in more cases than the total number of studies (174 cases). Of these studies, 47% sampled in three or more seasons. Summer was sampled in the intervention (i.e., intervention site or After period) in 50% of all cases (considering cases where sampling was conducted in single and multiple seasons together), followed by fall (43%), spring (25%) and winter (11%). Four *BA* studies had mismatched sampling in the Before and After period (i.e., sampled in summer in the After period, but fall in the Before period), while 13 *BA* studies had partial mismatch (i.e., one or more seasons matched, but other seasons were also present in the Before but not the After period).

### Quantitative synthesis

#### Description of the data

Of the 133 studies (from 103 articles) included in the narrative synthesis, 58 studies (from 46 articles) with 268 datasets, after aggregation, were included in our quantitative synthesis database (Additional file 11). Of these, 22 studies (91 datasets after aggregation) were used to analyze Control/Impact studies, while 37 studies (165 datasets after aggregation) were used in analyzing Before/After study designs. A single study had both a *CI* and *BA* component, so the total number studies (59) is greater than the actual number of studies included (58). We combined *CI*, *ALT-CI*, *RCA* and *BACI* converted to *CI* in analyses of Control/Impact studies and combined *BA*, deficient *BA*, incomplete *BACI* converted to *BA* and *BACI* converted to *BA* study designs in analysis of Before/After studies. We intended to analyze the potential for a time lag in *CI* designs when studies reported > 1 post-treatment years, but there was insufficient sample size to allow for such an analysis. These datasets were removed for all subsequent *CI* analyses and analyses proceeded with datasets for the first year post-treatment only.

Of the datasets included in the quantitative synthesis (256), 51% had ‘Medium’ overall study validity (*CI*: 47; *BA*: 83 datasets), while 49% had ‘Low’ overall study validity (*CI*: 44; *BA*: 82 datasets). Most datasets were from North America (182), with the majority from the United States (105/182 datasets), followed by Canada (77). The next most represented region was Europe with 42 datasets from five countries. No datasets were included from South America or Eurasia.

Within datasets with individual species information (226/256), 98 species, from 57 genera and 27 families were evaluated for impacts of flow magnitude alterations. The most evaluated species were from the Salmonidae family, including *Oncorhynchus mykiss* (24 datasets), *Salmo trutta* (24 datasets), *Oncorhynchus kisutch* (10 datasets), *Salmo salar* (10 datasets) and *Oncorhynchus tshawytscha* (eight datasets).

A total of 32 hydropower facilities/dams were included in the quantitative synthesis database (see Additional file 10 for further details of individual facilities/dams in *CI* or *BA* analyses). Information on dam size (i.e., high, low or very low head height) and operation (peaking, run-of-river and storage) was available for 241 and 207 of the total 256 datasets. The most common head height was high head (89%), followed by low head (3%) and very low head (2%). Storage operations were the most common operational regime (40%), followed by peaking (25%) and run-of-river (15%).

Of the 256 datasets, 88% reported fish abundance outcomes, while the remaining 12% reported fish biomass. Both *CI* and *BA* study design datasets primarily reported fish abundance (86% and 90% of datasets, respectively). The range of monitoring duration for all datasets was between less than one and 36 years. Most datasets reported monitoring for three or fewer years (72%, with 21% of these monitoring for ≤ 1 year), while less than 5% of datasets reported greater than 10 years. A single study reported greater than 20 years of monitoring. For further descriptions on flow magnitude element alterations, life stages and sampling methods included in quantitative synthesis, see Additional file 10.

#### Global meta-analyses—Control/Impact studies

##### Abundance

The overall mean weighted effect size of *CI* studies for abundance was − 0.001 (95% CI − 0.35, 0.34; *k* = 77, *p* = 0.997; Table 4A, Additional file 12: Fig. S1), suggesting changes in flow magnitude did not significantly affect fish abundance. Over half of the effect sizes were positive (i.e., *g* > 0; 43 of 77), suggesting that changes in flow magnitude positively impacted fish abundance (i.e., abundance was higher in intervention sites than in control sites) with the remaining showing neutral or negative responses (i.e., *g* ≤ 0) to changes in flow magnitude; however, most of the individual effect sizes were not statistically significant, having confidence intervals that overlapped zero (70 out 77 effect sizes) (see forest plot Additional file 12: Fig. S1). The *Q* test for heterogeneity suggested that there was significant heterogeneity between effect sizes (*Q* = 104.05, *p* = 0.018). Funnel plots of asymmetry suggested possible evidence of publication bias towards larger studies showing positive effects of flow magnitude change (funnel plot Additional file 12: Fig. S2). Interestingly, this evidence of bias appears in the grey literature, not the commercially published literature. The failsafe number (N = 0) was not greater than 5 k * 10 [(5 * 77 + 10) = 395], suggesting the results from the random effects model may not be robust against potential publication bias.

**Table 4:** Summary statistics from the main analyses of abundance and biomass including for Control/Impact, within-year and interannual Before/After studies, and from taxonomic-specific analyses

Analysis	Standardized mean difference (Hedges’ *g*)
*Control/Impact*	
A.Global meta-analyses	
Abundance (*k* = 77)	− 0.001 (95% CI − 0.35, 0.34; *p* = 0.997)
Biomass (*k* = 13)	− 0.16 (95% CI − 0.62, 0.30; *p* = 0.490)
B. Taxonomic analyses	
Abundance	
Catostomidae (*k* = 4)	− 0.46 (95% CI − 1.79, 0.88; *p* = 0.503)
Centrarchidae (*k* = 6)	0.52 (95% CI − 0.83, 1.14; *p* = 0.753)
Cyprinidae (*k* = 19)	− 0.09 (95% CI − 1.20, 1.03; *p* = 0.878)
Percidae (*k* = 4)	0.32 (95% CI − 0.84, 1.49; *p* = 0.590)
Salmonidae (*k* = 5)	0.18 (95% CI − 0.58, 0.93; *p* = 0.646)
Biomass	N/A
*Before/After—within year*	
C. Global meta-analyses	
Abundance—year 1(*k* = 19)	0.25 (95% CI − 0.04, 0.56; *p* = 0.091)*
Abundance—year 2 (*k* = 5)	0.67 (95% CI − 0.09, 1.43; *p* = 0.084)*
Abundance—year 3 (*k* = 4)	0.31 (95% CI − 0.62, 1.23; *p* = 0.516)
Abundance—year 4 (*k* = 3)	0.20 (95% CI − 0.80, 1.20; *p* = 0.697)
Abundance—year 1–4 (*k* = 19)	0.25 (95% CI − 0.02, 0.52; *p* = 0.072)*
Biomass	N/A
D. Taxonomic analyses	
Abundance	
Salmonidae (*k* = 4)	0.81 (95% CI − 0.15, 1.76; k = 4, p = 0.099)*
Biomass	N/A
*Before/After—interannual*	
E. Global meta-analysis	
Abundance (*k* = 112)	0.19 (95% CI − 0.23, 0.61; *p* = 0.374)
Biomass (*k* = 17)	0.46 (95% CI − 0.24, 1.15; *p* = 0.196)
F. Taxonomic analysis	
Abundance	
Acipenseridae (*k* = 5)	0.42 (95% CI − 1.98, 2.81; *p* = 0.733)
Anguillidae (*k* = 5)	− 0.45 (95% CI − 1.44, 0.55; *p* = 0.379)
Catostomidae (*k* = 8)	− 0.38 (95% CI − 1.84, 1.07; *p* = 0.606)
Centrarchidae (*k* = 5)	7.14 (95% CI − 5.68, 19.96; *p* = 0.275)
Cottidae (*k* = 5)	**1.34 (95% CI 0.39, 2.29; *p* = 0.006)**
Cyprinidae (*k* = 15)	− 1.18 (95% CI − 3.26, 0.90; *p* = 0.266)
Esocidae (*k* = 3)	0.37 (95% CI − 0.36, 1.10; *p* = 0.325)
Ictaluridae (*k* = 3)	6.69 (95% CI − 6.86, 20.24; *p* = 0.333)
Salmonidae (*k* = 59)	**0.45 (95% CI 0.25, 0.65; *p* < 0.0001)**
Biomass	
Salmonidae (*k* = 11)	0.52 (95% CI − 0.38, 1.43; *p* = 0.258)

N/A: Unable to conduct moderator analyses due to insufficient sample size or variability. A decrease in fish abundance from alterations to flow magnitude due to HPP compared to control groups is indicated by a value < 0 for Hedges’ *g*

Hedges’ *g* is the standardized mean difference effect size. CI: 95% confidence interval. Bold indicates significant effect (*p* < 0.05); *Indicates marginally significant effect (*p* < 0.1). *k*: number of effect sizes

The sensitivity analyses for both ‘Medium’ validity studies and studies with true replication showed a more negative effect of flow magnitude changes on fish abundance compared to the overall meta-analysis. The difference in the relative magnitude of effect sizes for these analyses suggests that the results may not be fully robust to the inclusion of studies with ‘Low’ validity or pseudoreplication, but the effect sizes were non-significant. Results of all other sensitivity analyses were comparable to the overall meta-analyses (for further details, see Additional file 12: Table S1).

##### Biomass

The overall weighted mean effect size for biomass was − 0.16 (95% CI − 0.62, 0.30; *k* = 13, *p* = 0.490), suggesting that changes in flow magnitude negatively impacted fish biomass (i.e., biomass was lower in intervention sites than in control sites), but the response was not significant (Table 4A). Most effect sizes were negative (i.e., *g* < 0; 7 out of 13); however, only one effect size was statistically significant (see forest plot Additional file 12: Fig. S6). The *Q* test of heterogeneity suggested that there was moderate statistical significance in heterogeneity between effect sizes (*Q* = 20.94, *p* = 0.051). The funnel plot of asymmetry did not suggest an obvious pattern of publication bias; however, it is difficult to determine asymmetry with this small number of studies (*k* = 13; Additional file 12: Fig. S7). The failsafe number (N = 0) was not greater than 5* k* + 10 [(5*13 + 10) = 75], suggesting the results from the random effects model may not be robust against potential publication bias. Results of sensitivity analyses were comparable to the overall meta-analyses (for further details, see Additional file 12: Table S2).

#### Effects of moderators—Control/Impact studies

##### Abundance

When addressing potential reasons for heterogeneity in the results, there was only sufficient sample size (i.e., ≥ 3 datasets from ≥ 2 studies) to address effect-modifying factors for *CI* study designs and abundance. Additionally, there were too few effect sizes (in sufficiently different categorical levels) to allow meaningful analysis of waterbody type (i.e., all included studies occurred in rivers) or ‘life stage’ (only Mixed and Not reported life stages were present). We present the results of all other moderator analyses here and summarize the outputs of conducted moderator analyses for *CI* study designs in Table 5.

**Table 5:** Summary results of meta-analyses using subsets of fish abundance effect sizes for Control/Impact studies, testing the influence of the given moderator variable

Moderator	k	*Q* statistic (*p*-value)	*Q*_*M*_ (*p*-value)	*Q*_*E*_ (*p*-value)
Waterbody type	77	N/A		
Dam size				
Unmoderated model	77	**104.05 (*p* = 0.018)**	–	–
Dam size	77	–	0.52 (*p* = 0.773)	**98.22 (*p* = 0.031)**
Hydropower operational regime				
Unmoderated model	77	**104.05 (*p* = 0.018)**	–	–
Operational regime	77	-	2.85 (*p* = 0.241)	90.12 (*p* = 0.098)*
Direction of flow magnitude alteration				
Unmoderated model	76	**103.24 (*p* = 0.017)**	–	–
Flow alteration	76	–	0.36 (*p* = 0.551)	92.12 (*p* = 0.076)*
Alterations to other flow components				
Unmoderated model	77	**104.05 (*p* = 0.018)**	–	–
Other components	77	–	0.27 (*p* = 0.873)	**99.31 (*p* = 0.027)**
Sampling methods				
Unmoderated model	73	**96.90 (*p* = 0.028)**	–	–
Sampling technique	73	–	0.07 (*p* = 0.789)	**96.29 (*p* = 0.025)**
Sampling season				
Unmoderated model	77	**104.05 (*p* = 0.018)**	–	–
Sampling season	77	–	2.21 (*p* = 0.531)	**94.90 (*p* = 0.044)**
Type of comparator (spatial)				
Unmoderated model	76	**97.46 (*p* = 0.042)**	–	–
Comparator site	76	–	2.04 (*p* = 0.153)	86.01 (*p* = 0.161)
Time since intervention				
Unmoderated model	77	**104.05 (*p* = 0.018)**	–	–
Time since intervention	77	–	1.64 (*p* = 0.441)	88.06 (*p* = 0.126)
Monitoring duration				
Unmoderated model	75	**97.46 (*p* = 0.035)**	–	–
Monitoring duration	75	–	3.88 (*p* = 0.143)	83.97 (*p* = 0.158)
Life stage	77	N/A		

Bold indicates statistical significance (*p* < 0.05)

Unmoderated model: random-effects model; *Significance at *p* < 0.1. N/A: unable to assess moderator due to insufficient sample size or lack of variation

*k*: number of effect sizes; *Q* statistic: value of homogeneity test; *Q*_*M*_: omnibus test statistic of moderators; *Q*_*E*_: unexplained heterogeneity

Due to sample size, we either combined or dropped levels within moderators as follows:

- Dam size (i.e., head height): (i) High; (ii) Low + Very-low; (iii) Unclear.
- Hydropower operational regime: (i) Peaking; (ii) Run-of-river; (iii) Storage.
- Direction of flow magnitude alteration: there was only sufficient sample size to consider two interventions: (i) Increase; (ii) Unspecified. We collapsed any increase of average, peak or base flow and short-term variation into ‘Increase’ and any decrease in average, peak or base flow and short-term variation into ‘Decrease’. However, the single effect size with a decrease in flow magnitude was not included during analysis.
- Alterations to other flow components: (i) Yes (i.e., alterations present); (ii) No; (iii) Unclear + Not reported.
- Sampling methods: (i) Gear; (ii) Multiple (i.e., any combination of methods). Four datasets from a single study using visual sampling methods were not included during analysis.
- Sampling seasons: (i) Summer; (ii) Fall; (iii) Summer + Fall; (iv) ‘Spring + Summer + Fall’ + All seasons.
- Type of comparator (spatial): (i) Upstream (i.e., upstream of dam/facility); (ii) No hydro (i.e., separate but similar waterbodies without HPP). Because it was not possible to combine comparator types, the single effect size with alternative hydropower as a comparator was not included in the analysis. No effect sizes with upstream and downstream comparators were included in the analysis.
- Time since intervention (years): (i) ≤ 1 year; (ii) > 4 years; (iii) Unclear + Not reported.
- Monitoring duration (years): (i) ≤ 1 year; (ii) 2 years; (iii) 3 years; (iv) ≥ 5 years. Due to sample size, we considered monitoring duration a categorical variable in *CI* studies.

For all moderators considered, we found no detectable effect on the average effect size (Table 5). Additionally, most moderators were highly correlated (see results of Pearson chi-square test; Additional file 8: Table S1).

##### Biomass

There were too few effect sizes within biomass and *CI* studies (*k* = 13) to allow for meaningful analysis of potential effect modifiers.

#### Global meta-analyses—Before/After studies

##### Within-year Before/After studies —Abundance

**Fig. 11:**
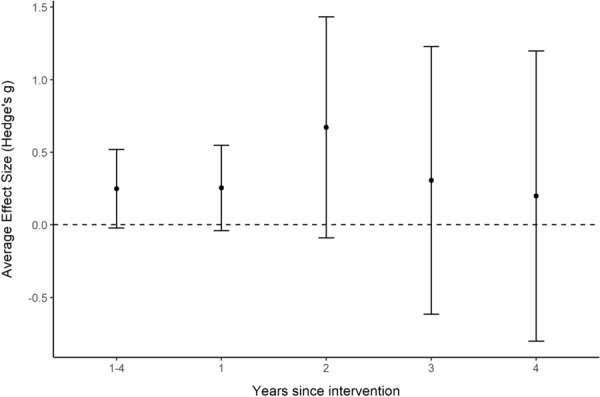
Comparison of overall average effect size for within-year *BA* studies one (*k* = 19), two (*k* = 5), three (*k* = 4) and four (*k* = 3) years post-intervention and when After years 1–4 were aggregated (year 1–4). Error bars indicate 95% confidence intervals. Models were developed for each of the first four years after a change in flow magnitude [i.e., comparing the most recent or only Before year with After year-1 only, After year-2 only, After year-3 only, and After year-4 only, as well as the average of years 1–4 after a change in flow magnitude. 95% confidence intervals that do not overlap with the dashed line indicate a significant effect (at the *p* < 0.05 level). *k*: number of effect sizes

The overall mean weighted effect size of within-year *BA* studies, considering only effect sizes for abundance in post-intervention year-1 was 0.25 (95% CI − 0.04, 0.56; *k* = 19, *p* = 0.091), suggesting changes in flow magnitude had a moderately significant overall positive effect on fish abundance (Table 4C, Fig. 11; Additional file 12: Fig. S8.). However, the sample size was small and there was a single study (with a single dataset) with a much larger, significant positive effect size, which may have disproportionately impacted the mean effect size for increasing abundance [non-native, established *Oncorhynchus mykiss* (Avery et al. 2015); see Additional file 12: forest plot Fig. S8 and Cook’s distance Fig. S10]. Most other effect sizes were also positive (i.e., *g* > 0; 13 of 19), with the remainder showing neutral or negative responses (i.e., *g* ≤ 0) to changes in flow magnitude. These effect sizes were not statistically significant and had confidence intervals that overlapped zero (18 out 19 effect sizes) (see forest plot Additional file 12: Fig. S8). The *Q* test for heterogeneity did not suggest significant heterogeneity between effect sizes (*Q* = 15.65, *p* = 0.617). There was no obvious indication of publication bias from the funnel plot, although it was difficult to determine asymmetry with this small number of studies (Additional file 12: Fig S9). However, the failsafe number was zero suggesting the results from the random effects model may not be robust against potential publication bias. For sensitivity analyses results, refer to Additional file 12: Table S3.

To investigate the potential impact of a time-lag in within-year fish responses to changes in magnitude, we compared the effect sizes for subsequent years of sampling post-intervention to that of the overall mean weighted effect size for year-1 datasets. The overall mean weighted effect size of post-intervention year-2 for abundance was moderately significant 0.67 (95% CI -0.09, 1.43; *k* = 5, *p* = 0.084) indicating that a slight increase in abundance may occur after two years of monitoring (Fig. 11; Additional file 12: Fig. S11). However, the sample size was quite small, and one study had a significant effect size (non-native, established *Oncorhynchus mykiss*; Korman et al. 2011) which may have had a disproportionate effect on the overall mean effect size. The overall mean weighted effect sizes after post-intervention year-3 decreased. By post-intervention year-4, the effect size had returned to a value similar to, but slightly smaller, than those of post-intervention year-1 [post-intervention year-3: Hedge’s *g* = 0.31 (95% CI − 0.62, 1.23; *k* = 4, *p* = 0.516); post-intervention year-4: Hedge’s *g* = 0.20 (95% CI − 0.80, 1.20; *k* = 3, *p* = 0.697)] (Fig. 11; Additional file 12: Fig. S12–13). Of the 15 species present in year-1, only three species were present in all four post-intervention years (*Cottus gobio*, *Oncorhynchus mykiss*, *Salmo trutta*). Year-2 had one species not present in year-3 and -4 (*Sinibotia superciliaris)*. When all post-intervention years (1–4) were aggregated, the resulting overall mean weighted effect size was very similar to that of year-1 alone [Hedge's *g* = 0.25 (95% CI − 0.02, 0.52; *k* = 19, *p* = 0.072)] (Fig. 11), with a moderately significant effect of changes in flow magnitude on fish abundance (see forest plot Additional file 12: Fig. S14). For each post intervention year separately, and for aggregated years 1–4 post-intervention, the *Q* test for heterogeneity suggested that there was no significant heterogeneity between effect sizes. Due to small sample sizes, it was not possible to investigate the effect of moderators for within-year *BA* studies.

##### Within-year Before/After studies —Biomass

No study using within-year temporal replication considered biomass as an outcome metric. Therefore, quantitative analysis for this subset of Before/After studies only considers abundance.

##### Interannual Before/After studies—Abundance

The overall mean weighted effect size for abundance when considering interannual *BA* studies was 0.19 (95% CI − 0.23, 0.61; *k* = 112, *p* = 0.374; Table 4E), indicating that alterations in flow magnitude had a slight positive, but non-significant, overall effect on fish abundance. Most effect sizes were positive (i.e., *g* > 0; 77 of 112), with the remaining 35 effect sizes showing neutral or negative responses (i.e., *g* ≤ 0) to alterations in flow magnitude. Most individual effect sizes were not statistically significant with confidence intervals overlapping zero (89 of 112; Additional file 12: Fig. S16), although 10 datasets had significant negative, and 13 datasets had significant positive effect sizes. The *Q* test for heterogeneity suggested that there was significant heterogeneity between effect sizes (*Q* = 421.12, *p* < 0.0001) that could be explored using mixed effects models (see section “Effects of modifiers—interannual *BA* studies”). The funnel plot of asymmetry suggests possible evidence of publication bias, especially in grey literature [i.e., as study sample size increased, the variance in effect sizes increased (see Additional file 12: Fig. S17)]. Also, the fail-safe number (N = 407) was less than 5 k + 10 [(5*112 + 10) = 570], suggesting the results of the random effects model may not be robust to publication bias. All sensitivity analyses applicable to interannual *BA* study designs had similar results to the overall meta-analysis (refer to Additional file 12: Table S4 for further details).

##### Interannual Before/After studies—Biomass

The overall mean weighted effect size for biomass when considering interannual *BA* studies was 0.46 (95% CI − 0.24, 1.15; *k* = 17, *p* = 0.196; Table 4E) suggesting an overall increase in fish biomass with alterations in flow magnitude compared to Before periods; however, the estimated overall response was not significant (Additional file 12: Fig. S24). Most effect sizes were positive (10/17) while the remaining seven effect sizes were negative. The *Q* test for heterogeneity suggested that there was significant heterogeneity between effect sizes (*Q* = 28.37, *p* = 0.03) that could be explored using mixed effects meta-analysis models; however, given the small number of effect sizes, the influence of categorical moderators could not be assessed due to potential overparameterization, and was not possible for continuous moderators due to lack of variability in the datasets (i.e., there were too few datasets for each year and gaps between years were too large to allow effect meta-regression). The funnel plot for the random effects model did not show any obvious pattern of publication bias, but with the small sample size, determination of asymmetry was difficult (note that no grey literature considered biomass for interannual *BA* studies; Additional file 12: Fig. S25). The failsafe number was two (less than the suggested 5 k + 10 [5*17 + 10 = 95], indicating the results of the random effect model for biomass may not be robust against publication bias. Results of the sensitivity analyses indicated that models may not be robust to the inclusion of studies with more than one intervention, or that report averages of averages (for details, refer to Additional file 12: Table S5).

#### Effects of moderators—interannual Before/After studies

##### Abundance

**Fig. 12:**
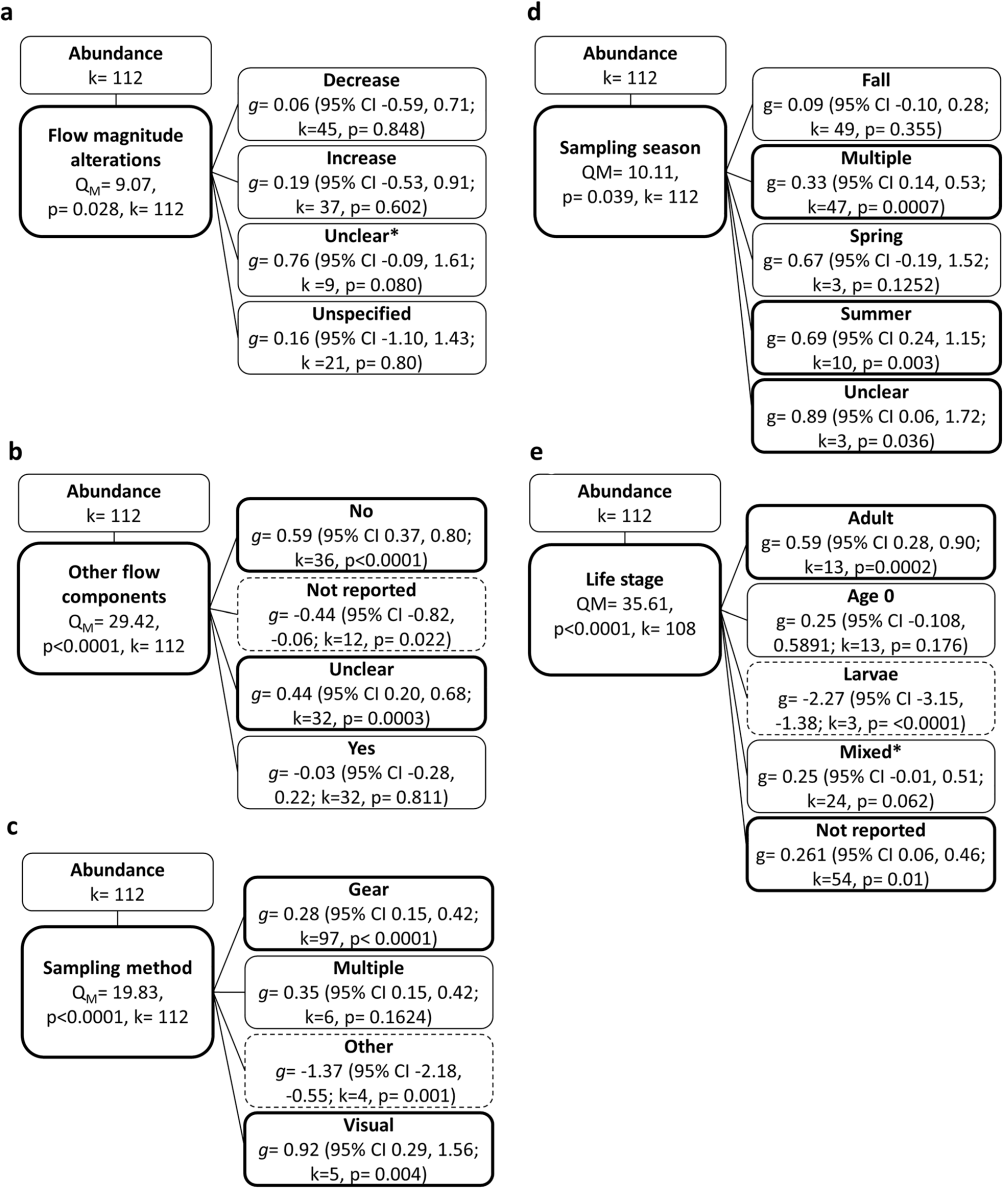
Summary flow chart of univariate mixed models and resulting significant moderators: (**a**) direction of flow magnitude alterations; (**b**) presence of alterations to other flow components; (**c**) sampling method; (**d**) sampling season; (**e**) life stage. *Indicates moderately significant effect (*p* < 0.1). Dashed boxes indicate statistically significant negative effects, thick solid line boxes indicate statistically significant positive effects (i.e., fish abundance is greater in the After period than the Before period). *k*: number of datasets (i.e., effect sizes); *g*: Hedges’ *g* mean effect size; CI: 95% confidence interval

To test for the influence of moderator variables on average fish responses to changes in flow magnitude, there were only sufficient sample sizes for interannual *BA* studies and fish abundance; there were too few effect sizes for abundance and biomass in the within-year *BA* studies and for biomass in the interannual *BA* studies to permit meaningful analyses. Note, for waterbody type and dam size, although there were sufficient sample sizes, there was insufficient variation to permit analyses (i.e., all 112 datasets occurred in river systems, at high head dams). For all analyses, we present the main results of univariate mixed models, and summarize all model results in Table 6 and significant model results in Fig. 12. Because of significant correlation among most moderators and small sample sizes, we were unable to combine multiple moderators into a single model (see results of Pearson’s ${\chi }^{2}$ test; Additional file 8: Table S2). However, the inclusion of these moderators left significant heterogeneity in all moderated models (Table 6), suggesting that interactions between moderators may be occurring, or other factors not captured by our analyses are influencing fish responses.

**Table 6:** Summary results of meta-analysis using subsets of fish abundance effect sizes for interannual Before/After studies, testing the influence of the given moderator variable

Moderator	k	*Q* statistic (*p*-value)	*Q*_*M*_ (*p*-value)	*Q*_*E*_ (*p*-value)
Waterbody type	112	N/A		
Dam size	112	N/A		
Hydropower operational regime				
Unmoderated model	112	**421.12 (*p* < 0.0001)**	–	–
Operational regime	112	–	1.35 (*p* = 0.717)	**412.00 (*p* < 0.0001)**
Direction of flow magnitude alteration				
Unmoderated model	112	**421.12 (*p* < 0.0001)**	–	–
Flow alteration	112	–	**9.07 (*p* = 0.028)**	**412.05 (*p* < 0.0001)**
Alterations to other flow components				
Unmoderated model	112	**421.12 (*p* < 0.0001)**	–	–
Other components	112	–	**29.42 (*p* < 0.0001)**	**391.70 (*p* < 0.0001)**
Sampling method				
Unmoderated model	112	**421.12 (*p* < 0.0001)**	–	–
Sampling technique	112	–	**19.83 (*p* = 0.0002)**	**401.29 (*p* < 0.0001)**
Sampling season				
Unmoderated model	112	**421.12 (*p* < 0.0001)**	–	–
Sampling season	112	–	**10.11 (*p* = 0.039)**	**411.01 (*p* < 0.0001)**
Type of comparator (temporal)				
Unmoderated model	112	**421.12 (*p* < 0.0001)**	–	–
Temporal comparison	112	–	1.63 (*p* = 0.203)	**420.92 (*p* < 0.0001)**
Time since intervention				
Unmoderated model	111	**420.78 (*p* < 0.0001**)	–	–
Time since intervention	111	–	1.96 (*p* = 0.376)	**418.83 (*p* < 0.0001)**
Monitoring duration (with outliers^†^)				
Unmoderated model	112	**421.12 (*p* < 0.0001)**	–	–
Monitoring duration	112	–	1.32 (*p* = 0.252)	**417.43 (*p* < 0.0001)**
Life stage				
Unmoderated model	108	**415.73 (*p* < 0.0001)**	–	–
Life stage	108	–	**35.36 (*p* < 0.0001)**	**380.12 (*p* < 0.0001)**

Bold indicates statistical significance (*p* < 0.05)

*k*: number of effect sizes; *Q* statistic: value of homogeneity test; *Q*_*M*_: omnibus test statistic of moderators; *Q*_*E*_: unexplained heterogeneity

Unmoderated model: random-effects model; *Significance at *p* < 0.1. ^†^Two extreme effect sizes were removed to improve model fit, but removal had little impact on results.

No detectable effects were found on average effect sizes from univariate mixed-effects models for the following moderators: (1) operational regime, (2) type of temporal comparator, (3) time since intervention (at < 1 year, one year, and two years after intervention), and (4) monitoring duration (Table 6).

The following five moderators were found to be associated with average effect sizes:

*Direction of flow magnitude alteration—*There were only sufficient sample sizes and variation to permit meaningful tests of the influence of grouped increases and decreases in flow magnitude, rather than increases and decreases in average discharge, peak flow, base flow and short-term variation separately (see Table 3 for definitions of flow magnitude intervention terms). The following levels within flow magnitude alterations were considered: (i) increase (combination of any increases in flow magnitude); (ii) decrease (combination of any decreases in flow magnitude); (iii) unclear (there was insufficient information to determine the overall increase/decrease in flow, but it was clear that flow magnitude had been altered); and (iv) unspecified (an alteration to flow magnitude was assumed but not explicitly stated). There was a statistically significant effect of the direction of flow magnitude on average fish abundance detected (Table 6), with studies including unclear alterations in flow associated with larger, positive effect sizes than those specifying decreases or increases in flow magnitude alterations (Fig. 12 and Additional file 12: Fig. S30); although average effect size for unclear alterations was only moderately significant and confidence intervals overlapped among all groups.

*Alterations to other flow components—*The presence of alterations to other flow components (i.e., frequency, duration, timing, rate of change, or surrogates of flow) was associated with average effect sizes (Table 6 and Fig. 12), although the response of fish abundance varied among interventions. Studies lacking alterations to other flow components had the largest, most positive average effect size (i.e., higher abundance in the After period than the Before period), whereas studies that did not report whether other flow components were altered had the most negative average effect size. Studies that did report the presence of alterations to other flow components had a slightly negative, but non-significant average effect size (Fig. 12 and Additional file 12: Figure S31).

*Sampling methods—*There were only sufficient sampling sizes and variation to include following levels within sampling methods: (i) gear + angling; (ii) visual; (iii) other (i.e., historical fishing data or hydroacoustics); and (iv) multiple (any combination or two or more methods). Sampling method was associated with average effect sizes, but the response of fish abundance to various methods varied (Fig. 12 and Table 6). Visual sampling methods were associated with the largest positive effect sizes, indicating that when using visual sampling methods average fish abundances were larger after the intervention than before. Similarly, gear + angling was associated with positive average effect sizes. ‘Other’ sampling methods was associated with negative average effect sizes, indicating the opposite response in abundance when those methods were utilized (Fig. 12 and Additional file 12: Fig. S32).

*Sampling seasons—*There were only sufficient sample sizes to allow inclusion of the following categorical levels: (i) Spring; (ii) Summer; (iii) Fall; (iv) Multiple (any combination of two or more seasons); and (v) Unclear (not reported + unclear). Sampling season was associated with average effect sizes (Table 6 and Fig. 12). The response of fish abundance to all seasons was positive (i.e., fish abundance was higher in the After period than in the Before period), with studies conducted in summer having the largest average effect size (other than studies that were not clear regarding which seasons were sampled). Studies conducted in multiple seasons were also associated with statistically significant average effect sizes (potentially because of the inclusion of summer samples within this group), although abundance of fish was lower than when only summer was considered (Fig. 12 and Additional file 12: Fig. S33).

*Life stage—*There were only sufficient sample sizes to consider the following life stages: (i) age-0; (ii) larvae; (iii) adult; (iv) mixed (any combination of life stages not reported separately); and (v) not reported (no specific life stage provided). Life stage was associated with average effect sizes (Table 6) with responses in fish abundance varying by life stage (Fig. 12). Adult fish were associated with larger, positive average effect sizes compared to mixed life stages (which had a moderate positive association with average effect size). Larvae were associated with the largest, most negative average effect size, indicating average fish abundance was lower for larvae after an intervention than prior to a change, but sample sizes were small (Fig. 12 and Additional file 12: Fig. S34).

#### Taxonomic analysis

Forest plots for all analyses are presented in Additional file 13.

##### Control/Impact studies

**Fig. 13:**
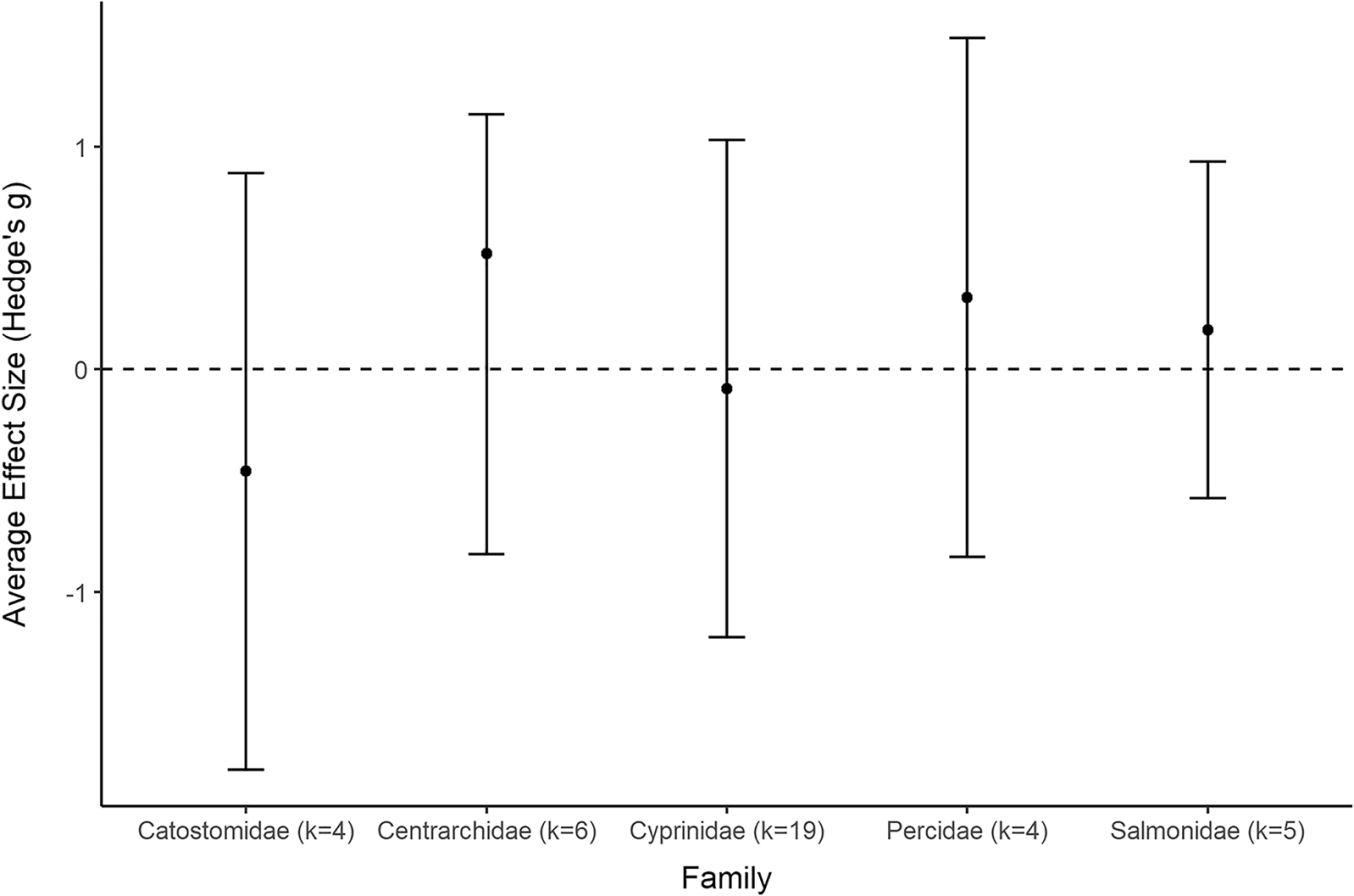
Average effect size by fish family for Control/Impact studies and abundance. Value in parentheses (*k*) is the number of effect sizes. Error bars indicate 95% confidence intervals. A positive mean value (above the dashed zero line) indicates that the abundance was higher in intervention than in comparator sites (no intervention). 95% confidence intervals that do not overlap with the dashed line indicate a significant effect (at the *p* < 0.05 level)

There were only sufficient sample sizes to investigate impacts of alterations to flow magnitude due to HPP facilities on abundance for five temperate freshwater fish families for *CI* studies: (i) Catostomidae, (ii) Centrarchidae, (iii) Cyprinidae, (iv) Percidae, and (v) Salmonidae. The families Catostomidae and Cyprinidae had overall negative responses, while Percidae, Centrarchidae and Salmonidae families had overall positive responses to flow magnitude alterations, although no family had a statistically significant overall response (Table 4B and Fig. 13). Based on the *Q* test of heterogeneity, there was significant heterogeneity among effect sizes for only the Cyprinidae family (*Q* = 39.41, *p* = 0.003) however there was insufficient variation in moderators to permit meaningful evaluation of the influence of moderator variables. Sample sizes were too small for moderator analysis of other families and abundance, or for analyzing biomass responses by taxa.

##### Within-year Before/After studies

There was only a sufficient sample size to investigate impacts of alterations to flow magnitude due to HPP facilities on abundance responses for one temperate freshwater fish family, Salmonidae, for within-year *BA* studies. Salmonidae had a moderately significant positive response to flow magnitude alterations [Hedge's *g* = 0.81 (95% CI − 0.15, 1.76; *k* = 4, *p* = 0.099)] (Table 4D). This may be due to a single statistically significant positive effect size related to *Oncorhynchus mykiss* in the Colorado River, where this species is an established non-native species that was previously stocked (Avery et al. 2015) (Additional file 13: Fig. S6). Based on the *Q* test of heterogeneity, heterogeneity within this family was not statistically significant. This may be due to the presence of only two species in this group of datasets: (i) *Oncorhynchus mykiss* (*k* = 3) and (ii) *Salmo trutta* (*k* = 1). Sample sizes were too small in this group to evaluate influences of moderator variables within the abundance outcome for this family. No within-year *BA* studies considered biomass outcomes.

##### Interannual Before/After studies — Abundance

There were sufficient sample sizes to investigate impacts of alterations to flow magnitude due to HPP facilities on abundance for nine temperate freshwater fish families for interannual *BA* studies: (i) Acipenseridae; (ii) Anguillidae; (iii) Catostomidae; (iv) Centrarchidae; (v) Cottidae; (vi) Cyprinidae; (vii) Esocidae; (viii) Ictaluridae; and (ix) Salmonidae. For families with significant heterogeneity among effect sizes (i.e., significant *Q*), additional analyses were performed for genera therein with sufficient sample size (i.e., ≥ 3 datasets from ≥ 2 independent studies) (Additional file 13: Fig. S17–S22).

**Fig. 14:**
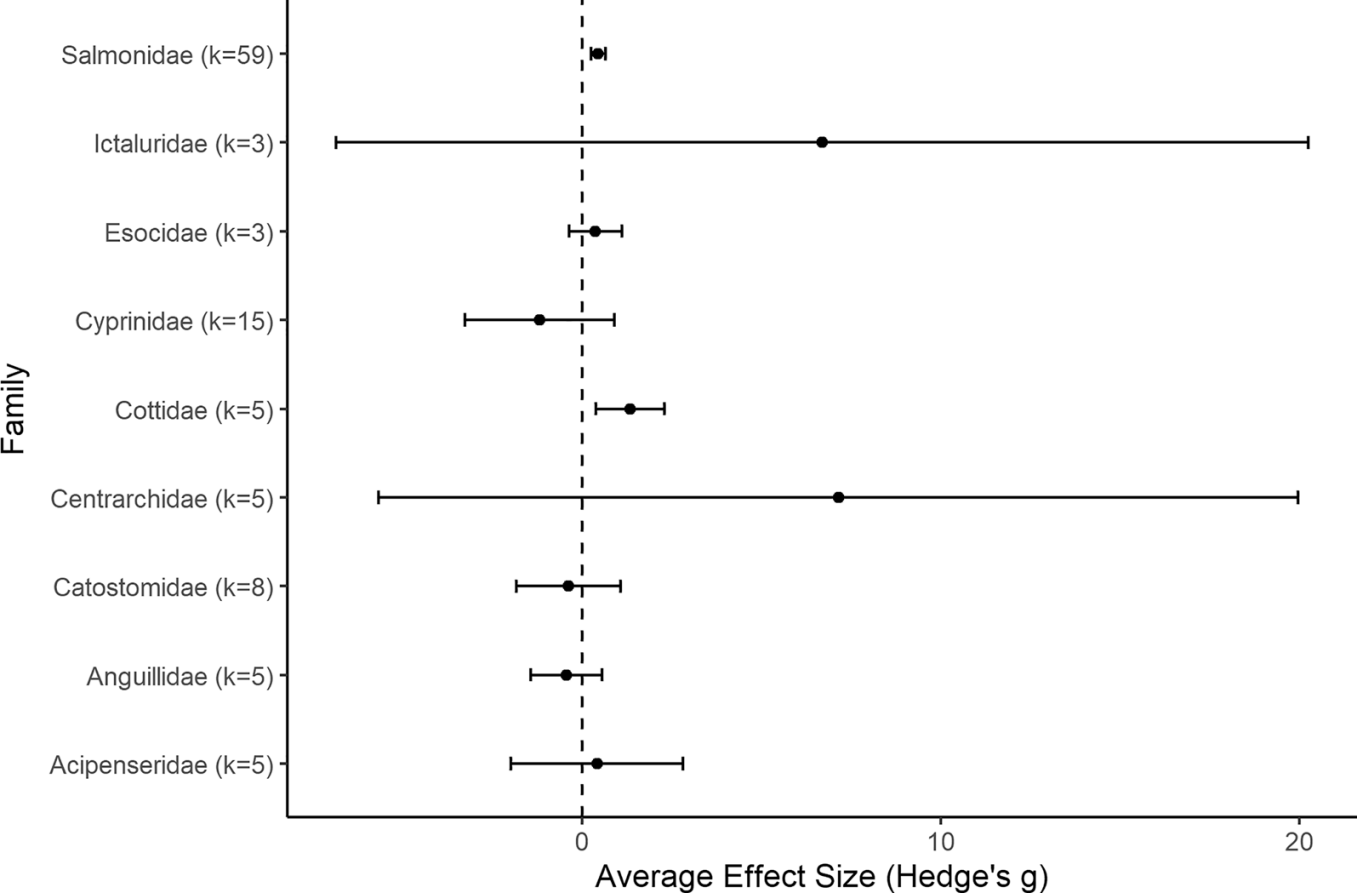
Average effect size by fish family for interannual Before/After studies and abundance. Value in parentheses (*k*) is the number of effect sizes. Error bars indicate 95% confidence intervals. A positive mean value (right of the dashed zero line) indicates that the abundance was higher in the After period (intervention) than in the Before period (no intervention). 95% confidence intervals that do not overlap with the dashed line indicate a significant effect (at the *p* < 0.05 level)

The families Acipenseridae, Centrarchidae, Esocidae and Ictaluridae had overall positive but nonsignificant responses to alterations in flow magnitude (i.e., fish abundance was greater after an intervention than prior to the intervention) (Table 4F and Fig. 14). Centrarchidae and Ictaluridae had strong positive responses to flow alterations, but the heterogeneity for both was significant and much larger than for the other families considered (Centrarchidae: *Q* = 29.06, *p* < 0.0001; Ictaluridae: *Q* = 30.34; *p* < 0.0001) (Fig. 14). Anguillidae, Catostomidae and Cyprinidae all had negative overall mean effect sizes, but also had nonsignificant responses to alterations in flow magnitude (Table 4F and Fig. 14). In contrast, alterations to flow magnitude were estimated to have overall positive and significant effects on salmonid and cottid abundance. Based on the *Q* test of heterogeneity, there was also significant heterogeneity among effect sizes for Acipenseridae, Catostomidae, Cyprinidae, and Salmonidae. Although heterogeneity was present within these families, we only conducted analyses at the genera level when more than one genus with sufficient sample sizes were present (i.e., Cyprinidae and Salmonidae; see Additional file 13 section "Interannual Before/After: Genera"). Anguillidae, Cottidae and Esocidae did not have statistically significant heterogeneity (Additional file 13).

##### Interannual Before/After studies—Biomass

There was only sufficient sample size to investigate impacts of alterations to flow magnitude due to HPP facilities on biomass responses for one temperate freshwater fish family, Salmonidae, for interannual *BA* studies. The overall weighted effect size for Salmonidae indicated that alterations in flow magnitude may have a positive effect on fish biomass, but that the response was not significant (Table 4F). There was no statistically significant heterogeneity among effect sizes (*Q* = 15.02; *p* = 0.131). There was sufficient sample size to investigate one genus in this family (*Oncorhynchus*; Additional file 13: Fig. S23).

## Review limitations

Our analysis of flow magnitude alteration impacts due to hydropower production and operations on fish abundance and biomass did not find consistent patterns in fish responses to increases or decreases in flow magnitude. This review’s ability to evaluate fish responses was limited by both the study validity (i.e., high susceptibility to bias) and quantity of available evidence. Although our approach targeted a single aspect of the fish-flow relationship, like past reviews (e.g., Poff and Zimmerman [20]; Webb et al. [21]; Young et al. [64]; McManamay et al. [65]) we found that fish responses to alterations in flow magnitude were highly variable (Table 4).

### Limitations of review methods

We attempted to minimize potential biases in our review methodology throughout the systematic review process. Our diverse advisory team and outreach to experts and practitioners helped us identify as many relevant and reliable studies as possible and decreased familiarity bias. While we identified 133 relevant studies, 58 of which were eligible for quantitative analysis, we acknowledge that our review does not represent the entirety of the knowledge base on the subject. Efforts were made to obtain all relevant materials to decrease availability bias; however, several reports and publications (n = 17) were unobtainable (Additional file 3). Our review is limited to only English articles. Although this captures most articles available and relevant to the North American and Canadian context, we acknowledge that Canadian reports on hydroelectric power production published in French were likely missed. Efforts were made to capture these reports when sufficient data were available in English summaries (4 articles). There may also be additional, valuable articles and grey literature from other temperate-region countries not published in English. We did not use non-English search terms in the systematic map [33] or this review, but there were relatively few articles excluded from the map on language at full text (61/2412 articles) and only four excluded on language during this review (Fig. 1). This low number suggests there may be only a slight risk of language bias.

There was no apparent evidence of publication bias for fish biomass (Additional file 12: Fig S7 and S25); however, sample sizes were small. There was possible evidence of publication bias for abundance in both *CI* and interannual *BA* studies towards studies showing increased abundance in the intervention site relative to controls (Additional file 12: Fig. S2 and S17). When separating publication bias by publication type, evidence of publication bias towards positive results was present in grey literature, but not in commercially published literature. A possible explanation may be that these reports are commissioned by hydropower operators to quantify impacts of flow alterations at their facilities, which may have led to lower reporting of negative results, due to the types of questions being investigated (e.g., practitioners focus on flow improvements), whereas commercially published literature may focus on the overall impacts of flow alterations. It is almost certain that additional grey literature exists (especially for earlier decades where grey literature made up less than 50% of studies identified; Fig. 3) in internal documents that were not accessible to our review team.

### Limitations of the evidence base

Of the 133 studies included in this review, 75 were excluded from quantitative synthesis largely for (i) qualitative outcomes in the intervention and/or comparator groups (e.g., presence/absence) or (ii) single data points in the intervention and/or comparator groups (i.e., it was not possible to calculate an average due to a lack of replication). Both cases meant that an effect size could not be calculated. Similarly, most studies in this review did not report within-year fish responses (i.e., data for each month or season of sampling reported separately for each sampling year), instead providing single data points, sums or averages across within-year sampling without also providing within-year fish outcomes over several years. As a result of this finer-scale, within-year sampling data not being reported separately, we were unable to analyze variability through time effectively. Reporting quantitative fish outcomes and providing raw or finer-scale sampling data, rather than pooling data across samples, would improve future (systematic) reviews. With the prevalence and availability of online data repositories, authors should continue to be encouraged to provide raw data.

Our inability to clearly identify relationships between flow and fish abundance and biomass may, in part, be due to our inability to quantify the amount of flow alteration experienced (i.e., ΔQ, where Q is discharge). However, we felt that our qualitative descriptions of flow alteration based on author descriptions allowed us to capture a greater percentage of studies for quantitative analysis than would otherwise have been possible. Effectively quantifying differences in flow magnitude was complicated by inconsistent reporting of flow alterations among studies. Several studies quantified flow magnitude or included hydrographs for intervention groups without also including measures of flow for comparator groups. In other instances, historical hydrographs were included, but only qualitative descriptions of the specific flow alteration being investigated were reported. This made possible calculations of the amount of flow alteration unfeasible. We recommend that when reporting alterations in flow magnitude, comparable data (i.e., measured flow magnitude or hydrographs) from the same temporal period (i.e., season) be included for both the intervention and comparator groups. Expanding and improving flow monitoring systems throughout impacted and unimpacted waterbodies would assist in these efforts.

A challenge in assessing flow alterations and fish responses is knowing if the response seen in fish outcomes can actually be attributed to the alteration in flow [66]. Collectively, the studies reviewed here did not provide clear insight into the impact the direction of flow magnitude alterations would have on fish and whether the apparent increases or decreases were due to flow alterations, or some other factor(s). The relatively low validity of included studies may have influenced this result. Of the datasets included for quantitative analysis, 51% had ‘Medium’ validity, while the remainder had ‘Low’ validity. When ‘Medium’ validity studies are considered alone, the relative magnitude of the effect size for abundance in *CI* studies is larger when compared to the overall model (i.e., an increase from *g* = − 0.001 to *g* = − 0.18; Additional file 12: Table S1). The inclusion of ‘Low’ validity studies leads to smaller effect sizes, although overall effect sizes were non-significant in either case. Improving study design by including temporal and spatial replication, improving comparator matching and improving reporting of flow alterations would all aid in improving internal validity of primary studies and, subsequently, the reliability of future evidence syntheses.

To properly assess the impacts of flow alterations, including both spatial and temporal replication within the same study is essential (i.e., *BACI* designs). If studies focus on spatial replication, changes in responses through time may be missed, while studies focused on temporal replication may miss underlying spatial variability or change. *BACI* studies were less represented in our quantitative synthesis than other study designs (13 cases; see “Study design and comparator”), likely due to the time and complexities required in these study designs. This limited our ability to examine these studies specifically, or at the interaction level, because they had to be converted for inclusion in analysis. In simulation, *BACI* designs outperform other study designs, including *BA* and *CI* designs, with higher accuracy and less bias [67]. In an ecological context *BACI* designs have benefits over *BA* or *CI* designs because they help decrease the likelihood of erroneous conclusions based on the inherent assumptions of similarity of spatial sites or Before/After periods in these designs [68]. We had opposing overall effect sizes when considering studies with different replication (i.e., negative and positive effect sizes for *CI* and *BA* studies, respectively). This may be a function of *BA* study designs potentially providing more accurate results than *CI* designs [67] and indicates that for a more complete picture, including both spatial and temporal replication would be helpful to truly understand outcomes.

Many articles were excluded due to choice of comparator or were assessed to have low study validity during critical appraisal because of imperfect matching. During screening, 144 articles were excluded due lack of usable comparators or any comparator (i.e., spatial or temporal trends). While we acknowledge that many of these studies asked different questions than this review, in cases where similar questions were asked, and only trends were considered, important aspects of flow and fish dynamics were likely missed. For instance, basin-scale factors or declines in fish abundance along the entirety of a river may be wrongly attributed to a flow alteration if no comparator group is included. Several studies were excluded due to the use of downstream comparators. Although upstream impacts attenuate downstream [66], it is unlikely that downstream sites will ever truly be unimpacted. Indeed, impacts of upstream dams can be detectable hundreds of kilometers downstream [69]. We recommend that researchers limit the use of downstream comparators when studying flow alterations and instead, use upstream or unimpacted comparators, although they present their own challenges (e.g., site matching or availability).

Most Control/Impact studies included in this review lacked true spatial replicates and all *BA* studies lacked replication across waterbodies. Pseudoreplication was more common than true replication in the surveyed literature, but considering only studies with true replication led to a larger, overall negative effect size, indicating that including pseudoreplicated studies may result in a smaller overall effect size (Additional file 12: Table S1). Although the inclusion of pseudoreplication may lead to issues of nonindependence of samples [70], true replication may not be possible in many situations where identifying similar sized dams, operations and hydrological settings, and sampling from these true replicates during similar periods of dam operation, is difficult [66]. Efforts should be made to at least sample in multiple locations downstream and upstream of an intervention, and to control for pseudoreplication during analysis [71]. Caution is warranted when selecting upstream comparators, as dams may act as barriers to dispersal and movement [72], and any apparent increase in fish abundance downstream may be due to pooling below the dam and loss of fish upstream, rather than an actual increase in population. In systems where multiple dams or dam cascades are present and impacts may interact, ensuring spatial comparators are outside the influence of any dam can be extremely difficult, but not impossible (e.g., Bowen et al. [73]). Spatial replication should still be attempted and the potential impacts of upstream facilities should be explicitly stated in any study within these systems.

We were unable to draw clear conclusions of time lags or long-term effects of alterations in flow magnitude on fish abundance or biomass. This was because many *CI* and interannual *BA* studies included in quantitative synthesis were based on short-term monitoring. We found that summary effect sizes for individual post-intervention monitoring years were no longer significant (p < 0.1) after two years of monitoring changes in flow magnitude (Fig. 11); however, this finding may be due to a decrease of available information over time (i.e., summary effect size estimates beyond one year of post-intervention monitoring were all based on sample sizes of ≤ 5). Long-term studies are important to identify changes in responses through time and help elucidate patterns or factors that are otherwise missing in short-term data [74, 75]. For example, if fish respond differently after several years of exposure to an intervention, short-term studies may not capture these changes. This is especially true if a single life stage or sampling period is considered [74]. Population decreases may not be immediately apparent in long-lived species if only adults are considered for short, 1–2 year periods; conversely, potential benefits of flow alterations may increase in value over time as fish adapt to new flow regimes. Of studies included for quantitative analysis, one reported > 4 years [76] and could be used to assess time lags, and only two *CI* studies reported ≥ 3 years. Interannual *BA* studies were often longer in duration, with six studies lasting a decade or more. These types of studies should be encouraged. When paired with flow experiments, these types of studies can expose aspects of responses that would otherwise be obscured, or even open new avenues of research [77].

A potential seasonal bias was present in our quantitative synthesis. Many studies (30/58) considered a single sampling season (corresponding to 117/256 datasets). Because fish populations change with seasons, focusing on a single sampling period may overemphasize responses that are due to population behaviour or other seasonally influenced environmental factors [78]. Several studies also used comparators sampled in different seasons for the same species (5 studies, 19 datasets). This may lead to additional issues interpreting responses if a species goes through episodic population fluctuations or variable seasonal reproduction [79]. Long term, multi-seasonal studies could help alleviate these risks, while studies conducted in a consistent season over a longer time period can provide useful insight into general population changes (e.g., pink salmon; [80]). Winter fish sampling was underrepresented in both the narrative (11% of cases) and quantitative synthesis (11% of datasets) and was rarely reported individually to isolate fish responses during this season. There is a general lack of knowledge on the importance of winter in fish population dynamics [81] and fish responses to flow alterations during this period were comparatively missing in our database. The lack of sampling during this period, potentially due to logistical, safety, and methodological challenges associated with sampling fish during winter [82, 83], may bias apparent fish responses to flow alterations and ultimately limit our ability to fully understand species responses to complex flow regimes.

Geographical and taxonomic biases were evident in the quantitative synthesis. Datasets were primarily from North America (71%) of which 58% were from the United States. Although 98 species were represented within the quantitative synthesis, only six species had more than eight datasets, five of which were from the Salmonidae family (30%). Similar geographic and taxonomic biases were also identified in the systematic map [33] and have been identified in other hydropower related reviews for temperate regions (e.g., Algera et al. [27]). This likely limits the applicability of our review results for other geographic regions and taxa.

### Reasons for heterogeneity

Overall mean effect sizes ranged from positive to negative and varied depending on outcome (abundance or biomass), study design (*CI*, within-year *BA* or interannual *BA*) and taxa considered (Table 4). For *CI* studies, alterations in flow magnitude led to effect sizes that indicated almost no change or negative changes in abundance, while fish biomass was estimated to have a negative overall effect size (i.e., was lower relative to comparators not receiving an intervention). In contrast, for *BA* study designs, both abundance and biomass had generally higher values in the After period relative to the Before period for within-year and interannual *BA* study designs (i.e., a positive overall response to flow magnitude alterations). This difference may arise because *CI* study designs compared fish outcomes at an impacted site to a non-impacted comparator (i.e., upstream of the HPP facility or a different unimpacted waterbody), whereas many *BA* studies reported alterations to flow at existing HPP facilities that were made specifically to provide potential benefits to fish (i.e., increases in base flow). None of the overall effect sizes from *CI* or interannual *BA* studies were statistically significant (*p* < 0.05), but abundance in After year-1 and After year-2 of within-year *BA* studies did have moderately significant effect sizes, although sample sizes were small (Table 4). These results are consistent with past reviews, which also found that fish responses to flow magnitude alterations were highly variable and context dependent [21, 29, 65]. It has been argued that over-generalization or simplified ‘rules-of-thumb’ applied across systems should be avoided [84] and, because river systems have unique physical properties [66, 85] and communities [86, 87], knowledge of one system cannot necessarily be transferred to other systems. Our results provide additional support for this.

Taxonomic responses varied across families, although interestingly, responses of specific taxa were consistent across *CI* and interannual *BA* studies (Table 4). Overall mean effect sizes for Catostomidae indicated a decrease in abundance relative to a comparator in both *CI* and *BA* study designs, as did those for Cyprinidae, while effect sizes for both Centrarchidae and Salmonidae saw overall increases relative to comparators (Table 4). Furthermore, a strong positive response to changes in flow magnitude was estimated for cottid abundance from *BA* studies, and there was little heterogeneity in effects sizes, suggesting a consistent response from this family that was represented by a single genus (*Cottus*) (Fig. 14; Additional file 13). The overall responses of these families may indicate that, although a generalizable trend across taxa may not be possible, specific families, genera or species may respond consistently to changes in flow magnitude. Caution should be taken when interpreting this result for most taxonomic groups, however, because we were unable to explore potential moderators and sample sizes were small.

Several moderators were tested in our quantitative synthesis to explore reasons for heterogeneity among responses. Moderator analysis for *CI* studies was inconclusive, with no detectable effect of any moderators concerned with dam operations (i.e., dam size, hydropower operational regime, direction of flow magnitude change), potential confounders (i.e., alterations to other flow components, time since sampling) or study design/methods (i.e., sampling season or method, type of comparators used, or monitoring duration). In contrast, several moderators were associated with the overall effect sizes for abundance and interannual *BA*s including: (i) direction of flow magnitude alterations (i.e., studies including unclear alterations in flow were associated with larger, positive effect sizes than those specifying decreases or increases in flow magnitude alterations); (ii) presence of alterations to other flow components (i.e., studies lacking alterations to other flow components had the largest, most positive average effect size compared to studies that did not report whether other flow components were altered, which had the most negative average effect size); (iii) sampling method (i.e., visual and gear + angling sampling methods were associated with positive effect sizes, indicating that when using these sampling methods average fish abundances were larger after the intervention than before); (iv) sampling season [i.e., fish abundance was higher in the After period than in the Before period for all seasons (positive responses to changes in flow magnitude), with studies conducted in summer having the largest average effect size]; and (v) life stage (i.e., adult fish were associated with larger, positive average effect sizes compared to mixed life stages, which had a moderate positive association with average effect size, and larvae were associated with the largest, most negative average effect size) (Additional file 12: S30–34). However, considerable residual heterogeneity remained in the observed effects of hydropower production, suggesting that interactions between moderators may be occurring or that some other factor(s), not captured in our analysis, may be influencing fish abundance. Most moderators were highly correlated (see Pearson’s χ^2^ test Additional file 8: Tables S1 and S2), complicating interpretation and making models with multiple variables impracticable due to small sample sizes [88]. It was, therefore, difficult to determine the impact of each moderator on overall mean effect sizes.

## Review conclusions

### Implications for policy/management

Systematic reviews with meta-analysis aim to generalize ecological relationships and explore differences in individual study characteristics and heterogeneity in results [34]. In some instances, results of systematic reviews may be ambiguous [89] and generalizations may not be possible. Previous reviews on fish/flow relationships determined that generalizable and transferable relationships between flow components (such as magnitude, frequency, duration, timing and rate of change) and species responses were not possible with the state of the literature base [20] and that relationships were highly context dependent [65]. Nearly a decade later, and with a more extensive, targeted review considering a single flow component, the results of our review are consistent with these findings. Generalizable signals were very difficult to identify, and generalization may not be possible in systems impacted by hydropower facilities where the specific features of the system (i.e., size, underlying hydrology, community dynamics) are highly influential. Our analyses provide some evidence that changes or alterations in flow magnitude may lead to positive responses in fish abundance overall (i.e., based on within-year Before/After study designs, one and two years after a change in flow magnitude has occurred), and for particular fish taxa (i.e., Cottidae: *Cottus*; Salmonidae: *Oncorhynchus* and *Salmo*). However, sample sizes were small, effectively hampering our ability to determine whether these outcomes were influenced by the direction of the flow magnitude alteration (i.e., due to an increase or decrease in flow magnitude). Overall, our results imply that fish responses to flow magnitude alterations or changes are likely context dependent, and as such, water resource and fisheries managers may need to take a site-specific and adaptive management approach. To this end, this systematic review provides managers with a comprehensive evidence base that they can use to assess the evidence available in the literature that is relevant to their specific contexts and/or regions (e.g., particular freshwater systems, HPP operating regimes, species or species groups).

### Implications for research

Other fish responses may be occurring that are not apparent in the outcomes examined here. If individual fish species or taxa respond differently to flow alterations, as seen in our taxonomic analyses for *CI* and *BA* studies, compositional changes in fish populations may result. Changes in composition have been seen in impoundments [29] and assemblage dominance and species composition has been found to differ between sites upstream and downstream of HPP facilities, or after flow alterations [22, 90]. Recent estimates of biodiversity changes in freshwater systems due to human-induced alterations indicate that temperate regions have experienced among the largest biodiversity changes of any region [91]. How different populations are responding to hydropower production and operations in terms of compositional changes is an area for further exploration.

Drawing from issues we encountered during quantitative analysis and common features of studies in our evidence base, some recommendations for improving future study designs and reporting are possible. To better assess the impacts of flow magnitude alterations due to hydropower operations on fish abundance and biomass through future systematic review and quantitative synthesis, we make the following recommendations for primary studies (see Box 1).

To compensate for the lack of generalizable signals identified during this review, regional, long term, continuous monitoring to inform decision making will help improve clarity. Adaptive management and long-term manipulative flow studies can further aid decision makers in learning more about their specific systems [77] and in developing flows that provide for both energy and ecological needs [24]. Work should continue to grow the evidence base of fish/flow relationships, but should focus on long-term, high-quality site- and species-specific efforts to improve our understanding of how specific species in specific locations interact with flow. Our inability to identify generalizable trends, even with our comprehensive approach, lends credence to the need for sustained, high quality regional science for supporting management decisions in systems impacted by flow magnitude alterations due to hydropower production and facility operation. Although it would be desirable to identify general science-based ecological rules and relationships that extend across regions and taxa, it is evident that such goals remain elusive in the context of fish-hydropower interactions.

Box 1. Ways to improve study design and reporting for studies of the impacts of flow magnitude alterations on fish abundance and biomass *Controls—*Authors should make every effort to incorporate temporal and/or spatial comparators in their studies to ensure adequate baselines and improve understanding of impacts. Although difficult, resource intensive, and demanding of advanced planning, full *BACI* study designs are essential to properly account for temporal and spatial confounders. If not possible, selecting more accurate study designs [e.g., *BA* are considered more accurate than *CI* studies] with comparators that are carefully matched will facilitate more accurate quantitative synthesis results. Care should be taken to avoid downstream comparators whenever possible and to minimize gaps between temporal sampling periods (i.e., Before and After sampling periods)

*Duration—*When designing studies to assess fish responses to flow magnitude alterations, long-term monitoring (i.e., > 2 years) both prior to and post-intervention would facilitate improved understanding of population level effects and time-lags in responses. This is especially important for longer-lived species. Efforts should be made to minimize gaps between sampling years, and to ensure sampling occurs in multiple seasons

*Replication*—Care should be taken to ensure that appropriate levels of replication are included. Authors should ensure replication occurs in both the intervention and comparator, to facilitate inclusion of more studies in quantitative synthesis. When combining studies in syntheses, more accurate results (i.e., those from true replication) are preferable to more precise results (i.e., those from pseudoreplication). However, as true replication is not always possible, authors who find themselves in situations where true replication is unobtainable should still aim to include replicate sampling (even if pseudoreplicates)

*Reporting*—Studies should report sufficient detail regarding location of sample sites (i.e., latitude and longitude) and clear, detailed descriptions of sampling design and justifications for design choices. When possible, studies should report summarized data separately for monthly or seasonal samples within a year, and report detailed descriptions of how samples are grouped for analysis or provide raw data. Authors should make every effort to include a detailed description of all hydropower facility design and operations as well as both qualitative and quantitative descriptions of flow regime alterations. Studies must also adequately describe the pre-intervention and/or comparator site as they do the post-intervention and/or intervention sites, to capture vital information on confounding factors or differences in starting conditions. Where information cannot fit within published articles, details should be included in supplementary materials

## Supplementary Information

**Additional file 1.** ROSES systematic review report.

**Additional file 2.** Search strategy and results. Provides a description of the search strategy and results of the literature searches. For each source, we provided full details on the search date(s), search strings used, search settings and restrictions, and subscriptions (if applicable), and the number of returns.

**Additional file 3.** Excluded articles. List of articles excluded on the basis of full-text assessment or data extraction and reasons for exclusion. Separate lists of articles excluded on the basis of full-text assessment, assessment during data-extraction, articles that were unobtainable and relevant reviews.

**Additional file 4.** Study validity assessment description. Provides further description of the study validity assessments, along with the critical appraisal tool.

**Additional file 5.** Data-extraction sheet. Contains the coding (extracted data) for all articles/studies included in the narrative synthesis. Includes a description of the coding form, the actual coding of all articles/studies, and a list of supplementary articles.

**Additional file 6.** Data extraction considerations. Provides a description of further data extraction considerations, including those for *BA* study designs.

**Additional file 7.** Data preparation and additional calculations for quantitative synthesis. Provides a description of data preparation for quantitative synthesis in relation to converting *BACI* studies into *CI* or *BA* designs, reducing multiple effect size estimates from the same study, and our handling of pseudoreplication.

**Additional file 8.** Correlation analyses of moderators (Pearson’s *χ*^2^) and outlier investigations. Contains results of contingency analysis for independence of moderators, and mixed-effects model comparison when including monitoring duration with and without two extreme outliers.

**Additional file 9.** Study validity assessment results. Contains results of assessments for each article/study included in the narrative synthesis.

**Additional file 10.** Additional data descriptions for narrative and quantitative syntheses. Contains further descriptions of data for narrative synthesis (including details on study location, fish species list, and interventions) and quantitative synthesis (including details on interventions and outcomes).

**Additional file 11.** Quantitative synthesis database. Contains the coding (extracted data) for all articles/studies/datasets included in the quantitative synthesis. Includes the actual coding of all articles/studies/datasets, the calculations of effect sizes, calculations for aggregating effect sizes and a list of articles/studies/datasets not considered during quantitative meta-analysis.

**Additional file 12.** Meta-analyses and publication bias. Global meta-analyses, publication bias, sensitivity analyses, and moderator analysis. All forest (i.e., summary plot of all effect size estimates) and funnel (i.e., visual assessment of publication bias using a scatter plot of effect sizes versus a measure of precision) plots from global and sensitivity analyses.

**Additional file 13.** Taxonomic analysis. Includes forest plots for all families with sufficient sample sizes and for, families with significant heterogeneity, genera therein with sufficient sample size for further analysis (i.e., ≥ 3 datasets from ≥ 2 independent studies.

## Data Availability

All data generated or analysed during this study are included in this published article and additional files. A ROSES form [35] for this systematic review report is included as Additional file 1.
