# Supplementary material for: How do changes in flow magnitude due to hydropower operations affect fish abundance and biomass in temperate regions? A systematic review
Source: Environ Evid. 2022 Feb 4;11:3. doi: 10.1186/s13750-021-00254-8 (PMC8813579; doi:10.1186/s13750-021-00254-8)
Supplement: Supplementary file 2 — Additional file 2. Search strategy and results. Provides a description of the search strategy and results of the literature searches. For each source, we provided full details on the search date(s), search strings used, search settings and restrictions, and subscriptions (if applicable), and the number of returns. [file 13750_2021_254_MOESM2_ESM.docx]

**Additional File 2. Search strategy and results**

Description: This Additional File provides a description of the search strategy and results of the updated literature searches (2017-2019). For each source, we provided full details on the search date(s), search strings used, search settings and restrictions, and subscriptions (if applicable), and the number of returns. For details and results of the search strategy used for the recent systematic map (i.e., literature prior to 2017), refer to Rytwinski et al. (2020); note, all potentially relevant studies identified by the systematic map were included for this review at the data extraction stage and then screened on the specific eligibility criteria of this review.

**Databases**

The search string was developed based on suggestions from the Advisory Team as described in the protocol of the review (Harper et al. 2020).

The following bibliographic databases were searched from October - December 2019 using Carleton University’s institutional subscription

1. ISI Web of Science Core Collection — multidisciplinary research topics including journals, books, proceedings, published data sets and patents
2. ProQuest Dissertation & Theses Global — collection of dissertations and theses from around the world, spanning from 1743 to present
3. Scopus — abstract and citation database of peer-reviewed literature including journals, books, and conference proceedings.
4. Federal Science Library (Canada) — Canadian government books, reports, government documents, theses, conference proceedings, and journal titles
5. Science.gov — US Federal Science
6. AGRICOLA (Agricultural Research Database) — US Department of Agriculture’s National Agricultural Library

***Web of Science: Search Strategy #1***

Note: “Topic” search in Web of Science includes: title, abstract, keywords, keywords plus

Table S1. Metadata from Web of Science Search Strategy #1

| **Search string** | **Restrictions** | **Returns [Date]** |
| --- | --- | --- |
| TS=((Fish*) AND (“Fresh water” OR Freshwater OR Stream$ OR Water$ OR River$ OR Fluvial OR Estuar* OR Reservoir$ OR Impoundment$ OR "Hydro electric*" OR Hydroelectric* OR "Hydro dam*" OR Hydrodam* OR "Hydro power" OR Hydropower OR "Hydro" OR Dam$) AND (Flow* OR Discharg*) AND (Productivity OR Biomass OR Abundance$ OR Densit* OR Yield$ OR “Ecological response” OR “Ecosystem response” OR “Biotic response”) NOT (mining OR "mine site" OR aquaculture OR "wastewater treatment" OR carbon)) | - 2017-2019 - Web of Science Core Collection - Advanced search - Topic field - All languages - All document types - Institution subscriptions:   - Science Citation Index Expanded (1900 - present)   - Social Sciences Citation Index (1956 - present)   - Arts & Humanities Citation Index (1975 - present)   - Conference Proceedings Citation Index - Science (1990 - present)   - Conference Proceedings Citation Index - Social Science and Humanities (1990 - present)   - Book Citation Index - Science & Social Science (2008 - present)   - Current Chemical Reactions (2008 - present)   - Index Chemicus (2008 - present)   - Part of the larger Web of Science. | **818**  [December 1, 2019] |

***ProQuest Dissertations & Theses Global: Search Strategy #2***

Note: Command line advanced search selected to search within: title, abstract, keywords.

Table S2. Metadata from ProQuest Dissertations & Theses Global Search Strategy #2

| Search string | Restrictions | Returns [Date] |
| --- | --- | --- |
| TI,AB,IF(Fish* AND (“Fresh water” OR Freshwater OR Stream OR Water OR River OR Fluvial OR Estuar* OR Reservoir OR Impoundment OR "Hydro electric*" OR Hydroelectric* OR "Hydro dam*" OR Hydrodam* OR "Hydro power" OR Hydropower OR "Hydro" OR Dam) AND (Flow* OR Discharg*) AND (Productivity OR Biomass OR Abundance OR Densit* OR Yield OR “Ecological response” OR “Ecosystem response” OR “Biotic response”) NOT (mining OR "mine site" OR aquaculture OR "wastewater treatment" OR carbon)) | - Specific Date Range (2017 - 2019) - Dissertations & Theses Global - Master’s and doctoral dissertation - All languages - English only search terms - Institutional subscription   - Indexing 1743-present; Full text 1997-present   - PQDT Global includes theses from Great Britain and Ireland | **76**  [December 1, 2019] |

***Scopus: Search Strategy #3***

Note: Advanced search selected to search within: title, abstract, and keywords.

Table S3. Metadata from Scopus Search Strategy #3

| Search string | Restrictions | Returns [Date] |
| --- | --- | --- |
| TITLE-ABS-KEY (Fish*) AND TITLE-ABS-KEY(“Fresh water” OR Freshwater OR Stream OR Water OR River OR Fluvial OR Estuar* OR Reservoir OR Impoundment OR "Hydro electric*" OR Hydroelectric* OR "Hydro dam*" OR Hydrodam* OR "Hydro power" OR Hydropower OR "Hydro" OR Dam) AND TITLE-ABS-KEY(Flow* OR Discharg*) AND TITLE-ABS-KEY(Productivity OR Biomass OR Abundance OR Densit* OR Yield OR “Ecological response” OR “Ecosystem response” OR “Biotic response”) AND NOT TITLE-ABS-KEY(mining OR "mine site" OR aquaculture OR "wastewater treatment" OR carbon) | - 2017-2020 - Advanced search - All subject areas - All languages - All documents types - English only search terms | **774**  [December 1, 2019] |

***Federal Science Library: Search Strategy #4***

Available online: <https://fsl-bsf.summon.serialssolutions.com/en/advanced#!/advanced?l=en>; Federal Science Library (Formerly WAVES).

Note: Advanced search using Subject field searched words in the title, subject, series and abstract areas of the Federal Science Library record. Searching is a little more limited than previous databases.

Table S4. Metadata from Federal Science Library Search Strategy #4

| Search string | Restrictions | Returns [Date] |
| --- | --- | --- |
| (subjectTerms:(Fish*)) AND (subjectTerms:(Flow* OR Discharg*)) AND (subjectTerms:(Productivity OR Biomass OR Abundance OR Densit* OR Yield OR “Ecological response” OR “Ecosystem response” OR “Biotic response”) AND (subjectTerms:( (“Fresh water” OR Freshwater OR Stream OR Water OR River OR Fluvial OR Estuar* OR Reservoir OR Impoundment OR "Hydro electric" OR Hydroelectric* OR "Hydro dam" OR Hydrodam* OR "Hydro power" OR Hydropower OR "Hydro" OR Dam)) NOT (subjectTerms:(mining OR "mine site" OR aquaculture OR "wastewater treatment" OR carbon)) | - 2017 - 2019 - Advanced search - No language specified - Any content types - Any disciplines - No limits, no exclusions - Not expanded to outside your library’s collection - Sort by Relevance - Open access – no institutional | **82**  [December 1, 2019] |

***Science.gov: Search Strategy #5***

Available online: <https://www.science.gov/scigov/desktop/en/ostiblue/search.html>

Note: Advanced search searches Full Record, Title, Author, and Date Range. You cannot specify abstract, so full record was searched. Search is more limited than previous databases.

Table S5. Metadata from Science.gov Search Strategy #5

| Search string | Restrictions | Returns [Date] |
| --- | --- | --- |
| Full Record: (Fish*) AND (“Fresh water” OR Freshwater OR Stream OR Water OR River OR Fluvial OR Estuar* OR Reservoir OR Impoundment OR "Hydro electric" OR Hydroelectric* OR "Hydro dam" OR Hydrodam* OR "Hydro power" OR Hydropower OR "Hydro" OR Dam) AND (Flow* OR Discharg*) AND (Productivity OR Biomass OR Abundance OR Densit* OR Yield OR “Ecological response” OR “Ecosystem response” OR “Biotic response”) NOT (mining OR "mine site" OR aquaculture OR "wastewater treatment" OR carbon) | - 2017-2019 - Advanced search - Sort by Relevance - All categories (except HSDB Hazardous Substances Databank) - Full record search terms - English only search terms - Accepted the additional records - Text records included only (no multimedia found) - Public access – peer-reviewed articles federally funded - Open access – no institutional subscription needed | **512** (+204 public access)  [December 1, 2019] |

***AGRICOLA: Search Strategy #6***

Available online: <https://agricola.nal.usda.gov/vwebv/searchAdvanced>

Note: Article Citation Database. Keywords Anywhere results come from anywhere in the bibliographical record: publisher, contents, note, meeting name, subject heading, sponsoring organization etc. Searching is more limited than other databases.

Table S6. Metadata from AGRICOLA Search Strategy #6

| **Search String** | **Restrictions** | **Returns [Date]** |
| --- | --- | --- |
| Fish? AND (Flow? OR Discharg?) AND (Productivity OR Biomass OR Abundance? OR Densit? OR Yield?) | - 2017-2019 - Searched NAL Article Citation Database - Advanced search - Used “all of these” - Searched within Keyword Anywhere - All Locations - All places - All types - All Formats - All languages - All media | **0**  [October 11, 2019] |

**Search Engine**

Internet searches were conducted in August 2019 using the search engine Google Scholar (first 500 hits sorted by relevance). Potentially useful documents that had not already been found in publication databases or in the systematic map were recorded and screened for inclusion in the review (Table S7).

***Google Scholar: Search Strategy #7***

Available online: <https://scholar.google.com>

Note: Keywords Anywhere results come from anywhere in the article. Searching is more limited than other databases. Number of actual returns exceeds reported, but limited to 500 most relevant.

Table S7. Metadata from Google Scholar Search Strategy #7 without using “Not” statements*

| **Search String** | **Restrictions** | **Returns [Date]** |
| --- | --- | --- |
| Find articles:   - with **all** of the words Fish Flow - with **at least one** of the words: Productivity Biomass Abundance Density Yield   Final search string appears as: Fish Flow Productivity OR Biomass OR abundance OR Density Or Yield | - 2017-2019 - Advanced search - Searched “where my words occur” – anywhere in the article - Search articles (excluding patents, case law, and citations) - Search for pages written in any language - Sort by relevance | **~82800**  **Selected first 500**  [January 8, 2020] |

*The results of this search were included in the final count of Google Scholar articles

**Other literature searches**

A few specialist websites and databases of grey literature were suggested through our calls for literature that had previously not be identified or searched during the systematic map. These sites were searched January and March 2020.

***ARLIS - Susitna Doc Finder: Search Strategy #8***

Available online: <https://www.arlis.org/susitnadocfinder/Search/Advanced>; ARLIS – Alaska Resources Library and Information Services, Susitna Doc Finder – Online SuHydro and SuWa Documents

Note: Advanced Search: All Fields searches title, author, subject, publisher, year of publication, report number, full text and contents. Search uses search operators ALL, ANY or NO.

Table S8. Metadata from ARLIS Search Strategy #8

| Search string | Restrictions | Returns [Date] |
| --- | --- | --- |
| **(All Fields:Fish*) AND (All Fields:“Fresh water” OR Freshwater OR Stream$ OR Water$ OR River$ OR Fluvial OR Estuar* OR Reservoir$ OR Impoundment$ OR "Hydro electric*" OR Hydroelectric* OR "Hydro dam*" OR Hydrodam* OR "Hydro power" OR Hydropower OR "Hydro" OR Dam$) AND (All Fields:Flow* OR Discharg*) AND (All Fields:Productivity OR Biomass OR Abundance$ OR Densit* OR Yield$ OR “Ecological response” OR “Ecosystem response” OR “Biotic response”) NOT ((All Fields:mining OR "mine site" OR aquaculture OR "wastewater treatment" OR carbon))** | - 1904 onwards - Advanced search - Sort by Relevance - All authors, publishers, regions, topics, report types, report numbers - Full record search terms - Used Search Fields and Search Groups | 11161  Selected first 250  [Jan 25, 2020] |

***Federal Energy Regulatory Commission (FERC) eLibrary: Search Strategy #9***

Available online: <https://elibrary-backup.ferc.gov/idmws/search/fercgensearch.asp>; FERC Online eLibrary (formerly FERRIS) – Federal Energy Regulatory Commission eLibrary

Note: Advanced Search: Full Text searches rely upon the content of the FERC PDF, which may be incomplete. Accordingly, not all documents may be returned.

Table S9. Metadata from FERC Search Strategy #9

| Search string | Restrictions | Returns [Date] |
| --- | --- | --- |
| (Fish) AND (“Fresh water” OR Freshwater OR Stream OR Water OR River OR Fluvial OR Estuary OR Reservoir OR Impoundment OR "Hydro electric" OR Hydroelectric OR "Hydro dam" OR Hydrodam OR "Hydro power" OR Hydropower OR "Hydro" OR Dam) AND (Flow OR Discharg) AND (Productivity OR Biomass OR Abundance$ OR Density OR Yield OR “Ecological response” OR “Ecosystem response” OR “Biotic response”) NOT (mining OR "mine site" OR aquaculture OR "wastewater treatment" OR carbon) | - Advanced search - Select Document Date: 01/01/1904 – 12/31/2019 - Search Library: Hydro - Full Text and Description search terms - Document Type for class and type: All - Search only Public availability - Score Desc | **11161**  **Selected only first 250**  [March 16, 2020] |

**Other literature searches**

Reference sections of accepted articles and relevant reviews were hand searched to evaluate relevant titles that were not found using the search strategy. Stakeholders were consulted for insight and advice for new sources of information. We also issued a call for evidence to target sources of grey literature through relevant mailing lists [Canadian Conference for Fisheries Research, American Fisheries Society, WaterPower Canada (formerly Canadian Hydropower Association), Canadian Electricity Association, Ontario Women Anglers, the Mactaquac Aquatic Ecosystem Study, Instream Flow Council] and through social media (e.g., Twitter) in December 2019. The call for evidence was also distributed by the Advisory Team to relevant networks and colleagues.

**References**

Harper, M., T. Rytwinski, J. J. Taylor, J. R. Bennett, K. E. Smokorowski, and S. J. Cooke. 2020. How do changes in flow magnitude due to hydroelectric power production affect fish abundance and diversity in temperate regions? A systematic review protocol. Environmental Evidence 9(1):14.

Rytwinski T, Harper M, Taylor JJ, Bennett JR, Donaldson LA, Smokorowski KE, et al. What are the effects of flow-regime changes on fish productivity in temperate regions? A systematic map. Environmental Evidence. 2020;9:7.
