# Supplementary material for: How do changes in flow magnitude due to hydropower operations affect fish abundance and biomass in temperate regions? A systematic review
Source: Environ Evid. 2022 Feb 4;11:3. doi: 10.1186/s13750-021-00254-8 (PMC8813579; doi:10.1186/s13750-021-00254-8)
Supplement: Supplementary file 4 — Additional file 4. Study validity assessment description. Provides further description of the study validity assessments, along with the critical appraisal tool. [file 13750_2021_254_MOESM4_ESM.docx]

**Additional File 4. Study validity assessment description**

Description: This Additional File provides further description of study validity assessments, along with the critical appraisal tool (Table S1).

**Further description of study validity assessments**

If a study contained more than one project (i.e., the project differed in terms of one or more components of the study validity; Table S1), each project received a separate validity rating and label (i.e., Enders et al. 2017 a/b indicates that there is one study with two projects within the Enders et al. 2017 article). For example, temporal and spatial comparisons [i.e., whether the study compared samples temporally (*BA*), spatially (*CI*) or using a combination (*BACI*)] was an internal study validity criterion (Table S1). If one project within the study had a *BACI* comparison, while the other had a *BA* comparison, they received different internal validity responses for this criterion and therefore a different project label.

The criteria of the appraisal tool are based on internal validity and evaluated the following: (i) study design [i.e., *BACI*, *RCT*, *BA*, *CI*, *ALT-CI*, *RCA* and *NR*, as well as deficient *BA* designs (*DEF_BA*) and incomplete *BACI*s (i.e., *BA* studies with *Before* data from a different site or studies missing certain components of a true *BACI* design; *INCOM_BACI*)], (ii) replication (no replication, pseudoreplication, or true replication), (iii) control and site matching (i.e., how well matched were intervention, comparator and sub-sample sites in terms of habitat, river section etc. at the outset of the study and during sampling), (iv) the intervention metric (quantitative or qualitative changes in magnitude or comparisons of unregulated to regulated systems), (v) confounders (other environmental or anthropogenic changes or other additional manipulations of flow components that occurred after the start of the study) and (vi) methodology (i.e., were different methodologies used between intervention and comparator sites and/or times). Each criterion was scored as ‘Yes’, ‘Partially’, ‘No’ or ‘Unclear’ based on the pre-defined criteria in Table S1. The study was given an overall ‘Low’ validity if it scored ‘No’ or ‘Unclear’ for any one or more of the criteria. If a study did not score ‘No’ for any criteria, but scored ‘Partially’ for one or more criteria, it received an overall ‘Medium’ validity score. Studies for which the answer was ‘Yes’ to all questions were classified as having ‘High’ validity. Each criterion received equal weight during study validity assessment.

Table S1. Critical appraisal tool for study validity assessment. Additions made to the tool since the protocol are indicated in italics.

| **Question/criterion** | **Response to question** | | | | **Type of Bias addressed** |
| --- | --- | --- | --- | --- | --- |
|  | **Yes** | **Partially** | **No** | **Unclear** |  |
| 1. Did the study consist of both temporal and spatial comparisons? | BACI, RCT | INCOM-BACI, BA, CI, ALT-CI, RCA, NR | N/A as study is not eligible for inclusion based on inclusion criteria | *DEF-BA* or lacking sufficient information to judge | selection |
| 2. Are experimental/observational units replicated? | ≥2 independent experimental/observ-ational units (i.e., the level of replication at which the intervention was administered/the exposure experienced). *For BA designs, is there either within-year replication (fish abundance data available for at least 2 months/seasons post-intervention) or interannual replication (fish abundance data available for at least two years post-intervention).* | There were at least two experimental/observ-ational units but there is a lack of independence between these units (pseudoreplication). *N/A for BA designs.* | No replication (i.e., <2 independent experimental/ observational units), *or, for CI designs, the study had replication (either true or pseudo), but (sub)sample sites were too different to be treated as replicates (i.e., major differences in magnitude, morphology or habitat characteristics, and/or the presence of intersecting tributaries). For BA designs, fish abundance data was only available for a single post-intervention sample.* | Lacking sufficient information to judge | selection |
| 3. Are intervention and comparator sites well-matched at site selection and/or study initiation? | *Sites are well matched e.g.:* (1) Intervention and comparator sites are well-matched (i.e., similar physical characteristics), *(2) Sub-sample sites are well-matched within both intervention and comparator sites (e.g., comparator sub-sample sites are all within the free-flowing river section).* N/A for BA designs (i.e., intervention/comparator at same site). | *Sites are moderately matched e.g.:* (1) Intervention and comparator sites are moderately matched, *(2) sub-sample sites are moderately matched within intervention and/or comparator sites (e.g., comparator sub-sample sites are in free-flowing section and river/reservoir transition zones).* N/A for BA designs. | Intervention and comparator sites are poorly matched. N/A for BA designs. | Lacking sufficient information to judge | selection |
| 4. Can the intervention be clearly interpreted? | It is clear that a change to flow magnitude has occurred and quantitative data on magnitude is reported | It is clear that a change to flow magnitude has occurred but either no quantitative data on magnitude is reported or the quantitative data is difficult to interpret (e.g., averaged across intervention and control sites within the same river) | N/A | The study compares an unregulated stream (or section of a stream) to a regulated stream (i.e., regulated via a hydro dam) or reports unspecified multiple components affecting flow (i.e., study does not report effects of components separately to isolate individual impacts of components) | selection, performance, reporting |
| 5. Was the study free of other potential confounders after sample selection/study initiation? | No or minimal confounding factors present, including e.g.: (1) no or minimal differences in environmental conditions between intervention and comparator sites and/or time periods (e.g., unplanned human alterations, floods, droughts, time-related trends), (2) no additional experimental manipulations of other flow regime components (e.g., flow frequency, duration) at the same time as magnitude alterations or, if present, are accounted for appropriately in analysis. | N/A | Confounding factors present that could have an impact on the outcome and these are not accounted for in analysis. | Lacking sufficient information to judge | performance |
| 6. Did the study use similar sampling/ measurement method(s) between intervention and comparators? | Similar/consistent sampling/measurement methods are used between intervention and comparator sites and/or times (e.g., gear type, timing or size of sample areas) | Different/inconsistent sampling/ measurement methods are used between intervention and comparator sites and/or times (e.g., gear type, timing or size of sample areas) | N/A | Lacking sufficient information to judge | detection |
