# Supplementary material for: How do changes in flow magnitude due to hydropower operations affect fish abundance and biomass in temperate regions? A systematic review
Source: Environ Evid. 2022 Feb 4;11:3. doi: 10.1186/s13750-021-00254-8 (PMC8813579; doi:10.1186/s13750-021-00254-8)
Supplement: Supplementary file 6 — Additional file 6. Data extraction considerations. Provides a description of further data extraction considerations, including those for BA study designs. [file 13750_2021_254_MOESM6_ESM.docx]

**Additional File 6. Data extraction considerations.**

Description: Here, we provide further descriptions of data extraction considerations, including considerations for BA study designs.

**Additional data extraction considerations**

First, we defined a *Site* as a specific hydroelectric facility (i.e., hydro dam) where observations or manipulative experiments were conducted and reported in one or more articles (see Table 2 in main report). Each specific hydroelectric facility was given a “Site ID”, using the hydrodam name. If no name was provided, we used the facility name (if provided) or river name. If no dam, facility or single river name was available, we used NR. Different articles reporting information from the same site were numbered to provide a unique identifier (e.g., Rupert1, Rupert2). Second, we defined a *Study* as an experiment or observation that was undertaken over a specific period at particular sites reported as separate waterbodies that were not treated as replicates within the article. When multiple studies were reported within an article, they were entered as independent lines in the database. Study ID included the Site ID plus a letter (e.g., WreckCove_A, WreckCove_B). Third, a single study could also report separate relevant comparisons, defined as *Datasets* for the same or different species, operating conditions, outcomes, life stages, sampling methods, years/seasons post-treatment and/or sites, but otherwise the same meta-data (see Table 2 in main report). Each dataset was reported as a separate line in the database and assigned a Dataset ID that included Study ID plus a number (e.g., WreckCove_A1, WreckCove_A2).

If authors reported responses for the same species and the same outcome category in a single study, with otherwise consistent meta-data, we extracted separate datasets for the database when there were different (i) life stages (e.g., the abundance of eggs for species X and the abundance of age-0 for species X), (ii) residency status (i.e., resident vs. non-resident fish); (iii) years and/or seasons post-treatment within a given outcome category (i.e., if for a given outcome category, multiple time periods were monitored and reported separately for a *CI* study design or within-in year variation post-treatment for a *BA* design); (iv ) sampling methods (e.g., electrofishing and snorkeling), and/or (v) sites downstream of a hydro dam within a single river sampled using a *BA* design. For quantitative analyses, we aggregated these datasets to reduce non-independence (see “Combining data across multiple comparisons within a study” in the main text and Additional File 7).

When a single study reported multiple outcomes within a particular outcome category (i.e., abundance and density or CPUE) but otherwise the same meta-data, for quantitative synthesis we selected the metric closest to the primary focus of the review for each outcome and retained other outcomes only for narrative review (Fig. S1). For example, if both abundance and CPUE were reported within a given study, we selected abundance data for quantitative synthesis and retained both abundance and CPUE in the narrative synthesis. Furthermore, if biomass and yield were reported within a single study, we selected only the biomass dataset for quantitative synthesis but retained both biomass and yield in the narrative synthesis (Fig. S2). In all cases, for quantitative synthesis we worked to maximize replication, selecting outcomes that had true replication or pseudoreplication over outcomes with no replication. If an outcome metric, closer to the primary focus of the systematic review, had no replication, the next, more ‘distant’ metric with greater replication was selected by default for quantitative analysis (i.e., if the study reported unreplicated abundance, but density was pseudoreplicated, density was selected for quantitative analysis). We retained both outcomes for narrative synthesis in this case as well.

While we extracted all possible data from each study, in some cases it was not possible to retain all datasets extracted. When retaining a dataset would lead to double counting of the same individuals, the aggregated datasets were removed. For example, individual species outcomes were selected over grouped species outcomes for narrative and quantitative synthesis (i.e., abundance of *Oncorhynchus mykiss* were selected rather than *Oncorhynchus* spp. abundance) while still maximizing replication. Similarly, individually reported life stages (e.g., adults) were selected over grouped life stages (e.g., mixed life stages), individually reported monthly data were retained over grouped monthly data, and data for individual sites were retained over aggregated site data. The only exception to this occurred if data for multiple months or sites were aggregated and data for a single month or site were also reported. In those instances, replication was maximized and individual site data were not considered for either narrative or quantitative synthesis. Two additional reasons for not retaining datasets for either narrative or quantitative synthesis include: (i) no fish of a specific species were captured at a particular site both *Before* and *After* an intervention occurred, or (ii) when samples were taken in a diversion reach (i.e., area downstream of where water is removed from the system) and an outflow reach (i.e., area downstream of where water is returned to the system) and no *Before* data was available for either the comparator or intervention reaches. To ensure independence of the datasets, and based on stakeholder input, only the diversion reach was retained for quantitative and narrative synthesis.


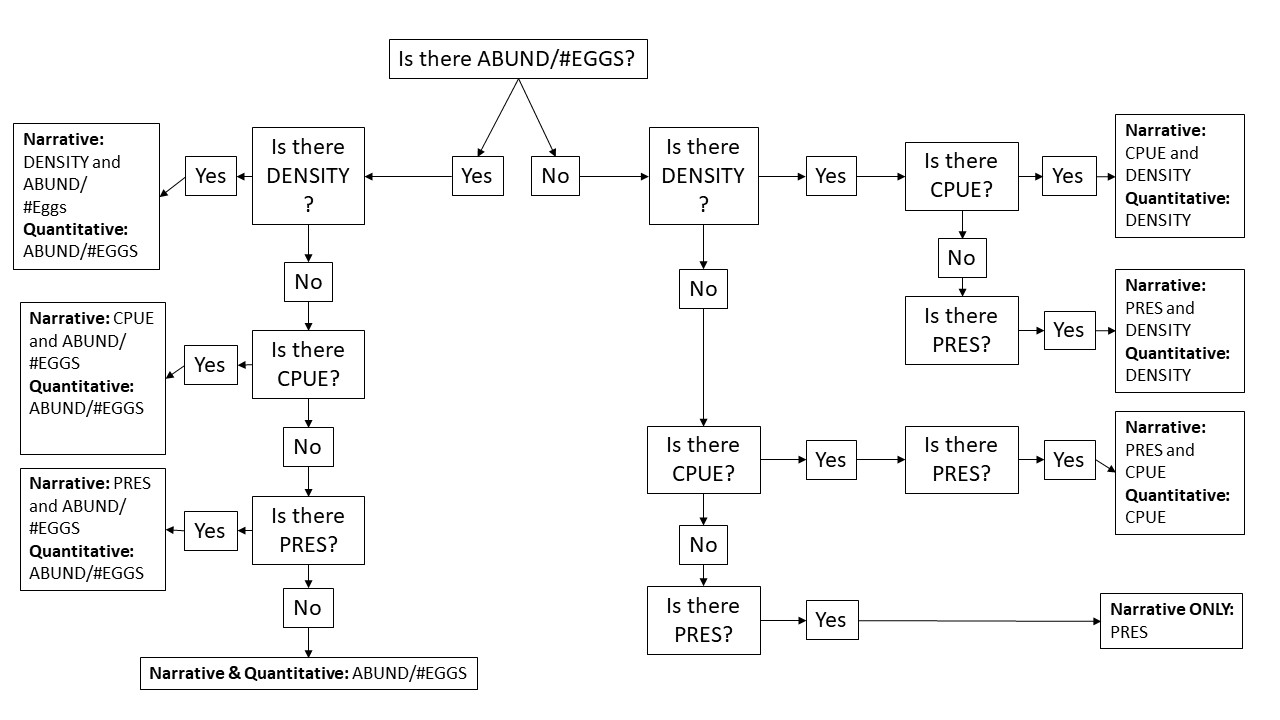


Fig. S1. Decision tree for abundance metrics to assist in determining whether to retain metrics for narrative and/or quantitative synthesis. ABUND: abundance; CPUE: catch per unit effort; DENSITY: density; #EGGS: number of eggs; PRES: presence/absence.


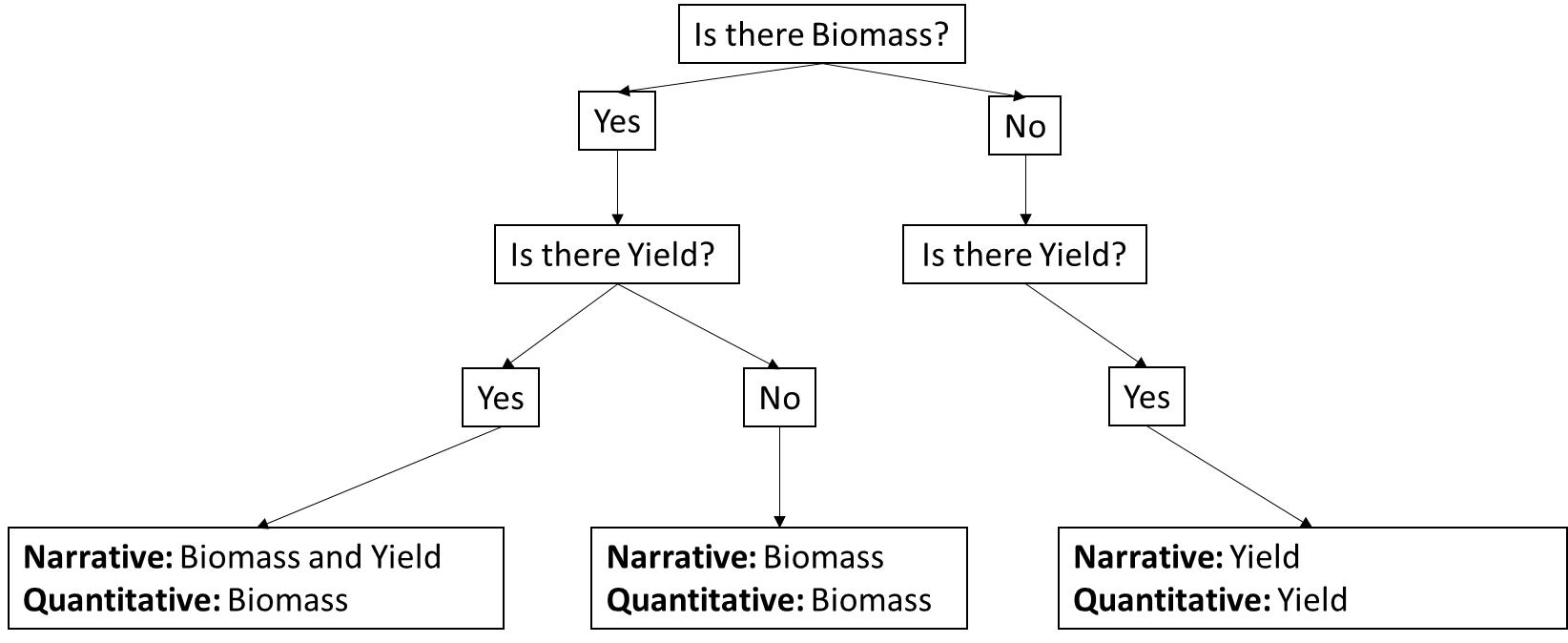


Fig. S2. Decision tree for biomass metrics to assist in determining whether to retain metrics for narrative and/or quantitative synthesis.

### BA data extraction considerations

We decided whether outcome data sampled during a certain calendar year represented *Before* or *After* as follows:

The *Before* period was defined to date back as long as fish outcome data were available. The *Before* period was defined to end with (and include) the last pre-intervention year. Periods without fish outcome data were included in the *Before* period if they lasted no more than five years and were preceded by a year with outcome data. Furthermore, if there was only a single *Before* period of fish outcome data, as long as it occurred within five years of the start of the intervention, the data were included as the *Before* period.

The *After* period was defined to begin with the first post-intervention year and last as long as fish outcome data were available, and no additional changes/modifications to flow magnitude began. If additional changes/modification were made after the initial period, these *After* periods were also retained and compared to both the *Before* period, and the previous *After* period during quantitative analysis. Periods without outcome data were included in the *After* period if they lasted no more than five years and were followed by a year with outcome data.

In cases where a gap greater than five years occurred, data were extracted, but considered as a deficient *Before/After* comparisons during critical appraisal, and the effect of retaining these data was explored using sensitivity analysis during quantitative analysis. Gaps longer than five years occurred in four studies (two studies with a *Before* period gap, and two studies with an *After* period gap).

Temporal replication was considered at two levels: (i) within-year (n = # months), and (ii) interannual (n = # years). For within year variation, each *After* year was extracted as a separate row (i.e., different datasets from the same study), with the mean fish outcome and variation for each *After* time period coming from within-year sampling (e.g., averaged across sampling months or seasons). If fish were sampled for only one *Before* year (but for >1 month or season), that *Before* within-year mean and variation were used as the comparator for each separate *After* year. If there were multiple within-year time periods (i.e., >1 year and each year fish were sampled in >1 month), we used the most recent *Before* time period (within-year mean and variation) and recorded this for each separate *After* period. We accounted for multiple comparisons to the same *Before* year during quantitative analysis.

When fish outcome data were available for more than one year in a *Before*/*After* design, interannual replication and calculation of interannual variation allowed us to include these data in separate analyses even if no usable information was available on within-year variation (i.e., when a single fish sampling period occurred per year over >1 years, or when only a total fish abundance for multiple within-year sampling periods was reported for >1 years). Treating within-year and interannual variation separately ensured we did not introduce bias by considering only interannual variation, if within-year and interannual variation differed. For example, if it was suspected that within-year variation in fish abundance was larger than the interannual variation, using effect sizes with interannual variation only would lead to a lower variance and would be given a higher weight in meta-analysis than if the within-year variation had been known and included as well. Calculations of interannual variation followed two scenarios. First, if (a) fish outcomes were only sampled once per year, or (b) studies only report total fish abundance from multiple sampling seasons within a given year, then mean fish abundance and variation were calculated by averaging these data across all *Before* years (n = # *Before* years), and all *After* years (n= # *After* years). Second, if fish abundance was sampled/reported more than once per year, average abundance was calculated per year (or used in the case where authors reported this average), then averaged across all *Before* years (n = # *Before* years) and all *After* years (n=# *After* years). In the latter case, we were able to make use of studies that reported average fish abundance (from multiple within-year samples) but did not provide any information on within-year variation which would have precluded inclusion in the within-year variation analysis above.

If *BA* study designs were carried out at multiple waterbodies and these waterbodies were not treated as replicates within a given article (i.e., fish responses to changes in magnitude were reported separately for each waterbody rather than combined in a single analysis as replicates), each waterbody was treated as a separate study (same article ID, different Study ID). If a single waterbody was sampled in a *BA* design but at multiple locations downstream of the hydropower dam, each sampling location was extracted on separate rows and treated as the same study [same Study ID, different Dataset ID - similar to different years/seasons sampled in *CI* designs; these were later aggregated to reduce non-independence for quantitative synthesis (see Additional File 7: Combining data across multiple comparisons within a study)]. One exception was when both a diversion and a return section were considered in the same study. If this occurred (and a comparator site was available), data from all sites were extracted on separate rows and treated as the same study (same Study ID, different Dataset ID), but the diversion and return sections were not combined using a composite effect size. If studies combined multiple sites within a given waterbody (i.e., total or average fish abundance), all data were extracted and noted in a comment, then a single metric, maximizing replication was selected for quantitative analysis.
