# Supplementary material for: How do changes in flow magnitude due to hydropower operations affect fish abundance and biomass in temperate regions? A systematic review
Source: Environ Evid. 2022 Feb 4;11:3. doi: 10.1186/s13750-021-00254-8 (PMC8813579; doi:10.1186/s13750-021-00254-8)
Supplement: Supplementary file 7 — Additional file 7. Data preparation and additional calculations for quantitative synthesis. Provides a description of data preparation for quantitative synthesis in relation to converting BACI studies into CI or BA designs, reducing multiple effect size estimates from the same study, and our handling of pseudoreplication. [file 13750_2021_254_MOESM7_ESM.docx]

**Additional File 7. Data preparation and additional calculations for quantitative synthesis**

Description: Here, we provide further descriptions of data preparation for quantitative synthesis in relation to converting BACI studies into CI or BA designs, reducing multiple effect size estimates from the same study, and our handling of pseudoreplication.

**Initial Data Preparation**

Outcomes from *BACI* studies were converted to *CI* or *BA* prior to quantitative synthesis to permit analysis with the selected effect size calculations. To convert a *BACI* to a *CI*, data sampled before the intervention (*B*) was subtracted from data sampled after the intervention (*A*) for each comparator (*C*) and intervention (*I*) site [i.e., *C*: *A-B* and *I*: *A-B*]. To convert to a *BA*, data sampled in the comparator site (*C*) were subtracted from data sampled in the intervention site for the *B* and *A* years [i.e., *B*: *I-C* and *A*: *I-C*]. Means and variances were obtained by averaging across sites or years in each group. In all cases, we worked to maximize the resulting sample size during conversion.

**Combining data across multiple comparisons within a study**

To reduce multiple effect size estimates from the same study, and avoid giving studies with multiple estimates more weight in analyses, datasets were aggregated in five instances when studies sharing all other meta-data, reported: (i) responses from multiple life stages separately within the same outcome (e.g., the abundance of eggs for species X, and the abundance of age-0 for species X, separately) (seven studies); (ii) years and/or seasons post-treatment within a given outcome category (i.e., if for a given outcome category, multiple seasons were monitored and reported separately for a *CI* study design (one study; not included for meta-analysis due to lack of replication) or within-in year variation post-treatment for a *BA* design) (four studies); (iii) different sampling methods (no studies), (iv) sites downstream of a hydro dam within a single river sampled using a *BA* design (13 studies), and (v) for one study in which data for both resident and non-resident individuals of the same species were reported and aggregated. Responses could have been summed if sampling was conducted in the same time period; however, in all instances where this occurred, aggregation would have led to an inflated sample size and required an unacceptable level of data manipulation.

With respect to situation (i) above, most cases were from sampling that was conducted at different time periods within a calendar year (e.g., the abundance of eggs were collected in spring and the abundance of YOY later in summer), potentially resulting in the same individuals being sampled at different time periods and leading to a lack of independence between responses. If sampling was conducted at the same time period, responses could have been summed using equations 23.1-23.3 in Borenstein et al. [1]; in the one *CI* instance where this occurred it was not possible to sum the responses of the different life stages for three reasons: (1) this study was a *BACI* conversion to *CI*; (2) outcomes were reported as densities; (3) intervention sites were pseudo-replicates. If results had been summed, this would have led to an inflated sample size and required an unacceptable level of data manipulation. Therefore, we aggregated such cases instead.

With respect to situation (ii) (i.e., aggregating by years and/or seasons post-treatment within a given outcome category) we attempted to aggregate across seasons, but only a single study with insufficient replication reported seasonal data requiring aggregation. If data were reported for multiple post-treatment years for a *CI* and magnitude remained status quo (meaning no additional changes occurred since time period/year-1), we only included the first year post treatment (year-1) in the main analysis but extracted separate datasets for all cases where there were more years of data, in order to explore the possibility of a temporal lag in fish responses to flow magnitude alterations.

When aggregation was necessary, in each of the cases discussed above, we computed an average effect size. To do so, we first computed the arithmetic mean effect size as the mean of the effect sizes from different comparisons within a study according to equation 24.1 in Borenstein et al. [2]. The variance of this average effect size was then computed following methods of Borenstein et al. [2]. Because the correlations between comparisons were unknown, we assumed a correlation coefficient of *r*=1. This assumption may lead to overestimation of variance and result in an increased likelihood of a Type II error (i.e., finding that the effect size is not significantly different from zero).

In some cases, more than one situation for aggregating datasets was present (i.e., multiple life stages and downstream sites were reported separately). When this occurred, we did not average across both life stages and downstream sites, because outcomes were already averaged across years, leading to unacceptable levels of data manipulation. To avoid this, we selected the life stage that was less likely to be under-represented by the sampling technique used (i.e., catchability by electrofishing increases with size [3,4]). Similarly, if a single species was sampled in one location, but different life stages were captured using different techniques, we aggregated by life stage, disregarding the sampling technique, under the assumption that the authors selected the sampling technique that was most effective for each life stage targeted.

**Adjustment accounting for pseudoreplication**

Replication within a *control/impact* study (i.e., group sample size) was considered at two levels: (i) independent intervention areas (i.e., separate waterbodies or separate sections of a waterbody receiving treatment – true replicates), and (ii) partly subsampled data, hereafter referred to as pseudoreplicated samples [i.e., in the sense that reported variances did not refer to the variability of true replicate means from (i) above but to the variability of subsamples within/across true replicates]. For the former, we recorded the number of independent intervention areas as the level of true treatment replication. For the latter, we recorded the number of pseudoreplicated samples occurring, for example, at the sub-sample site within an area (i.e., non-independent replicates). In cases of pseudoreplicated data (or presumed pseudoreplicated data), we made appropriate adjustments in the quantitative synthesis.

To avoid giving pseudoreplicated data too much weight in analyses (i.e. outcome means and variances were not from independent replicates but subsampled sites), we calculated the variance of effect sizes using a modified equation and a conservative sample number [5,6].

1. Standard errors of the mean outcomes for each group were converted to standard deviations using the total numbers of subsamples as sample sizes (or reported standard deviations based on the total number of subsamples that were left unchanged).
2. Effect sizes (Hedges’ *g* statistic) that were also calculated using the total number of subsamples [7]:

$$d=\frac{\bar{X}_{G2}- \bar{X}_{G1}}{S_{\mathrm{pooled}}}$$

where$\bar{X}_{G1}$ and $\bar{X}_{G2}$ were the means of group 1 (G1 = comparator group) and group 2 (G2 = intervention group). S_pooled_ was the pooled standard deviation of the two groups:

$$S_{\mathrm{pooled}}= \sqrt{\frac{\left( n_{G2}-1 \right)S_{G2}^{2}+\left( n_{G1}-1 \right)S_{G1}^{2}}{n_{G1}+ n_{G2}-2}}$$

where *S* = standard deviation, and n_G2_ and n_G2_ were the number of subsamples of group 1 and group 2 (e.g., number of subplots or nests).

To convert from *d* to Hedges’ *g,* we used a correction factor that removes small sample size bias:

$$J=\left[ 1-\frac{3}{4\left( n_{G1}+n_{G2}-2 \right)-1} \right]$$

here again, n_G1_ and n_G2_ were the number of subsamples of group 1 and group 2.

1. In the following equation, variances for *d* (i.e., V_d_) were calculated using both the conservative sample number (solid-lined box; nt_G1_ and nt_G2_) and the total number of subsamples as sample sizes (dashed-lined box; n_G1_ and n_G2_):

$$V_{d}= \frac{{nt}_{G1}+{nt}_{G2}}{{{nt}_{G1}nt}_{G2}}+ \frac{d^{2}}{2\left( n_{G1}+n_{G2} \right)}$$

This conservative sample number was based on using the number of true replicates instead of pseudoreplicates. For this review, in all cases, the conservative sample number was one replicate waterbody (i.e., nt_G1_ =1 and nt_G2_=1) where plot or nest subsamples were taken from.

1. Then Hedges’ *g* and associated variance (*V_g_*) were calculated as:

$Hedge^{'}s g=J \times d$

$$V_{g}=J^{2} \times V_{d}$$

Therefore, the total number of subsamples was used to calculate an effect size estimate and the conservative sample number was used to calculate the uncertainty of this estimate (i.e., effect size weighting). This method provides a conservative estimate of variability. We used sensitivity analyses to determine the influence of including pseudoreplicated studies on the overall mean effect size.

**Missing Variance**

When standard deviations were missing and it was not possible to calculate from reported data (i.e., by calculating from reported raw data, converting from standard error or 95% confidence interval), mean value imputation was used to calculate the missing variables:

$$SD= \bar{X_{j}}\left( \frac{\sum_{G2}^{k} {SD}_{G2}}{\sum_{G2}^{K} \bar{X_{G2}}} \right)$$

where $\bar{X}_{j}$ is the observed mean of the dataset with missing information, and *k* is the number of j studies with complete information. All datasets with standard deviations were used in imputation, unless effect sizes were calculated through aggregation. Imputation methods have been found to perform as well, or better, than other ways of dealing with missing data values as long as the datasets with missing variables did not exceed 60% of the total [8]. In our case, two *CI* studies (eight cases, of which six cases were aggregated resulting in three datasets) had missing standard deviations that could not be calculated from reported data. This is equal to 3% of all *CI* study datasets, allowing us to confidently use imputation to fill these data gaps. To determine the impact of imputation, we compared the summary effect sizes with and without datasets with imputation using sensitivity analysis.

**References**

1. Borenstein M, Hedges LV, Higgins JPT, Rothstein HR. Recreating the summary data for the full study (option 2). In: Borenstein M, Hedges LV, Higgins JPT, Rothstein HR, editors. Introduction to Meta-Analysis. United Kingdom: Johgn Wiley & Sons, Ltd.; 2009. p. 221–2.

2. Borenstein M, Hedges LV, Higgins JPT, Rothstein HR. Multiple outcomes or time-points within a study. In: Borenstein M, Hedges LV, Higgins JPT, Rothstein HR, editors. Introduction to Meta-analysis. United Kingdom: John Wiley & Sons, Ltd; 2009. p. 225–38.

3. Borgstroem R, Skaala O. Size-dependent catchability of brown trout and Atlantic salmon parr by electrofishing in a low conductivity stream. Nordic journal of freshwater research. 1993;6:14–20.

4. Hedger RD, Diserud OH, Sandlund OT, Saksgård L, Ugedal O, Bremset G. Bias in estimates of electrofishing capture probability of juvenile Atlantic salmon. Fisheries Research. 2018;208:286–95.

5. Bernes C, Macura B, Jonsson BG, Junninen K, Müller J, Sandström J, et al. Manipulating ungulate herbivory in temperate and boreal forests: effects on vegetation and invertebrates. A systematic review. Environmental Evidence. 2018;7:13.

6. Eales J, Haddaway NR, Bernes C, Cooke SJ, Jonsson BG, Kouki J, et al. What is the effect of prescribed burning in temperate and boreal forest on biodiversity, beyond pyrophilous and saproxylic species? A systematic review. Environmental Evidence. 2018;7:19.

7. Borenstein M, Hedges LV, Higgins JPT, Rothstein HR. Effect sizes based on means. In: Borenstein M, Hedges LV, Higgins JPT, Rothstein HR, editors. Introduction to Meta-analysis. United Kingdom: John Wiley & Sons, Ltd; 2009. p. 21–32.

8. Kambach S, Bruelheide H, Gerstner K, Gurevitch J, Beckmann M, Seppelt R. Consequences of multiple imputation of missing standard deviations and sample sizes in meta-analysis. Ecology and Evolution. 2020;10:11699–712.
