# Supplementary material for: How do changes in flow magnitude due to hydropower operations affect fish abundance and biomass in temperate regions? A systematic review
Source: Environ Evid. 2022 Feb 4;11:3. doi: 10.1186/s13750-021-00254-8 (PMC8813579; doi:10.1186/s13750-021-00254-8)
Supplement: Supplementary file 8 — Additional file 8. Correlation analyses of moderators (Pearson’s χ2) and outlier investigations. Contains results of contingency analysis for independence of moderators, and mixed-effects model comparison when including monitoring duration with and without two extreme outliers. [file 13750_2021_254_MOESM8_ESM.docx]

**Additional File 8.** **Correlation analyses of moderators (Pearson’s χ2) and outlier investigations.**

Description: Results of contingency analysis for independence of moderators, and mixed-effects model comparison when including monitoring duration with and without two extreme outliers

**Moderator analysis – *Control/Impact* Studies**

***Abundance***

Table S1. Pearson chi-squared values (above diagonals), and their *p*-values (below diagonals) of contingency analysis for independence of moderators* considered for *CI* study designs and abundance.

| **Moderator(s)** |  |  |  |  |  |  |  |  |  |
| --- | --- | --- | --- | --- | --- | --- | --- | --- | --- |
|  | Dam size | Hydropower operational regime | Direction of flow magnitude alteration | Alterations to other flow components | Sampling method | Sampling season | Type of comparator (spatial) | Time since intervention | Monitoring duration |
| Dam size | - | 29.22 (77) | 1.92 (76) | 30.83 (77) | 47.35 (73) | 65.82 (77) | 3.7 (76) | 29.95 (77) | 53.46 (75) |
| Hydropower operational regime | <0.0001 | - | 45.75 (76) | 11.36 (77) | 2.94 (73) | 40.81 (77) | 41.78 (76) | 82.56 (77) | 53.28 (75) |
| Direction of flow magnitude alteration | 0.382 | <0.0001 | - | 6.33 (76) | 0 (72) | 10.31 (76) | 43.89 (75) | 52.25 (76) | 29.38 (74) |
| Alterations to other flow components | <0.0001 | 0.023 | 0.042 | - | 38.23 (73) | 31.62 (77) | 12.52 (76) | 10.92 (76) | 314.32 (75) |
| Sampling method | <0.0001 | 0.230 | 1.000 | <0.0001 | - | 41.27 (73) | 8.92 (73) | 10.01 (73) | 34.99 (71) |
| Sampling season | <0.0001 | <0.0001 | 0.016 | <0.0001 | <0.0001 | - | 0.06 (76) | 24.18 (77) | 36.75 (75) |
| Type of comparator (spatial) | 0.157 | <0.0001 | <0.0001 | 0.002 | 0.004 | 0.996 | - | 65.06 (76) | 23.17 (75) |
| Time since intervention | <0.0001 | <0.0001 | <0.0001 | 0.028 | 0.007 | 0.0005 | <0.0001 | - | 40.32 (75) |
| Monitoring duration | <0.0001 | <0.0001 | <0.0001 | 0.006 | <0.0001 | <0.0001 | <0.0001 | <0.0001 | - |

*******(i) Intervention related moderators: dam size, hydropower operational regime, direction of flow magnitude alteration; (ii) confounder related moderators: alterations to other flow components, time since intervention; (iii) study design related moderators: sampling method, sampling season, type of comparator (spatial), monitoring duration.

**Moderator analysis – Interannual *Before/After* Studies**

***Abundance***

Table S2. Pearson chi-squared values (above diagonals), and their *p*-values (below diagonals) of contingency analysis for independence of moderators* considered for *BA* study designs and abundance.

| Moderator(s) |  |  |  |  |  |  |  |  |
| --- | --- | --- | --- | --- | --- | --- | --- | --- |
|  | Hydropower Operational regime | Direction of flow magnitude change | Alterations to other flow components | Sampling method | Sampling season | Type of comparator (temporal) | Time since intervention | Life stage |
| Hydropower Operational regime | - | 68.32 (112) | 78.52 (112) | 13.65 (112) | 93.14 (112) | 42.18 (112) | 73.11 (111) | 61.33 (108) |
| Direction of flow magnitude change | <0.0001 | - | 121.47 (112) | 15.03 (112) | 53.68 (112) | 50.3 (112) | 38.07 (111) | 45.56 (108) |
| Alterations to other flow components | <0.0001 | <0.0001 | - | 11.5 (112) | 46.34 (112) | 69.16 (112) | 44.36 (111) | 51.26 (108) |
| Sampling method | 0.136 | 0.090 | 0.243 | - | 65.89 (112) | 9.69 (112) | 9.28 (111) | 30.44 (108) |
| Sample season | <0.0001 | <0.0001 | <0.0001 | <0.0001 | - | 14.09 (112) | 89.84 (111) | 54.04 (108) |
| Type of comparator (temporal) | <0.0001 | <0.0001 | <0.0001 | 0.021 | 0.007 | - | 25.24 (111) | 22.97 (108) |
| Time since intervention | <0.0001 | <0.0001 | <0.0001 | 0.158 | <0.0001 | <0.0001 | - | 24.18 (107) |
| Life stage | <0.0001 | <0.0001 | <0.0001 | 0.002 | <0.0001 | 0.0001 | 0.002 | - |

*******(i) Intervention related moderators: hydropower operational regime, direction of flow magnitude alteration; (ii) confounder related moderators: alterations to other flow components, time since intervention, life stage; (iii) study design related moderators: sampling method, sampling season, type of comparator (spatial), monitoring duration.

***Interannual BA: Abundance - Meta-regression***

Due to two extreme outliers in effect sizes (*Ictalurus punctatus* and *Micropterus dolomieu*; (Bestgen et al. 2006), we were unable to achieve normality through transformation for the continuous moderator ‘monitoring duration’. We therefore conducted meta-regression with and without these outliers and present results for both analyses below (Fig. S1 and S2). We found no significant relationship of fish abundance and monitoring duration in either instance and the results of the two models did not differ greatly. We report results of *Q_M_* for the models in Table S3. Potential reasons for the occurrence of these outliers for *Ictalurus punctatus* (ES = 14) and *Micropterus dolomieu* (ES = 21), may be due to factors other than flow magnitude alterations that occurred in the *Before* and *After* periods of the study (Bestgen et al. 2006). During the *Before* sampling period, *I. punctatus* had the lowest numbers ever recorded in the history of sampling in the system; this results in a comparison between the *After* period and a non-representative *Before* period which might inflate the effect size. *Micropterus dolomieu* is an established invader with an active removal program, conducted throughout the *Before* and *After* periods. This species had a very successful spawning year during the *After* sampling period, probably due to warm water temperatures, which led to an extreme number of age-1 fish (Bestgen et al. 2006). The *Before* period may have had a depressed number of fish due to active removal and the *After* period had an unexpectedly high number of age-1 fish, resulting in an inflated effect size.


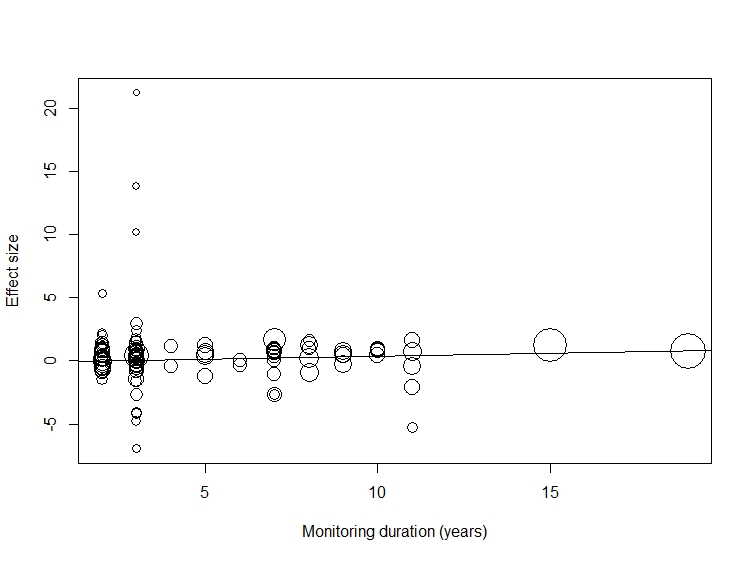


Fig. S1. Meta-regression of effect sizes (Hedge’s *g*) against monitoring duration (years) for interannual *Before/After* studies and abundance. Two extreme effect sizes were retained in this model to compare to model without outliers.


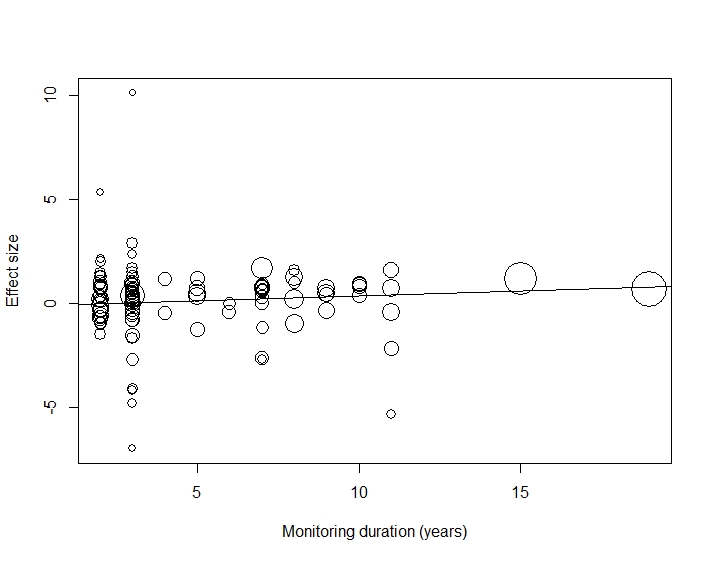


Fig. S2. Meta-regression of effect sizes (Hedge’s *g*) against monitoring duration (years) for interannual *Before/After* studies and abundance. Two extreme effect sizes were removed to improve model fit.

Table S3. Summary results of meta-regression using subsets of fish abundance effect sizes for interannual *Before/After* studies, testing the influence of monitoring duration with and without extreme outliers.

| **Moderator** | ***k*** | ***Q* statistic (p-value)** | ***Q_M_* (*p*-value)** | ***Q_E_* (*p*-value)** |
| --- | --- | --- | --- | --- |
| Monitoring duration (with outliers)  Unmoderated model  Monitoring duration | 112  112 | **421.12 (*p*<0.0001)**  - | -  1.32 (*p*=0.252) | -  **417.43 (*p*<0.0001)** |
| Monitoring duration (without outliers)  Unmoderated model  Monitoring duration | 110  110 | **361.71 (*p*<0.0001)**  - | -  1.39 (*p*=0.239) | -  **357.43 (*p*<0.0001)** |

Unmoderated model: random-effects model; *k*: number of effect sizes; *Q* statistic: value of homogeneity test; *Q_m_*: omnibus test statistic of moderators; *Q_E_*: unexplained heterogeneity. Significance at *p* < 0.05; * Significance at *p*

<0.1.

**References**

Bestgen, K. R., K. A. Zelasko, R. I. Compton, and T. Chart. 2006. Responses of the Green River fish community to changes in flow and temperature regimes from Flaming Gorge Dam since 1996 based on sampling conducted from 2002 to 2004. Larval Fish Laboratory, Colorado State University, Final Report to the Colorado River Recovery Implementation Program, Project 115, Denver.
