# Supplementary material for: How do changes in flow magnitude due to hydropower operations affect fish abundance and biomass in temperate regions? A systematic review
Source: Environ Evid. 2022 Feb 4;11:3. doi: 10.1186/s13750-021-00254-8 (PMC8813579; doi:10.1186/s13750-021-00254-8)
Supplement: Supplementary file 10 — Additional file 10. Additional data descriptions for narrative and quantitative syntheses. Contains further descriptions of data for narrative synthesis (including details on study location, fish species list, and interventions) and quantitative synthesis (including details on interventions and outcomes). [file 13750_2021_254_MOESM10_ESM.docx]

**Additional File 10.** **Additional data descriptions for narrative and quantitative syntheses.**

Description: Further descriptions of data for narrative synthesis (including details on study location, fish species list, and interventions) and quantitative synthesis (including details on interventions and outcomes).

### Narrative synthesis

### Study location


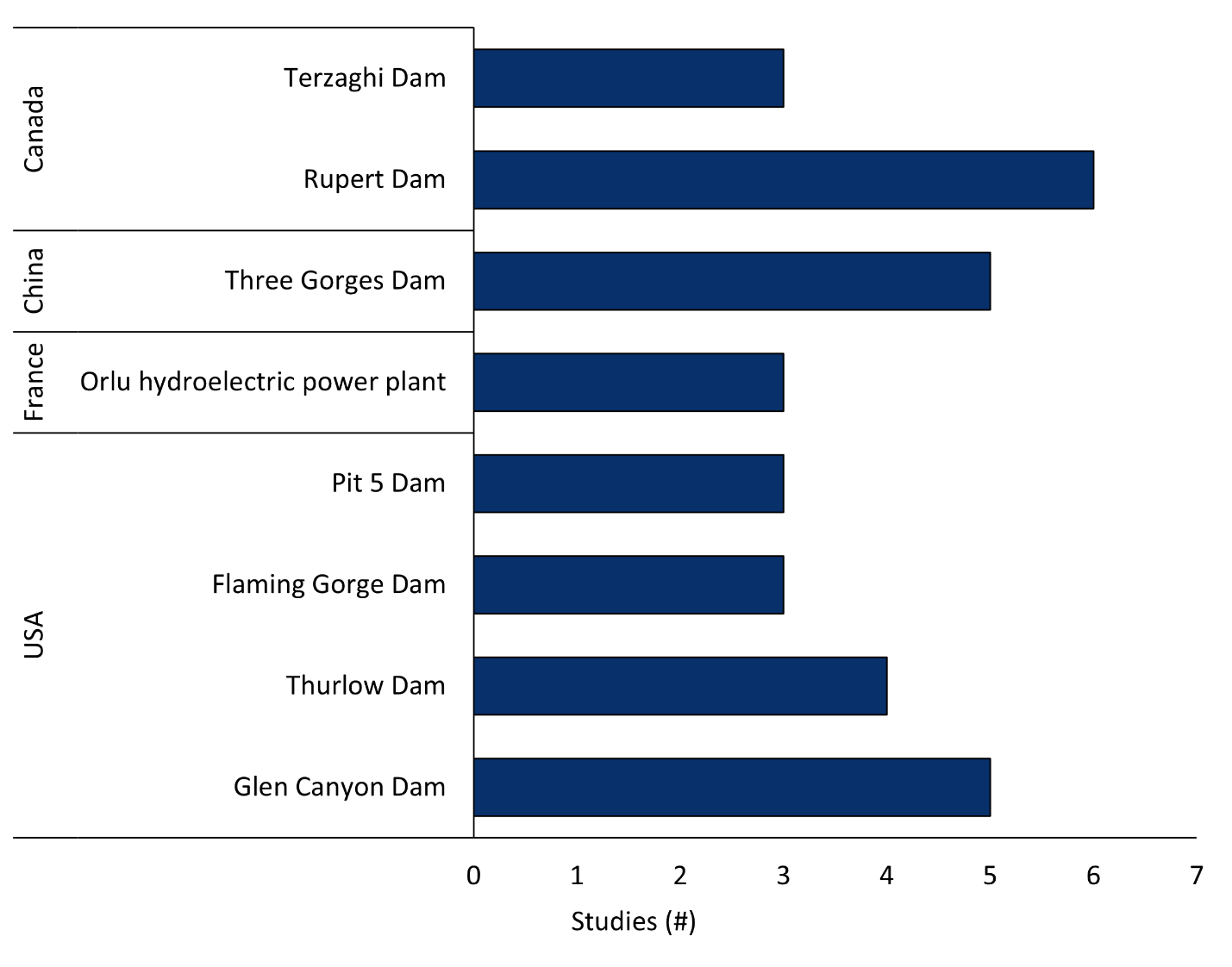


Fig. S1. Eight most studied hydroelectric dams/facilities by country.

### Population

The maximum number of species considered by a single study was 85 (Thurlow Dam, Tallapoosa River, Alabama USA). Of studies that reported species-specific results, two studies did not report enough information to determine which family group a recorded species or species group belonged to (i.e., undefined larval stage). Six studies did not report sufficient information to determine the genus either because it was not possible to identify beyond family for the life stage (two studies), or results were for mixed-genus groups. Nine studies did not report species names but grouped species under genera. Twelve studies reported data that were grouped across family or genus. These studies were not considered in Fig. 6 (in the main text of the review) but did include an additional 30 species not previously reported by articles with species-specific results (Table S1). A single study considered an identified hybrid.

Table S1. Fish species list with all family, genera and species included in the narrative synthesis.

| **Family** | **Genus** | **Species** | **Common name** |
| --- | --- | --- | --- |
| Acestrorhynchidae | Acestrorhynchus | *Acestrorhynchus pantaneiro* | [unk] |
| Acipenseridae | Acipenser | *Acipenser fulvescens* | Lake sturgeon |
|  |  | *Acipenser sinensis* | Chinese sturgeon |
| Amiidae | Amia | *Amia calva* | Bowfin |
| Anguillidae | Anguilla | *Anguilla anguilla* | European eel |
|  |  | *Anguilla australis* | Short-finned eel |
|  |  | *Anguilla reinhardtii* | Speckled longfin eel |
|  |  | *Anguilla rostrata* | American eel |
| Anostomidae | Leporinus | *Leporinus amae* | [unk] |
|  | Schizodon | *Schizodon nasutus* | [unk] |
| Aphredoderidae | Aphredoderus | *Aphredoderus sayanus* | Pirate perch |
| Atherinopsidae | Labidesthes | *Labidesthes sicculus* | Brook silverside |
| Belonidae | Strongylura | *Strongylura marina* | Atlantic needlefish |
| Catostomidae | Carpiodes | *Carpiodes carpio* | River carpsucker |
|  |  | *Carpiodes cyprinus* | Quillback |
|  |  | *Carpiodes velifer* | Highfin carpsucker |
|  | Catostomus | *Catostomus catostomus* | Longnose sucker |
|  |  | *Catostomus columbianus* | Bridgelip sucker |
|  |  | *Catostomus commersonii* | White sucker |
|  |  | *Catostomus discobolus* | Bluehead sucker |
|  |  | *Catostomus latipinnis* | Flannelmouth sucker |
|  |  | *Catostomus macrocheilus* | Largescale sucker |
|  |  | *Catostomus occidentalis* | Sacramento sucker |
|  |  | *Catostomus platyrhynchus* | Mountain sucker |
|  | Cycleptus | *Cycleptus elongatus* | Blue sucker |
|  | Erimyzon | *Erimyzon oblongus* | Eastern creek chubsucker |
|  | Hypentelium | *Hypentelium etowanum* | Alabama hog sucker |
|  |  | *Hypentelium nigricans* | Northern hog sucker |
|  | Ictiobus | *Ictiobus bubalus* | Smallmouth buffalo |
|  |  | *Ictiobus cyprinellus* | Bigmouth buffalo |
|  | Minytrema | *Minytrema melanops* | Spotted sucker |
|  | Moxostoma | *Moxostoma anisurum* | Silver redhorse |
|  |  | *Moxostoma breviceps* | Smallmouth redhorse |
|  |  | *Moxostoma carinatum* | River redhorse |
|  |  | *Moxostoma collapsum* | Notchlip redhorse |
|  |  | *Moxostoma duquesnei* | Black redhorse |
|  |  | *Moxostoma erythrurum* | Golden redhorse |
|  |  | *Moxostoma macrolepidotum* | Shorthead redhorse |
|  |  | Moxostoma pappillosum | V-lip redhorse |
|  |  | Moxostoma pisolabrum | Pealip redhorse |
|  |  | *Moxostoma poecilurum* | Blacktail redhorse |
|  | Xyrauchen | *Xyrauchen texanus* | Razorback sucker |
| Centrarchidae | Ambloplites | *Ambloplites ariommus* | Shadow bass |
|  |  | *Ambloplites rupestris* | Rock bass |
|  | Centrarchus | *Centrarchus macropterus* | Flier |
|  | Lepomis | *Lepomis auritus* | Redbreast sunfish |
|  |  | *Lepomis cyanellus* | Green sunfish |
|  |  | *Lepomis gibbosus* | Pumpkinseed |
|  |  | *Lepomis gulosus* | Warmouth |
|  |  | *Lepomis humilis* | Orangespotted sunfish |
| Centrarchidae | Lepomis | *Lepomis macrochirus* | Bluegill |
|  |  | *Lepomis megalotis* | Longear sunfish |
|  |  | *Lepomis microlophus* | Redear sunfish |
|  |  | *Lepomis miniatus* | Redspotted sunfish |
|  |  | *Lepomis punctatus* | Spotted sunfish |
|  | Micropterus | *Micropterus coosae* | Redeye bass |
|  |  | *Micropterus dolomieu* | Smallmouth bass |
|  |  | *Micropterus punctulatus* | Spotted bass |
|  |  | *Micropterus salmoides* | Largemouth black bass |
|  | Pomoxis | *Pomoxis annularis* | White crappie |
|  |  | *Pomoxis nigromaculatus* | Black crappie |
| Characidae | Astyanax | *Astyanax fasciatus* | Banded astyanax |
|  |  | *Astyanax gr. Scabripinnis* | [unk] |
|  |  | *Astyanax jacuhiensis* | [unk] |
|  | Bryconamericus | *Bryconamericus iheringii* | [unk] |
|  |  | *Bryconamericus stramineus* | [unk] |
|  | Oligosarcus | *Oligosarcus jenynsii* | [unk] |
| Clupeidae | Alosa | *Alosa aestivalis* | Blueback shad |
|  |  | *Alosa alabamae* | Alabama shad |
|  |  | *Alosa chrysochloris* | Skipjack shad |
|  |  | *Alosa pseudoharengus* | Alewife |
|  |  | *Alosa sapidissima* | American shad |
|  | Dorosoma | *Dorosoma cepedianum* | American gizzard shad |
|  |  | *Dorosoma petenense* | Threadfin shad |
|  | Potamalosa | *Potamalosa richmondia* | Freshwater herring |
| Cobitidae | Cobitis | *Cobitis maroccana* | [unk] |
|  |  | *Cobitis taenia* | Spined loach |
|  | Misgurnus | *Misgurnus fossilis* | Weatherfish |
|  | Sinibotia | *Sinibotia superciliaris* | [unk] |
| Cottidae | Cottus | *Cottus asper* | Prickly sculpin |
|  |  | *Cottus bairdii* | Mottled sculpin |
|  |  | *Cottus caeruleomentum* | Blue ridge sculpin |
|  |  | *Cottus carolinae* | Banded sculpin |
|  |  | *Cottus cognatus* | Slimy sculpin |
|  |  | *Cottus confusus* | Shorthead Sculpin |
|  |  | *Cottus gobio* | Bullhead |
|  |  | *Cottus hubbsi* | Columbia Sculpin |
|  |  | *Cottus hypselurus* | Ozark sculpin |
|  |  | *Cottus klamathensis* | Marbled sculpin |
|  |  | *Cottus pitensis* | Pit sculpin |
|  |  | *Cottus ricei* | Spoonhead sculpin |
|  |  | *Cottus tallapoosae* | [unk] |
| Curimatidae | Steindachnerina | *Steindachnerina sp.* | [unk] |
| Cyprinidae | Abramis | *Abramis brama* | Freshwater bream |
|  |  | *Abramis sapa* | White-eye bream |
|  | Achondrostoma | *Achondrostoma arcasii* | [unk] |
|  | Alburnoides | *Alburnoides bipunctatus* | Schneider |
|  | Alburnus | *Alburnus alburnus* | Bleak |
|  | Aspius | *Aspius sp.* | [unk] |
|  | Ballerus | *Ballerus ballerus* | Zope |
|  | Barbus | *Barbus barbus* | Barbel |
|  | Blicca | *Blicca bjoerkna* | White bream |
| Cyprinidae | Campostoma | *Campostoma anomalum* | Central stoneroller |
|  |  | *Campostoma oligolepis* | Largescale stoneroller |
|  | Carassius | *Carassius auratus* | Goldfish |
|  | Chondrostoma | *Chondrostoma nasus* | Common nase |
|  | Chrosomus | *Chrosomus erythrogaster* | Southern redbelly dace |
|  | Clinostomus | *Clinostomus funduloides* | Rosyside dace |
|  | Coreius | *Coreius heterodon* | [unk] |
|  | Couesius | *Couesius plumbeus* | Lake chub |
|  | Ctenopharyngodon | *Ctenopharyngodon idella* | Grass carp |
|  | Cyprinella | *Cyprinella analostana* | Satinfin shiner |
|  |  | *Cyprinella callistia* | Alabama shiner |
|  |  | *Cyprinella gibbsi* | Tallapoosa shiner |
|  |  | *Cyprinella lutrensis* | Red shiner |
|  |  | *Cyprinella spiloptera* | Spotfin shiner |
|  |  | *Cyprinella venusta* | Blacktail shiner |
|  |  | *Cyprinella whipplei* | Steelcolor shiner |
|  | Cyprinus | *Cyprinus carpio* | Common carp |
|  | Erimystax | *Erimystax dissimilis* | Streamline chub |
|  |  | *Erimystax x-punctatus* | Gravel chub |
|  | Exoglossum | *Exoglossum maxillingua* | Cutlips minnow |
|  | Gila | *Gila atraria* | Utah chub |
|  |  | *Gila cypha* | Humpback chub |
|  |  | *Gila robusta* | Roundtail chub |
|  | Gobio | *Gobio gobio* | Gudgeon |
|  | Hesperoleucus | *Hesperoleucus symmetricus* | California roach |
|  | Hybognathus | *Hybognathus hayi* | Cypress minnow |
|  |  | *Hybognathus regius* | Eastern silvery minnow |
|  | Hybopsis | *Hybopsis winchelli* | Clear chub |
|  | Hypophthalmichthys | *Hypophthalmichthys molitrix* | Silver carp |
|  |  | *Hypophthalmichthys nobilis* | Bighead carp |
|  | Leuciscus | *Leuciscus aspius* | Asp |
|  |  | *Leuciscus idus* | Ide |
|  |  | *Leuciscus leuciscus* | Common dace |
|  | Luxilus | *Luxilus chrysocephalus* | Striped shiner |
|  |  | *Luxilus cornutus* | Common shiner |
|  |  | *Luxilus zonatus* | Bleeding shiner |
|  | Lythrurus | *Lythrurus bellus* | Pretty shiner |
|  |  | *Lythrurus fumeus* | Ribbon shiner |
|  |  | *Lythrurus umbratilis* | Redfin shiner |
|  | Macrhybopsis | *Macrhybopsis aestivalis* | Speckled chub |
|  |  | *Macrhybopsis storeriana* | Silver chub |
|  | Mylocheilus | *Mylocheilus caurinus* | Peamouth |
|  | Mylopharodon | *Mylopharodon conocephalus* | Hardhead |
|  | Mylopharyngodon | *Mylopharyngodon piceus* | Black carp |
|  | Nocomis | *Nocomis biguttatus* | Hornyhead chub |
|  |  | *Nocomis leptocephalus* | Bluehead chub |
|  |  | *Nocomis micropogon* | River chub |
|  | Notemigonus | *Notemigonus crysoleucas* | Golden shiner |
|  | Notropis | *Notropis ammophilus* | Orangefin shiner |
|  |  | *Notropis amoenus* | Comely shiner |
|  |  | *Notropis atherinoides* | Emerald shiner |
|  |  | *Notropis baileyi* | Rough shiner |
| Cyprinidae | Notropis | *Notropis boops* | Bigeye shiner |
|  |  | *Notropis buccatus* | Silverjaw minnow |
|  |  | *Notropis buchanani* | Ghost shiner |
|  |  | *Notropis candidus* | Silverside shiner |
|  |  | *Notropis edwardraneyi* | Fluvial shiner |
|  |  | *Notropis greenei* | Wedgespot shiner |
|  |  | *Notropis hudsonius* | Spottail shiner |
|  |  | *Notropis nubilus* | Ozark minnow |
|  |  | *Notropis percobromus* | Carmine shiner |
|  |  | *Notropis procne* | Swallowtail shiner |
|  |  | *Notropis rubellus* | Rosyface shiner |
|  |  | *Notropis stilbius* | Silverstripe shiner |
|  |  | *Notropis stramineus* | Sand shiner |
|  |  | *Notropis texanus* | Weed shiner |
|  |  | *Notropis uranoscopus* | Skygazer shiner |
|  |  | *Notropis volucellus* | Mimic shiner |
|  | Opsopoeodus | *Opsopoeodus emiliae* | Pugnose minnow |
|  | Parachondrostoma | *Parachondrostoma toxostoma* | [unk] |
|  | Phenacobius | *Phenacobius catostomus* | Riffle minnow |
|  | Phoxinus | *Phoxinus phoxinus* | Eurasian minnow |
|  | Pimephales | *Pimephales notatus* | Bluntnose minnow |
|  |  | *Pimephales promelas* | Fathead minnow |
|  |  | *Pimephales vigilax* | Bullhead minnow |
|  | Platygobio | *Platygobio gracilis* | Flathead chub |
|  | Ptychocheilus | *Ptychocheilus grandis* | Sacramento pikeminnow |
|  |  | *Ptychocheilus lucius* | Colorado pikeminnow |
|  |  | *Ptychocheilus oregonensis* | Northern pikeminnow |
|  | Rhinichthys | *Rhinichthys atratulus* | Blacknose dace |
|  |  | *Rhinichthys cataractae* | Longnose dace |
|  |  | *Rhinichthys osculus* | Speckled dace |
|  |  | *Rhinichthys umatilla* | Umatilla Dace |
|  | Rhodeus | *Rhodeus amarus* | European bitterling |
|  |  | *Rhodeus sericeus* | Bitterling |
|  | Richardsonius | *Richardsonius balteatus* | Redside shiner |
|  | Romanogobio | *Romanogobio albipinnatus* | White-finned gudgeon |
|  | Rutilus | *Rutilus rutilus* | Roach |
|  | Scardinius | *Scardinius erythrophthalmus* | Rudd |
|  | Schizothorax | *Schizothorax plagiostomus* | [unk] |
|  | Semotilus | *Semotilus atromaculatus* | Creek chub |
|  |  | *Semotilus corporalis* | Fallfish |
|  | Squalius | *Squalius cephalus* | Chub |
|  | Telestes | *Telestes souffia* | Vairone |
|  | Tinca | *Tinca tinca* | Tench |
|  | Vimba | *Vimba vimba* | Vimba bream |
| Diplomystidae | Diplomystes | *Diplomystes nahuelbutaensis* | [unk] |
| Elassomatidae | Elassoma | *Elassoma zonatum* | Banded pygmy sunfish |
| Eleotridae | Gobiomorphus | *Gobiomorphus australis* | Striped gudgeon |
|  |  | *Gobiomorphus coxii* | Cox’s gudgeon |
|  | Hypseleotris | *Hypseleotris compressa* | Empire gudgeon |
|  |  | *Hypseleotris kluzingeri* | Western carp gudgeon |
|  | Philypnodon | *Philypnodon grandiceps* | Flat-headed gudgeon |
| Embiotocidae | Hysterocarpus | *Hysterocarpus traskii* | Russian river tule perch |
| Erythrinidae | Hoplias | *Hoplias sp.* | [unk] |
| Esocidae | Esox | *Esox americanus* | Redfin pickerel |
|  |  | *Esox lucius* | Northern pike |
|  |  | *Esox niger* | Chain pickerel |
| Fundulidae | Fundulus | *Fundulus catenatus* | Northern studfish |
|  |  | *Fundulus diaphanus* | Banded killifish |
|  |  | *Fundulus olivaceus* | Blackspotted topminnow |
|  |  | *Fundulus zebrinus* | Plains killifish |
| Galaxiidae | Galaxias | *Galaxias brevipinnis* | Koaro |
|  |  | *Galaxias maculatus* | Inanga |
|  |  | *Galaxias olidus* | Mountain galaxias |
| Gasterosteidae | Culaea | *Culaea inconstans* | Brook stickleback |
|  | Gasterosteus | *Gasterosteus aculeatus* | Three-spined stickleback |
| Gobiidae | Ponticola | *Ponticola kessleri* | Bighead goby |
|  | Proterorhinus | *Proterorhinus marmoratus* | Tubenose goby |
| Gymnotidae | Gymnotus | *Gymnotus carapo* | Banded knifefish |
| Heptapteridae | Pimelodella | *Pimelodella sp.* | [unk] |
|  | Rhamdia | *Rhamdia quelen* | South American catfish |
| Hiodontidae | Hiodon | *Hiodon alosoides* | Goldeye |
|  |  | *Hiodon tergisus* | Mooneye |
| Ictaluridae | Ameiurus | *Ameiurus catus* | White catfish |
|  |  | *Ameiurus melas* | Black bullhead |
|  |  | *Ameiurus natalis* | Yellow bullhead |
|  |  | *Ameiurus nebulosus* | Brown bullhead |
|  |  | *Ameiurus platycephalus* | Flat bullhead |
|  | Ictalurus | *Ictalurus furcatus* | Blue catfish |
|  |  | *Ictalurus punctatus* | Channel catfish |
|  | Noturus | *Noturus eleutherus* | Mountain madtom |
|  |  | *Noturus exilis* | Slender madtom |
|  |  | *Noturus flavus* | Stonecat |
|  |  | *Noturus funebris* | Black madtom |
|  |  | *Noturus gyrinus* | Tadpole madtom |
|  |  | *Noturus insignis* | Margined madtom |
|  |  | *Noturus lachneri* | Ouachita madtom |
|  |  | *Noturus leptacanthus* | Speckled madtom |
|  |  | *Noturus nocturnus* | Freckled madtom |
|  | Pylodictis | *Pylodictis olivaris* | Flathead catfish |
| Lepisosteidae | Lepisosteus | *Lepisosteus oculatus* | Spotted gar |
|  |  | *Lepisosteus osseus* | Longnose gar |
|  |  | *Lepisosteus platostomus* | Shortnose gar |
| Loricariidae | Hypostomus | *Hypostomus sp.* | [unk] |
| Lotidae | Lota | *Lota lota* | Burbot |
| Mordaciidae | Mordacia | *Mordacia mordax* | Shorthead lamprey |
| Moronidae | Morone | *Morone americana* | White perch |
| Moronidae | Morone | *Morone chrysops* | White bass |
|  |  | *Morone chrysops x Morone saxitilis* | [unk] |
|  |  | *Morone saxatilis* | Striped bass |
| Mugilidae | Mugil | *Mugil cephalus* | Flathead grey mullet |
|  | Trachystoma | *Trachystoma petardi* | Pinkeye mullet |
| Nemacheilidae | Barbatula | *Barbatula barbatula* | Stone loach |
|  | Triplophysa | *Triplophysa marmorata* | [unk] |
| Osmeridae | Osmerus | *Osmerus mordax* | Rainbow smelt |
| Paralichthyidae | Paralichthys | *Paralichthys lethostigma* | Southern flounder |
| Parodontidae | Apareiodon | *Apareiodon affinis* | Darter characine |
| Percichthyidae | Macquaria | *Macquaria australasica* | Macquarie perch |
|  |  | *Macquaria colonorum* | Estuary perch |
|  |  | *Macquaria novemaculeata* | Australian bass |
| Percidae | Ammocrypta | *Ammocrypta beanii* | Naked sand darter |
|  |  | *Ammocrypta meridiana* | Southern sand darter |
|  | Crystallaria | *Crystallaria asprella* | Crystal darter |
|  | Etheostoma | *Etheostoma blennioides* | Greenside darter |
|  |  | *Etheostoma caeruleum* | Rainbow darter |
|  |  | *Etheostoma chuckwachatte* | Lipstick darter |
|  |  | *Etheostoma collettei* | Creole darter |
|  |  | *Etheostoma flabellare* | Fantail darter |
|  |  | *Etheostoma gracile* | Slough darter |
|  |  | *Etheostoma histrio* | Harlequin darter |
|  |  | *Etheostoma nigrum* | Johnny darter |
|  |  | *Etheostoma olmstedi* | Tessellated darter |
|  |  | *Etheostoma proeliare* | Cypress darter |
|  |  | *Etheostoma radiosum* | Orangebelly darter |
|  |  | *Etheostoma rupestre* | Rock darter |
|  |  | *Etheostoma spectabile* | Orangethroat darter |
|  |  | *Etheostoma stigmaeum* | Speckled darter |
|  |  | *Etheostoma tallapoosae* | Tallapoosa darter |
|  |  | *Etheostoma tetrazonum* | Missouri saddled darter |
|  |  | *Etheostoma variatum* | Variegate darter |
|  |  | *Etheostoma whipplei* | Redfin darter |
|  |  | *Etheostoma zonale* | Banded darter |
|  | Gymnocephalus | *Gymnocephalus baloni* | Danube ruffe |
|  |  | *Gymnocephalus cernua* | Ruffe |
|  | Perca | *Perca flavescens* | American yellow perch |
|  |  | *Perca fluviatilis* | European perch |
|  | Percina | *Percina bimaculata* | Chesapeake logperch |
|  |  | *Percina caprodes* | Logperch |
|  |  | *Percina copelandi* | Channel darter |
|  |  | *Percina cymatotaenia* | Bluestripe darter |
|  |  | *Percina fulvitaenia* | Ozark logperch |
|  |  | *Percina kathae* | Mobile logperch |
|  |  | *Percina lenticula* | Freckled darter |
|  |  | *Percina maculata* | Blackside darter |
|  |  | *Percina nasuta* | Longnose darter |
|  |  | *Percina nigrofasciata* | Blackbanded darter |
|  |  | *Percina palmaris* | Bronze darter |
| Percidae | Percina | *Percina peltata* | Shield darter |
|  |  | *Percina phoxocephala* | Slenderhead darter |
|  |  | *Percina roanoka* | Roanoke darter |
|  |  | *Percina sciera* | Dusky darter |
|  |  | *Percina shumardi* | River darter |
|  |  | *Percina smithvanizi* | Muscadine Darter |
|  |  | *Percina uranidea* | Stargazing darter |
|  |  | *Percina vigil* | Saddleback darter |
|  | Sander | *Sander canadensis* | Sauger |
|  |  | *Sander lucioperca* | Pike-perch |
| Percidae | Sander | *Sander vitreus* | Walleye |
| Perciliidae | Percilia | *Percilia irwini* | [unk] |
| Percopsidae | Percopsis | *Percopsis omiscomaycus* | Trout-perch |
| Petromyzontidae | Entosphenus | *Entosphenus lethophagus* | Pit-Klamath brook lamprey |
|  | Eudontomyzon | *Eudontomyzon mariae* | Ukrainian brook lamprey |
|  | Ichthyomyzon | *Ichthyomyzon castaneus* | Chestnut lamprey |
|  |  | *Ichthyomyzon fossor* | Northern brook lamprey |
|  |  | *Ichthyomyzon gagei* | Southern brook lamprey |
|  | Lampetra | *Lampetra planeri* | European brook lamprey |
|  |  | *Lampetra richardsoni* | Western brook lamprey |
|  | Petromyzon | *Petromyzon marinus* | Sea lamprey |
| Pimelodidae | Parapimelodus | *Parapimelodus valenciennis* | [unk] |
|  | Pimelodus | *Pimelodus absconditus* | [unk] |
|  |  | *Pimelodus atrobrunneus* | [unk] |
|  |  | *Pimelodus maculatus* | [unk] |
| Plotosidae | Tandanus | *Tandanus tandanus* | Freshwater catfish |
| Poeciliidae | Gambusia | *Gambusia affinis* | Mosquitofish |
|  |  | *Gambusia holbrooki* | Eastern mosquitofish |
| Polyodontidae | Polyodon | *Polyodon spathula* | Mississippi paddlefish |
| Pseudomugilidae | Pseudomugil | *Pseudomugil signifer* | Pacific blue eye |
| Retropinnidae | Prototroctes | *Prototroctes maraena* | Australian grayling |
|  | Retropinna | *Retropinna semoni* | Australian smelt |
| Salmonidae | Coregonus | *Coregonus artedi* | Cisco |
|  |  | *Coregonus clupeaformis* | Lake whitefish |
|  | Hucho | *Hucho hucho* | Huchen |
|  | Oncorhynchus | *Oncorhynchus clarkii* | Cutthroat trout |
|  |  | *Oncorhynchus clarkii clarkii* | Coastal Cutthroat Trout |
|  |  | *Oncorhynchus gorbuscha* | Pink salmon |
|  |  | *Oncorhynchus keta* | Chum salmon |
|  |  | *Oncorhynchus kisutch* | Coho salmon |
|  |  | *Oncorhynchus mykiss* | Rainbow trout |
|  |  | *Oncorhynchus nerka* | Sockeye salmon |
|  |  | *Oncorhynchus tshawytscha* | Chinook salmon |
|  | Prosopium | *Prosopium williamsoni* | Mountain whitefish |
|  | Salmo | *Salmo salar* | Atlantic salmon |
|  |  | *Salmo trutta* | Sea trout |
|  | Salvelinus | *Salvelinus confluentus* | Bull trout |
|  |  | *Salvelinus fontinalis* | Brook trout |
|  |  | *Salvelinus leucomaenis* | Whitespotted char |
|  |  | *Salvelinus malma* | Dolly varden |
| Salmonidae | Thymallus | *Thymallus thymallus* | Grayling |
| Sciaenidae | Aplodinotus | *Aplodinotus grunniens* | Freshwater drum |
| Siluridae | Silurus | *Silurus glanis* | Wels catfish |
| Sisoridae | Glyptothorax | *Glyptothorax pectinopterus* | River cat |
| Sparidae | Acanthopagrus | *Acanthopagrus australis* | Yellowfin bream |
| Sternopygidae | Eigenmannia | *Eigenmannia virescens* | Glass knifefish |
| Tetrarogidae | Notesthes | *Notesthes robusta* | Bullrout |
| Trichomycteridae | Trichomycterus | *Trichomycterus areolatus* | [unk] |

### Intervention

A total of 70 hydropower facilities/dams were reported independently (i.e., no more than one dam considered within the study) and of these seven dams/facilities were considered by more than two studies (Fig. S2). Some studies included more than one study design (i.e., *BA* and *DEF_BA*) resulting in more cases per dam than the total number of studies.


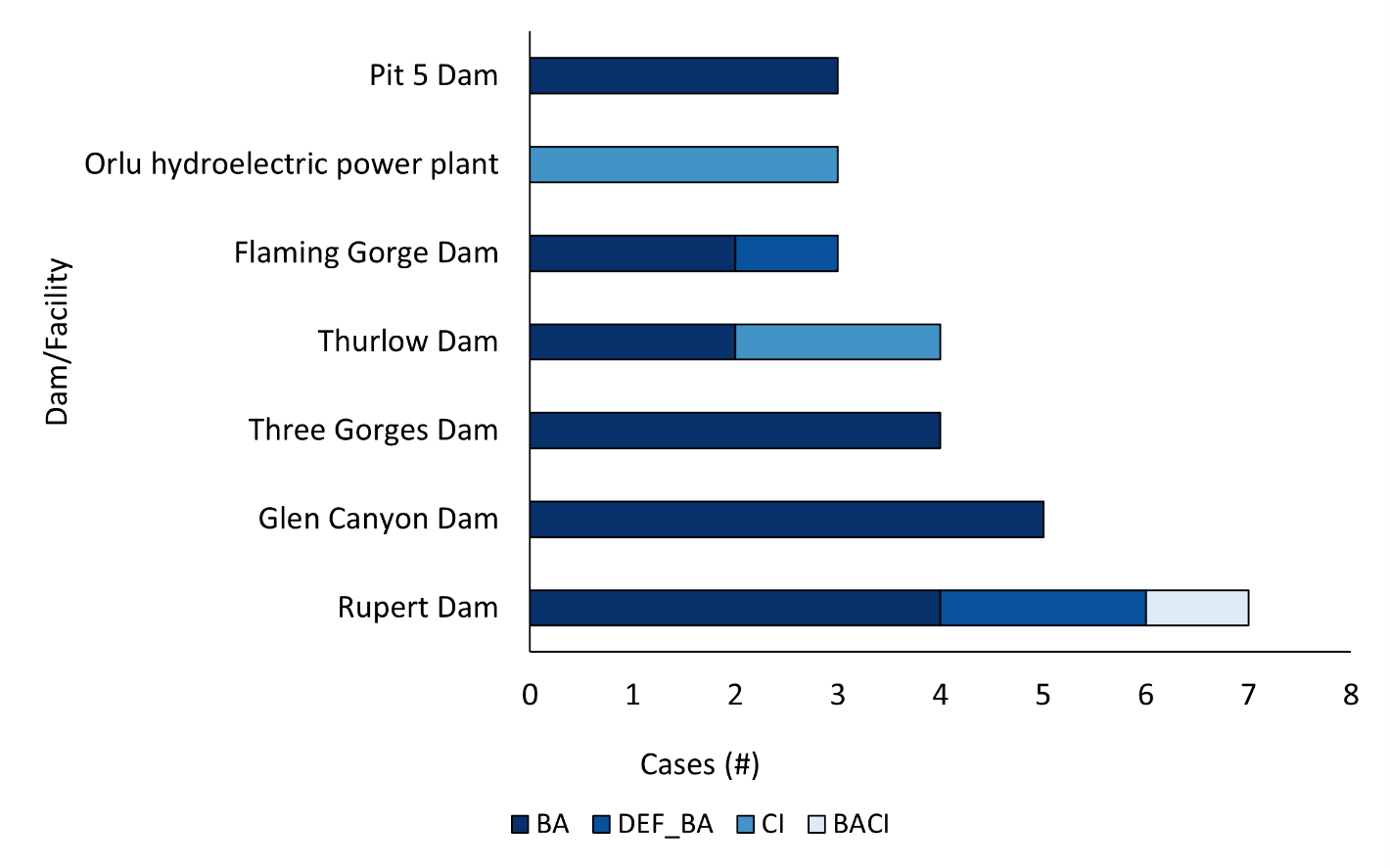


Fig. S2. Dams/facilities considered in more than one study with study design. *BA*: *Before/After*; *BACI*: *Before/After/Control/Impact*; *CI*: *Control/Impact*; and *DEF_BA*: deficient *Before/After* design (i.e., gap of more than five years, or missing information).

### Quantitative synthesis

### Population

For the quantitative synthesis database, five studies considered multiple species combined (i.e., quantitative data was pooled for all species; 12 datasets). All other studies either reported data for individual species (93 studies), or did not report taxonomic information (12 studies). Within datasets with individual species information (226/256), 98 species, from 57 genera and 27 families were evaluated for impacts of flow magnitude alterations. An additional 17 species were identified in multi-species groups but individual effect sizes could not be calculated for these species. The most commonly evaluated species were from the Salmonidae family, including *Oncorhynchus mykiss* (24 datasets), *Salmo trutta* (24 datasets), *Oncorhynchus kisutch* (10 datasets), *Salmo salar* (10 datasets) and *Oncorhynchus tshawytscha* (eight datasets). *Cottus gobio* was also evaluated in eight datasets and all other species were evaluated in fewer than eight datasets each.

### Intervention and outcomes

A total of 32 hydropower facilities/dams were included in the quantitative synthesis database. Of these, seven dams (abundance: 60 datasets; biomass: four datasets) were considered by *CI* studies (Fig. S3), although an additional 26 datasets (abundance: 17 datasets; biomass: nine datasets) were for studies considering multiple dams (i.e., as replicates) or did not report the names of the facilities considered. *BA* studies using within-year replication were conducted at seven dams/facilities (abundance: 37 datasets) (Fig. S3). *BA* studies with interannual replication considered 21 dams/facilities (abundance: 110 datasets; biomass: 17 datasets), although two datasets did not report sufficient information to determine dam/facility name (Fig. S3). Only three dams/facilities (Kinnaird Burn, Cow Green and Glen Canyon Dam) were considered in more than one study design. Of the nine dams/facilities with greater than ten datasets, only three dams were considered in more than one study (Glen Canyon Dam, Rupert Dam, Terzaghi Dam). Of the remaining dams/facilities, only two were considered by more than one study (two studies each).





Fig. S3. Number of individual dams/facilities considered in each analysis type (i.e., *CI* or *BA*) and outcome (abundance and biomass). Numbers in columns indicate the number of datasets and studies (within brackets). Note that three dams/facilities were considered in more than one analysis (i.e., both in a Within-year and Interannual *BA*). An additional 28 datasets considered more than one dam/facility or did not provide sufficient information on location. *CI*: *Control/Impact*; *BA*: *Before/After*.

Most datasets that described an alteration in flow magnitude evaluated the impact of increases in flow magnitude (40%) (Table S2). Of these, 38 datasets considered increases in base flow or increases to at least two flow magnitude elements (e.g., peak flow, base flow, average discharge or short-term variation; 48 datasets). The remaining datasets reported increases in average discharge (3 datasets) or short-term variation (4 datasets). Of the 61 datasets that reported decreases in flow magnitude, 37 reported alterations in average discharge, six reported changes in short-term variation, and one reported alterations to base flow. The remaining 17 datasets reported decreases in two or more flow magnitude elements. A minority of datasets reported both increases and decreases to flow magnitude elements (three datasets) and 90 datasets did not report sufficient information to assign a flow magnitude or flow element alteration.

Table S2. The number of datasets for the two different outcomes by flow magnitude element alterations (refer to Table 4 for definitions). *Multiple* (#) indicates that more than one element was altered and the number of elements that were either increased or decreased. *Unclear* indicates that although a change in flow magnitude was specified, the element considered was not clear, while *Unspecified* indicates that an alteration in flow magnitude was assumed due to the presence of a hydropower facility.

|  | **Abundance** | **Biomass** | **Total** |
| --- | --- | --- | --- |
| Peak Flow | 9 |  | 9 |
| Base Flow | 29 | 10 | 39 |
| Average Discharge | 30 | 10 | 40 |
| Short-term Variation | 8 | 2 | 10 |
| Multiple (2) | 52 | 1 | 53 |
| Multiple (3) | 13 | 1 | 14 |
| Multiple (4) | 1 |  | 1 |
| Unclear | 9 |  | 9 |
| Unspecified | 75 | 6 | 81 |
| **Total** | **226** | **30** | **256** |

Of datasets that reported life stage (133/268), 29% reported more than one life stage but combined quantitative results for life stages together (i.e., mixed life stages). Age-0 fish were the most frequently reported single age group (11%), followed by adults (5%), larvae (3%), number of eggs and juveniles (1% each). Half of the datasets did not report the life stage of the studied fish.

Gear based sampling was the most common sampling method used (85%), with all other sampling methods accounting for the remaining 15% (visual: 4%, other: 2%, angling: <1%, multiple 9% of datasets). Sampling occurred in all seasons, with 52% of datasets having sampling in more than one season, normally summer and fall (21%). When samples occurred in a single season, the most common was fall (25%) followed by summer (15%) and spring (5%). Fall was sampled in 193 of all datasets, summer in 173, spring in 93 and winter in only 30 datasets (but never without some other season also being sampled). Because more than 50% of datasets included more than one season, the total number of datasets evaluating seasons exceeds the total number of datasets (256). First sampling often occurred within the first year of the intervention (59% of datasets). Few datasets reported sampling beginning after the first year of the intervention (8% started between two and four years after); however, 12% of datasets reported that sampling began >4 years post-intervention. Additionally, 21% of studies did not report when post-intervention sampling was started.
