# Supplementary material for: How do changes in flow magnitude due to hydropower operations affect fish abundance and biomass in temperate regions? A systematic review
Source: Environ Evid. 2022 Feb 4;11:3. doi: 10.1186/s13750-021-00254-8 (PMC8813579; doi:10.1186/s13750-021-00254-8)
Supplement: Supplementary file 12 — Additional file 12. Meta-analyses and publication bias. Global meta-analyses, publication bias, sensitivity analyses, and moderator analysis. All forest (i.e., summary plot of all effect size estimates) and funnel (i.e., visual assessment of publication bias using a scatter plot of effect sizes versus a measure of precision) plots from global and sensitivity analyses. [file 13750_2021_254_MOESM12_ESM.docx]

**Additional File 12. Meta-analyses and publication bias**

Description: Global meta-analyses, publication bias, sensitivity analyses, and moderator analysis. All forest (i.e, summary plot of all effect size estimates) and funnel (i.e., visual assessment of publication bias using a scatter plot of effect sizes versus a measure of precision) plots from global and sensitivity analyses.

This space intentionally left blank

**Global meta-analysis – *Control/Impact* Studies**

***Abundance***

*
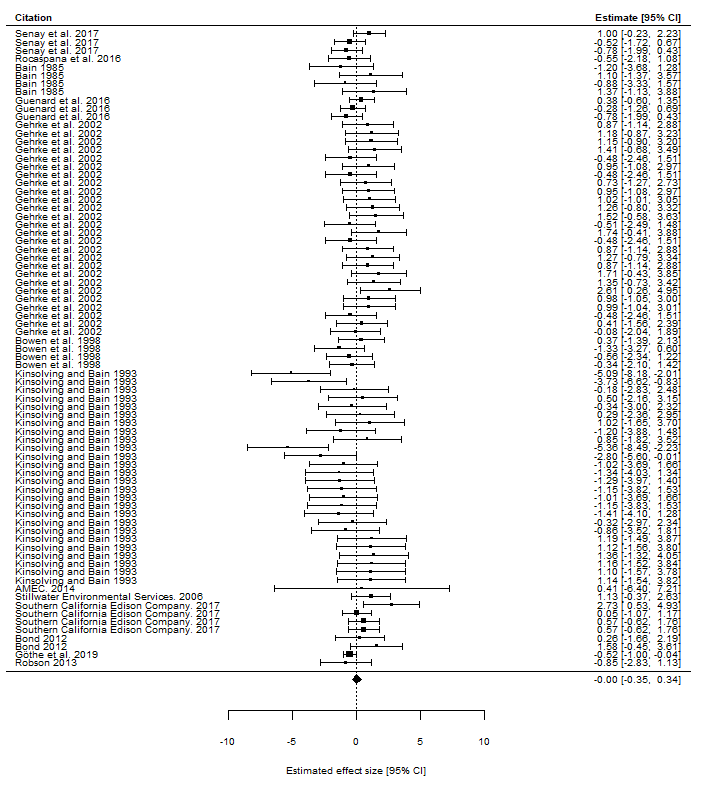
*Fig. S1. Summary plot of all effect size estimates from *Control/Impact* evaluations of the impact of flow magnitude alterations on fish abundance (*k*=77). Error bars indicate 95% confidence intervals. A positive mean value (right of dashed zero line) indicates that the abundance was higher in treatment areas than in comparator areas (no intervention). Diamond: overall mean effect size of random-effects model.

*
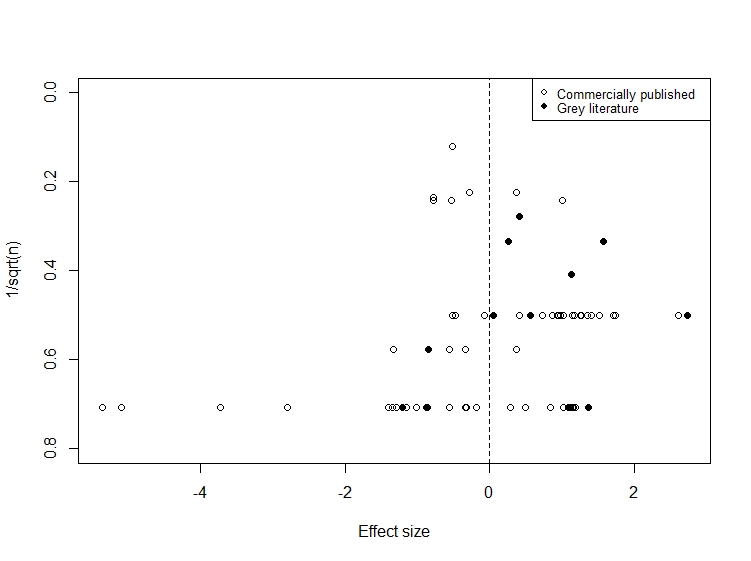
*Fig. S2 Funnel plot showing *Control/Impact* studies of abundance (*k* = 77). Open circles indicate datasets from commercially published articles and filled circles indicate datasets from grey literature. Summary effect is indicated by the dashed line.

The Cook’s distance plot (Fig. S3) indicates a few influential effect sizes, with one outlier of concern (i.e., a single dataset with a comparatively large sample size relative to the other effect sizes). However, removing this single effect size from the random effects model yielded a similar result to the meta-analysis using all datasets, albeit a larger, positive overall effect size trend [Hedge’s *g* = 0.06 (95% CI -0.30, 0.42; *k* = 76, *p=* 0.745)] (see forest plot Fig S4) and had no impact on funnel plot asymmetry (see Fig S5) or the failsafe number. 
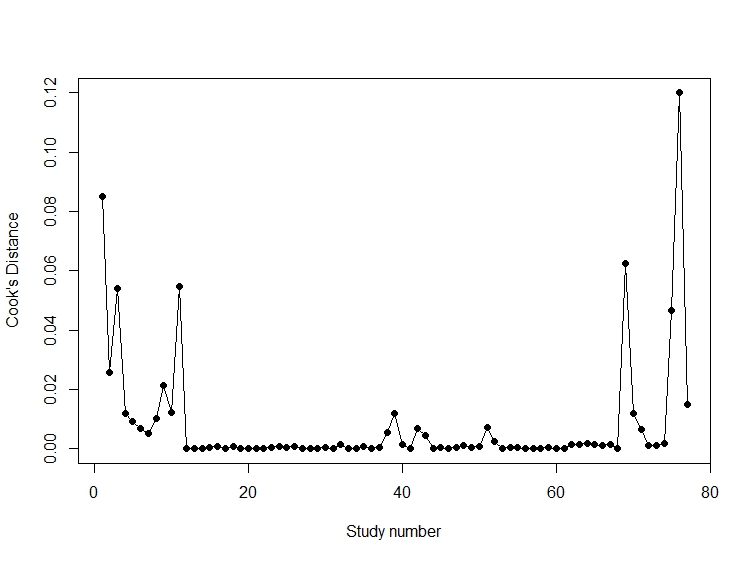


Fig. S3. Cook’s distance plot indicating influence of effect size. Note the outlier of concern (Cook’s distance ≈ 0.12).


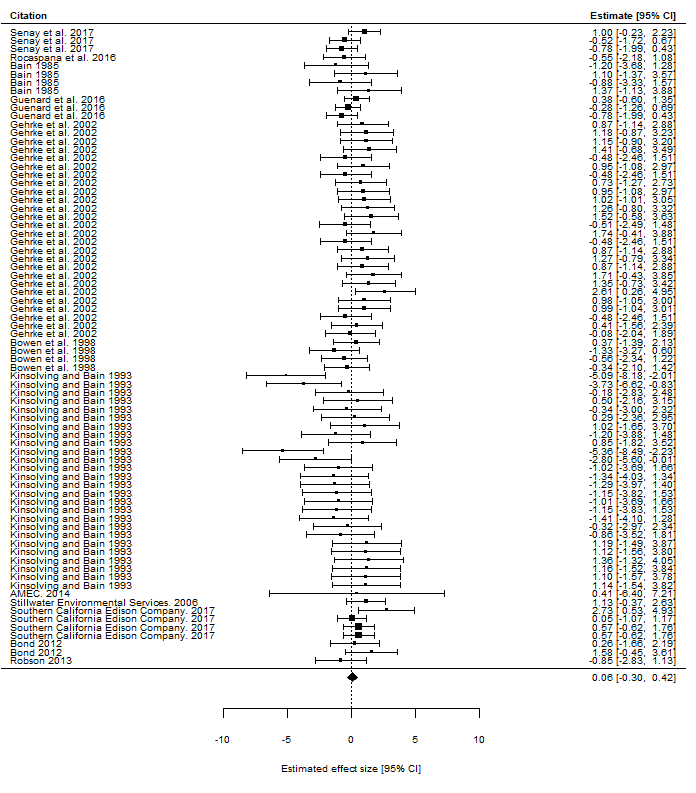


Fig. S4. Summary plot of all effect size estimates from *Control/Impact* evaluations of the impact of flow magnitude alterations on fish abundance, after removing one outlier of concern with a comparatively large sample size relative to the other effect sizes (*k*=76). Error bars indicate 95% confidence intervals. A positive mean value (right of dashed zero line) indicates that the abundance was higher in treatment areas than in comparator areas (no intervention). Diamond: overall mean effect size of random-effects model.


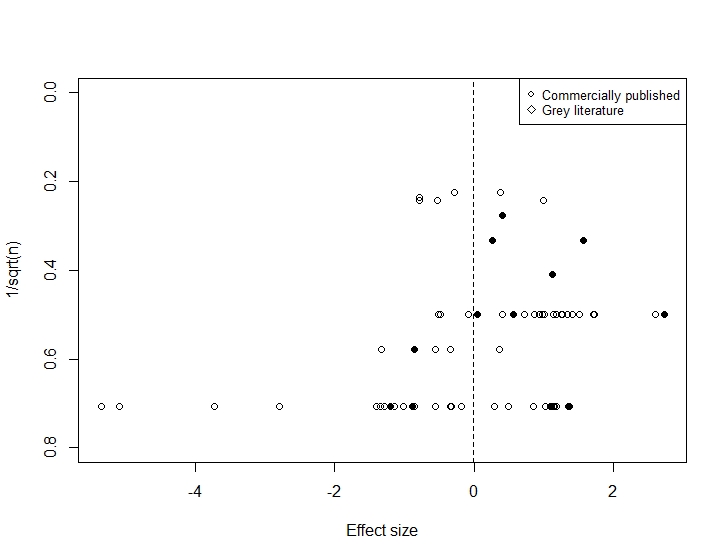
 Fig. S5 Funnel plot showing *Control/Impact* studies of abundance (*k* = 76), with the one influential outlier removed. Open circles indicated datasets from commercially published articles and filled circles indicate datasets from grey literature. Summary effect is indicated by the dashed line.

The sensitivity analyses for both medium validity studies and studies with true replication showed a more negative effect of flow magnitude changes on fish abundance compared to the overall meta-analysis. The difference in the relative magnitude of effect sizes for these analyses suggests that the results may not be fully robust to the inclusion of studies with low validity or pseudoreplication, but the effect sizes were non-significant (Table S1). Sensitivity analyses of studies where no stocking influenced outcomes indicated a slight increase in the effect size relative to the overall meta-analysis, as did the analyses of studies that specified flow magnitude specifically (i.e., removing studies reporting comparisons of regulated to unregulated systems without a measure of flow magnitude); however, the results are comparable to the overall meta-analysis and the effect sizes are non-significant. This indicates the results may be robust to the inclusion of studies that: (i) may have been impacted by stocking, or (ii) studies that did not specify a flow magnitude (Table S1). The sensitivity analysis based only on studies comparing a single intervention to a single comparator showed a positive effect of flow magnitude change on fish abundance compared to the overall meta-analysis, but the relative magnitude of the effect sizes was comparable. Similarly, the sensitivity analysis for studies that required variance imputation had a comparable effect size to the overall meta-analysis (Table S1). In both cases, the summary effect was non-significant, again indicating that the overall meta-analysis result may be robust to the inclusion of studies comparing multiple interventions with a single comparator, or where imputation was used.

Table S1. Summary statistics of applicable sensitivity analyses for *CI* study designs and abundance. Statistical significance at *p*<0.05.

| **Analysis** | **Standardized mean difference (Hedge’s *g*)** |
| --- | --- |
| Global analysis (*k* = 77) | -0.001 (95% CI -0.35, 0.34; *p* = 0.997) |
| Medium Validity (*k* = 39) | -0.18 (95% CI -0.60, 0.23; *p=* 0.385) |
| Without imputation (*k* = 75) | 0.02 (95% CI -0.34, 0.37; *p* = 0.923) |
| Without stocking (*k* = 66) | -0.03 (95% CI -0.48, 0.43; *p*=0.900) |
| True replication (*k* = 11) | -0.10 (95% CI -0.55, 0.35; *p* = 0.6721) |
| Single comparator/single intervention (*k* = 69) | 0.005 (95% CI -0.47, 0.48; *p* = 0.985) |
| Reported flow magnitude components (*k* = 44) | -0.04 (95% CI -0.47, 0.39; *p* = 0.858) |

Note, a decrease in the abundance of fish from alterations to flow magnitude due to HPP compared to control groups is indicated by a value <0 for Hedges’ *g*. CI: 95% confidence interval. *k*: number of effect sizes.

***Biomass***


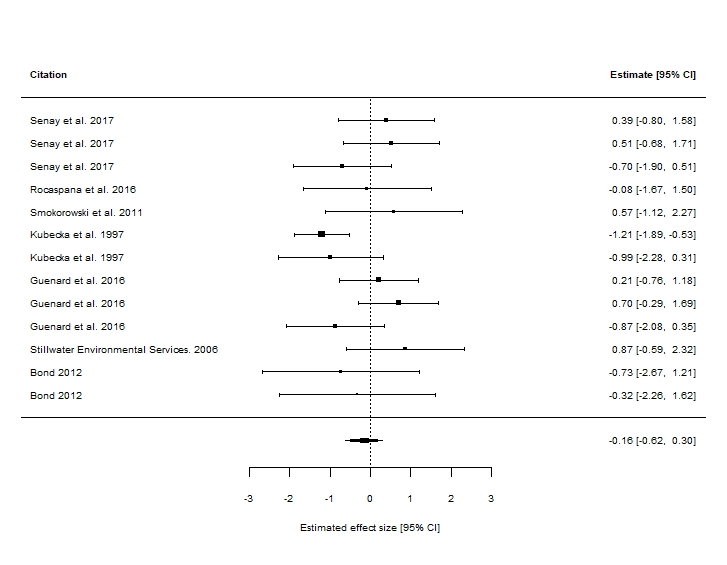


Fig. S6. Summary plot of all effect size estimates from *Control/Impact* evaluations of the impact of flow magnitude alterations on fish biomass (*k*=13). Error bars indicate 95% confidence intervals. A positive mean value (right of dashed zero line) indicates that the abundance was higher in treatment areas than in comparator areas (no intervention). Diamond: overall mean effect size of random-effects model.


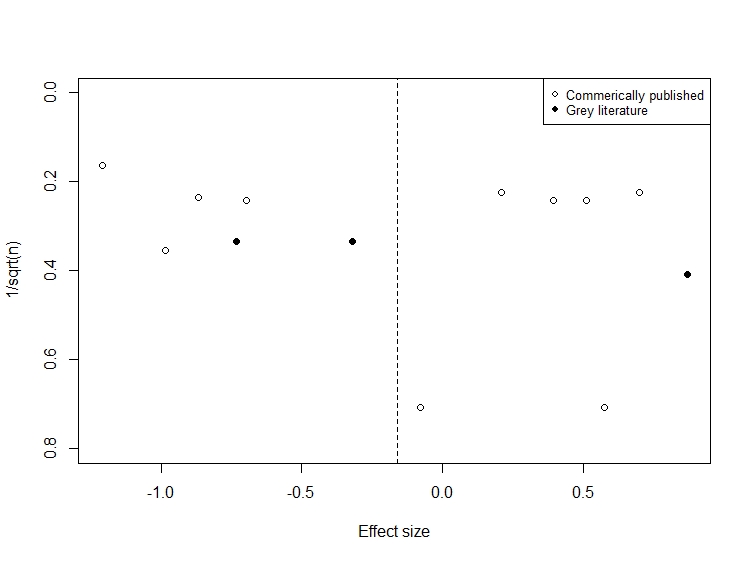


Fig. S7. Funnel plot showing *Control/Impact* studies of biomass (*k* = 13). Open circles indicate datasets from commercially published articles and filled circles indicate datasets from unpublished literature. Summary effect is indicated by the dashed line.

Sensitivity analysis based on only medium validity studies showed a more positive effect of changes to flow magnitude on fish biomass compared to the overall meta-analysis (Table S2). The summary effect was not significant, suggesting that the result of the overall meta-analysis may be robust to differences in study validity. All other sensitivity analyses for biomass and *CI* study designs had slightly larger, more negative effect sizes; however, results were comparable to the overall meta-analysis (Table S2) and no effect sizes were significant. This indicates that results may be robust against the inclusion of studies with imputation, potential impacts of stocking, pseudoreplication, multiple interventions compared to a single comparator, and studies that do not report flow magnitude components (i.e., studies reporting regulated vs unregulated systems without also reporting a flow magnitude component, or reporting multiple flow magnitude components).

Table S2. Summary statistics of applicable sensitivity analyses for *CI* study designs and biomass. Statistical significance at *p*<0.05.

| **Analysis** | **Standardized mean difference (Hedge’s *g*)** |
| --- | --- |
| Global analysis (*k* = 13) | -0.16 (95% CI -0.62, 0.30; *p* = 0.489) |
| Medium Validity (*k* = 7) | 0.07 (95% CI -0.46, 0.61; *p* = 0.789) |
| Without imputation (*k* = 12) | -0.16 (95% CI -0.64, 0.32; *p* = 0.508) |
| Without stocking (*k* = 10) | -0.25 (95% CI -0.79, 0.29; *p* = 0.366) |
| True replication (*k* = 8) | -0.26 (95% CI -0.83, 0.31; *p* = 0.369) |
| Single comparator/single intervention (*k* = 5) | -0.33 (95% CI -1.20, 0.54; *p* = 0.458) |
| Reported flow magnitude components (*k* = 10) | -0.25 (95% CI -0.79, 0.29; *p* = 0.366) |

Note, a decrease in the abundance of fish from alterations to flow magnitude due to HPP compared to control groups is indicated by a value <0 for Hedges’ *g*. CI: 95% confidence interval. *k*: number of effect sizes.

**Global meta-analysis – Within-year *Before/After* studies**

***Abundance***
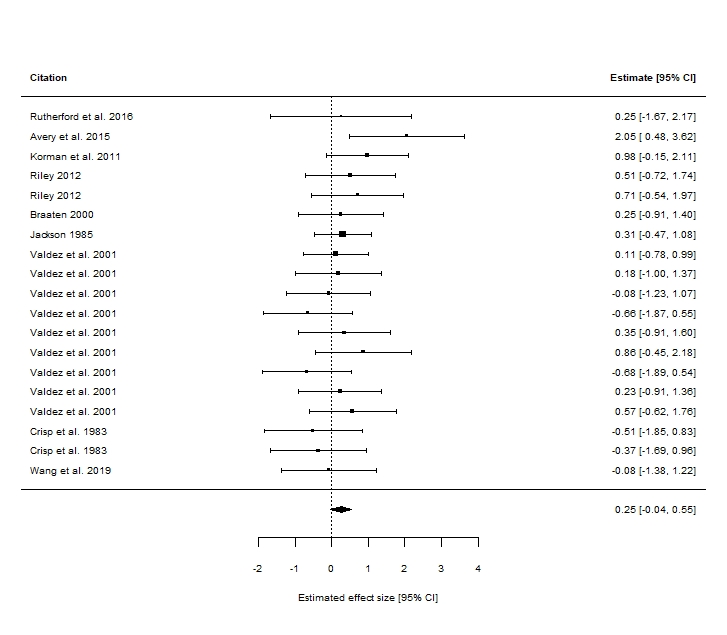


Fig. S8. Summary plot of all effect size estimates from within-year *Before/After* evaluations of the impact of flow magnitude alterations on fish abundance after year 1 (*k*=19). Error bars indicate 95% confidence intervals. A positive mean value (right of dashed zero line) indicates that the abundance was higher in the *After* period than in the *Before* period (no intervention). Diamond: overall mean effect size of random-effects model.


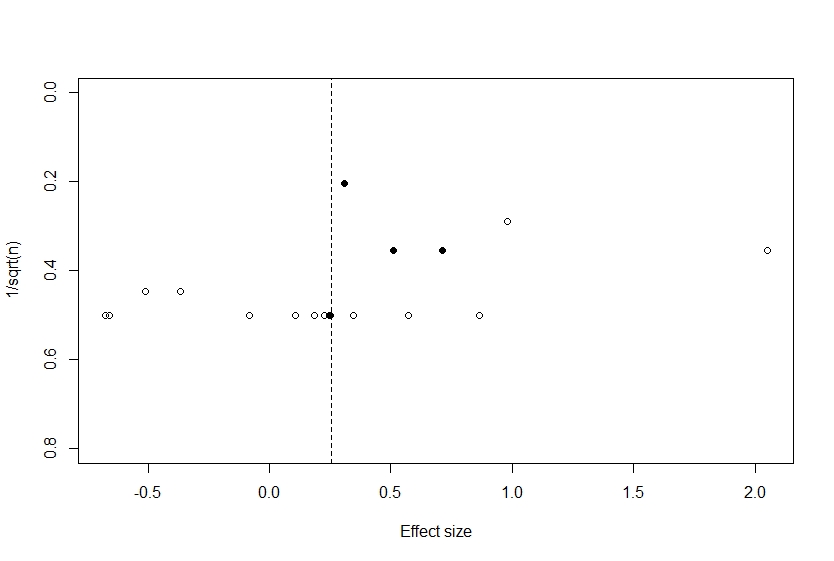


Fig. S9. Funnel plot showing publication bias for within- year *Before/After* studies of abundance (*k* = 19). Open circles indicate datasets from commercially published articles and filled circles indicate datasets from grey literature. Summary effect is indicated by dashed line.


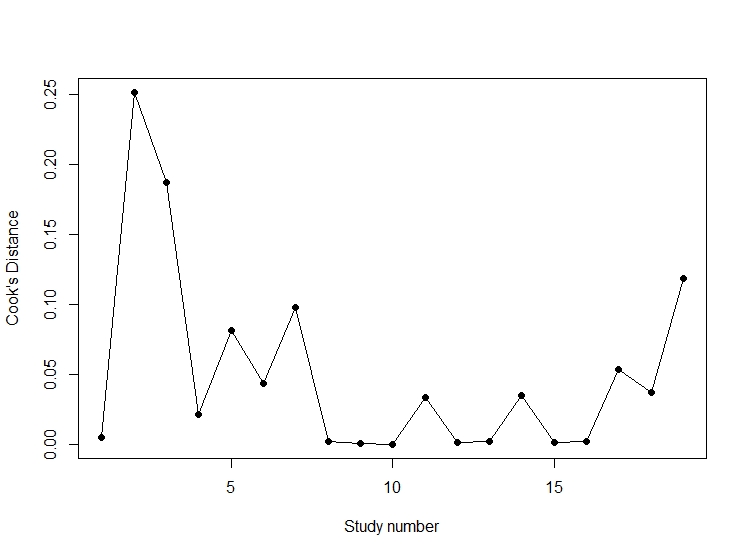


Fig. S10. Cook’s distance plot indicating influence of effect sizes.


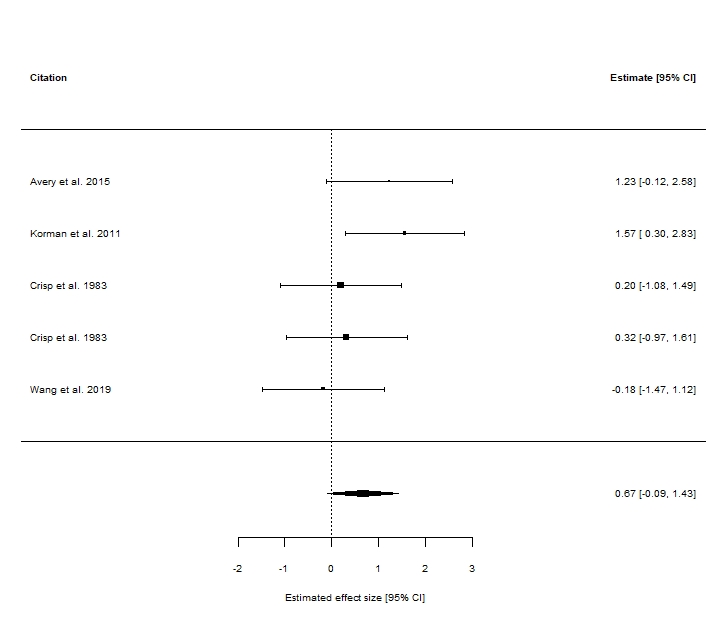


Fig. S11. Summary plot of all effect size estimates from within-year *Before/After* evaluations of the impact of flow magnitude alterations on fish abundance after year 2 (*k*=5). Error bars indicate 95% confidence intervals. A positive mean value (right of dashed zero line) indicates that the abundance was higher in the *After* period than in the *Before* period (no intervention). Diamond: overall mean effect size of random-effects model.


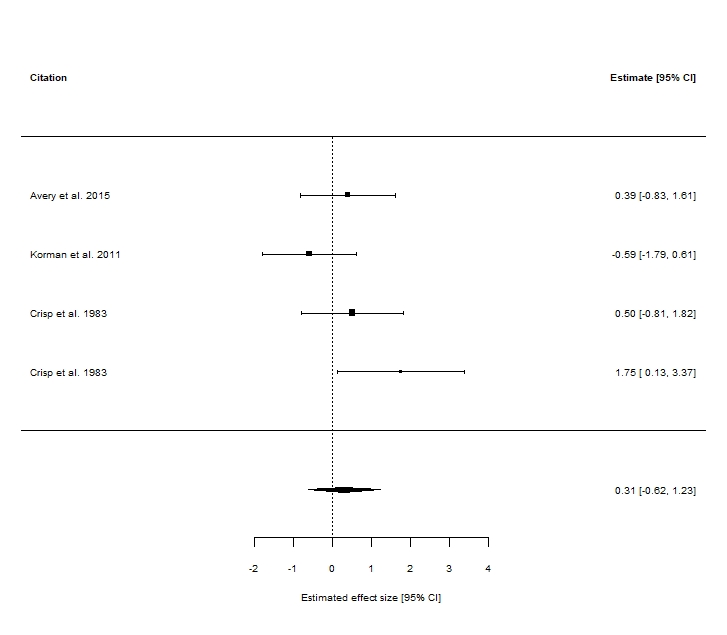


Fig. S12. Summary plot of all effect size estimates from Within-year *Before/After* evaluations of the impact of flow magnitude alterations on fish abundance after year 3 (*k*=4). Error bars indicate 95% confidence intervals. A positive mean value (right of dashed zero line) indicates that the abundance was higher in the *After* period than in the *Before* period (no intervention). Diamond: overall mean effect size of random-effects model.


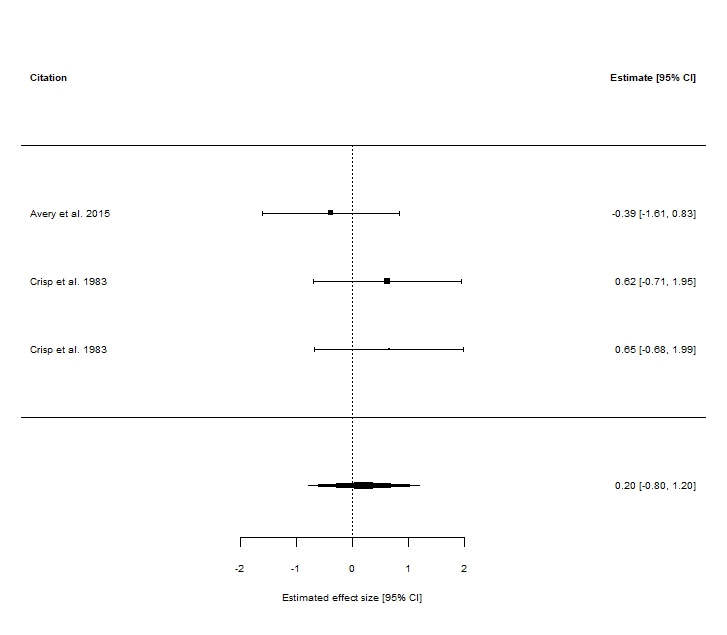


Fig. S13. Summary plot of all effect size estimates from Within-year *Before/After* evaluations of the impact of flow magnitude alterations on fish abundance after year 4 (*k*=3). Error bars indicate 95% confidence intervals. A positive mean value (right of dashed zero line) indicates that the abundance was higher in the *After* period than in the *Before* period (no intervention). Diamond: overall mean effect size of random-effects model.


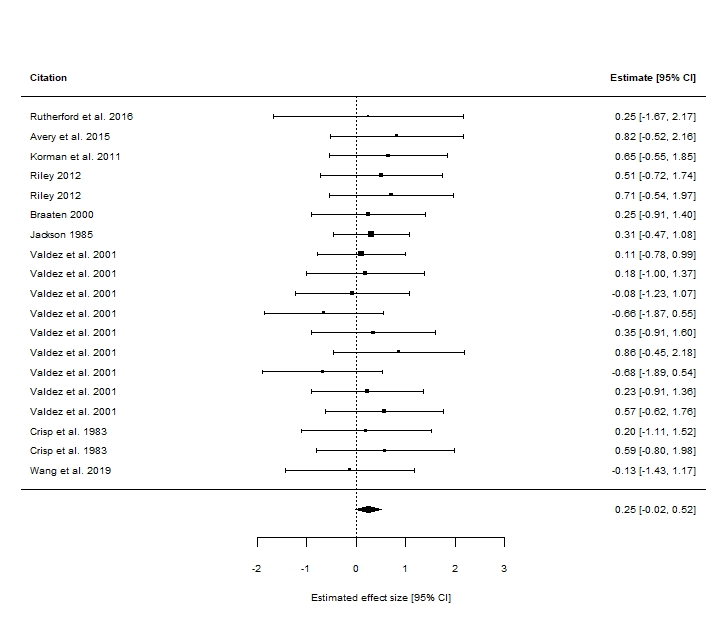


Fig. S14. Summary plot of all effect size estimates from Within-year *Before/After* evaluations of the impact of flow magnitude alterations on fish abundance after year 1- 4 aggregated (*k*=19). Error bars indicate 95% confidence intervals. A positive mean value (right of dashed zero line) indicates that the abundance was higher in the *After* period than in the *Before* period (no intervention). Diamond: overall mean effect size of random-effects model.

***Sensitivity analysis (Abundance, After year 1)***

Sensitivity analyses for within-year *BA* studies were conducted only for year-1 post-intervention datasets. Sensitivity analyses to determine the effect of the inclusion of studies with imputation of missing variances, single comparators and multiple interventions, deficient *BA* or *BACI* studies, and inclusion of outflow regions were not conducted because datasets with these features were not present for within-year *BA* studies. Sensitivity analysis of medium validity studies showed a slightly smaller, but still positive effect size of flow magnitude changes on fish abundance, but this overall response was no longer significant (possibly due to a smaller sample size for medium validity studies only) [Hedges’ *g* = 0.14 (95% CI -0.17, 0.44; *k* = 14, *p* = 0.386)], compared to the overall meta-analysis [Hedges’ *g* = 0.25 (95% CI -0.04, 0.56; *k* = 19, *p* = 0.091)]. The small difference in average effect sizes between the two models suggests that the results may be robust to inclusion of studies with low validity. The sensitivity analysis of studies where stocking clearly did not occur showed a statistically significant and slightly larger, more positive mean effect size [Hedge’s *g* = 0.35 (95% CI 0.02, 0.69; *k* = 17, *p* = 0.040)] than the overall meta-analysis, indicating that the results may not be robust against the inclusion of studies that were potentially impacted by stocking. Similarly, the sensitivity analysis of only studies that reported flow magnitude components showed a statistically significant and slightly larger, more positive mean effect size [Hedge's *g* = 0.35 (95% CI 0.02, 0.69; *k* = 17, *p* = 0.040)] than the overall meta-analysis. However, in both cases the same two effect sizes were removed. Given the small sample size and the removal of two of the six negative effect sizes, it is possible that the response of the model to the removal of these two effect sizes may not be associated with potential stocking or the lack of specific flow magnitude description *per se*, but with some other aspect of the study. It is also important to note that the mean effect sizes for the model with and without potential impacts of stocking, and therefore flow magnitude elements, were within the confidence intervals of the two models (see forest plots with stocking/no reported flow magnitude Fig. S8, without stocking/no reported flow magnitude Fig. S15).


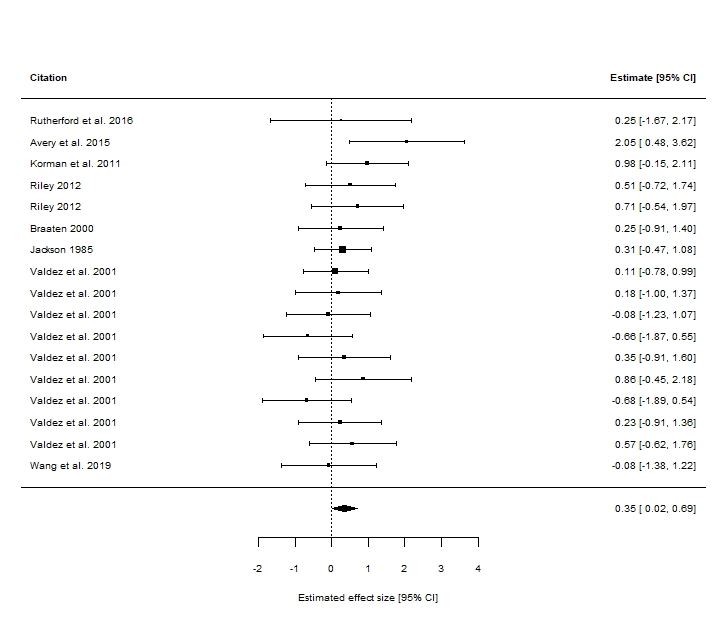


Fig. S15. Summary plot of all effect size estimates from within-year *Before/After* evaluations of the impact of flow magnitude alterations on fish abundance after year 1 (*k*=17), considering only studies where it is clear that no stocking influenced the waterbody during sampling, and flow magnitude components are stated. Error bars indicate 95% confidence intervals. A positive mean value (right of dashed zero line) indicates that the abundance was higher in the *After* period than in the *Before* period (no intervention). Diamond: overall mean effect size of random-effects model. For this analysis, to test the influence of studies where stocking potentially occurred and flow magnitude components are not specified, the same two datasets (which both had these features) were removed and the average effect size for fish abundance was determined with the remaining studies.

**Global meta-analysis – Interannual *Before/After* studies**

***Abundance***


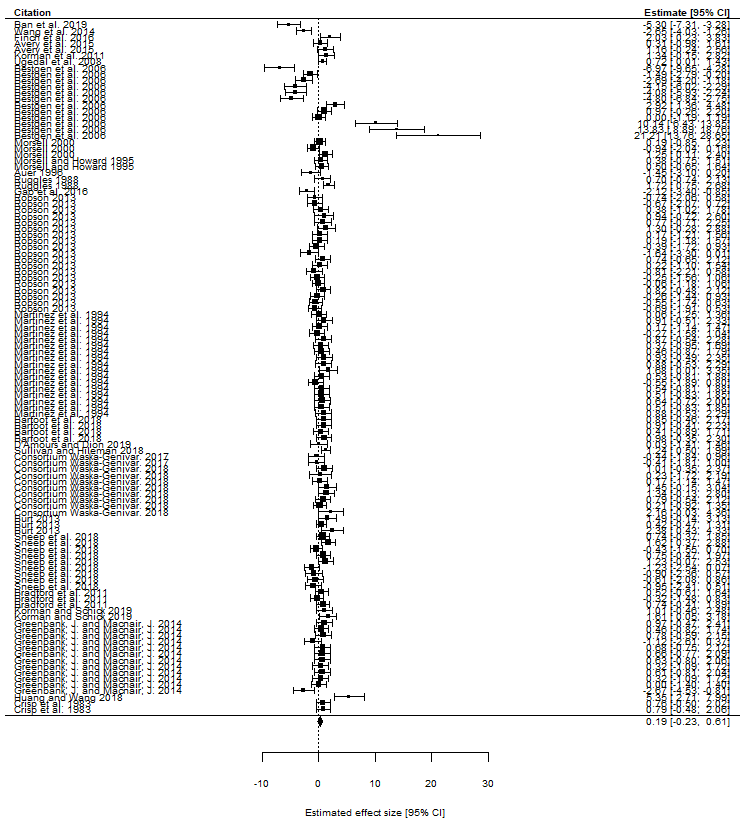


Fig. S16. Summary plot of all effect size estimates from interannual *Before/After* evaluations of the impact of flow magnitude alterations on fish abundance (*k*=112). Datasets from the same study with different interventions were compared to a single comparator. Error bars indicate 95% confidence intervals. A positive mean value (right of dashed zero line) indicates that the abundance was higher in the *After* period than in the *Before* period (no intervention). Diamond: overall mean effect size of random-effects model.


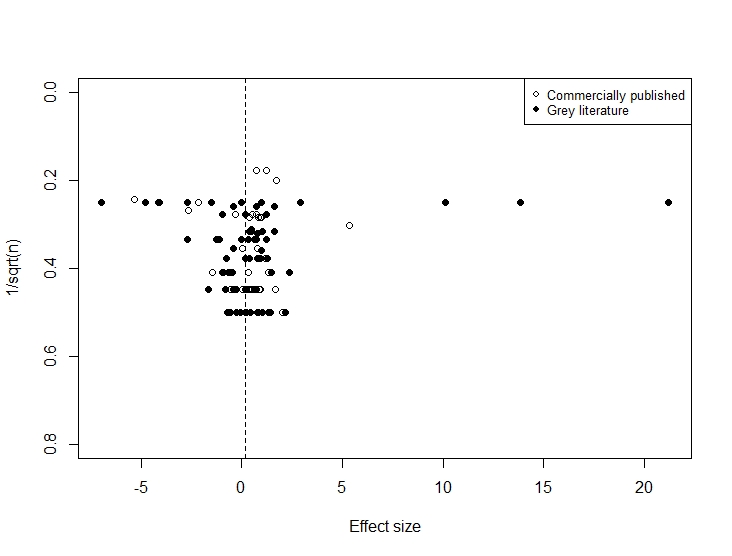


Fig. S17. Funnel plot showing interannual *Before/After* studies of abundance (*k* = 112). Open circles indicate datasets from commercially published articles and filled circles indicate datasets from grey literature. Summary effect is indicated by the dashed line.


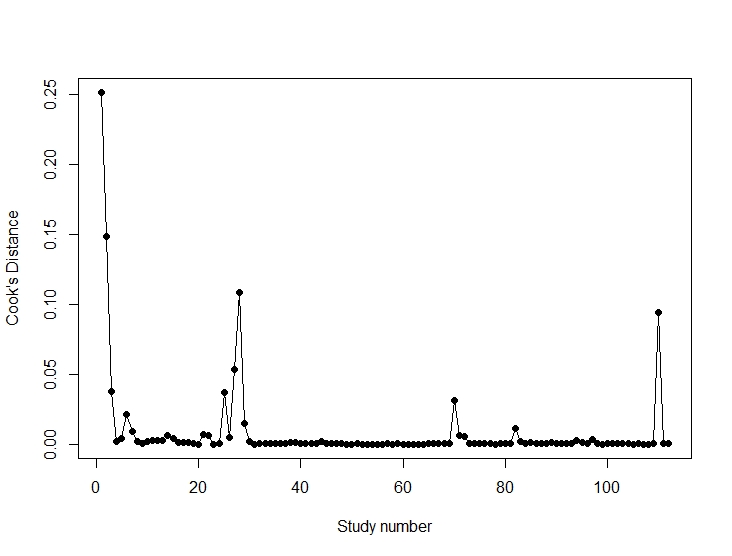


Fig. S18. Cook’s distance plot indicating influence of effect size. Note the outlier of concern (Cook’s distance ≈ 0.25).

For this analysis, we assumed that any study with more than one intervention used the same comparator for all interventions [i.e., if two flow magnitude alterations (trials) are conducted, each can be compared to Trial 0 (the *Before* period), Trial 0 vs. Trial 1 and Trial 0 vs. Trial 2]; however, it is possible that each subsequent intervention could be compared to the previous intervention (i.e., Trial 0 vs. Trial 1, Trial 1 vs. Trial 2). When comparing these two options (i.e., changing the before period for two studies with multiple intervention periods (Fig. S19), using different comparator periods did not greatly alter the overall mean effect size [Hedge's *g* = 0.17 (95% CI -0.25, 0.59; *k* = 112, *p* = 0.4238)] and the direction of effect did not change (see Fig. S20). We conducted all further analysis using the same comparator period.

*
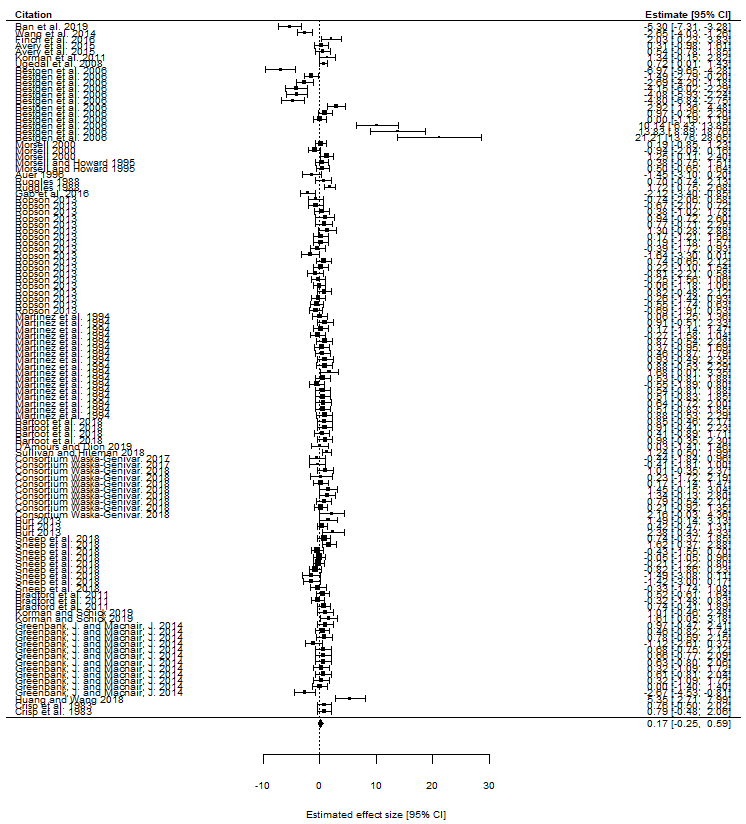
*Fig. S19. Summary plot of all effect size estimates from interannual *Before/After* evaluations of the impact of flow magnitude alterations on fish abundance (*k*=112). Datasets from the same study with different interventions were compared to each previous period (i.e., Trial 2 was compared to Trial 1, rather than the original pre-trial period). Error bars indicate 95% confidence intervals. A positive mean value (right of dashed zero line) indicates that the abundance was higher in the *After* period than in the *Before* period (no intervention). Diamond: overall mean effect size of random-effects model. Diamond: overall mean effect size of random-effects model.


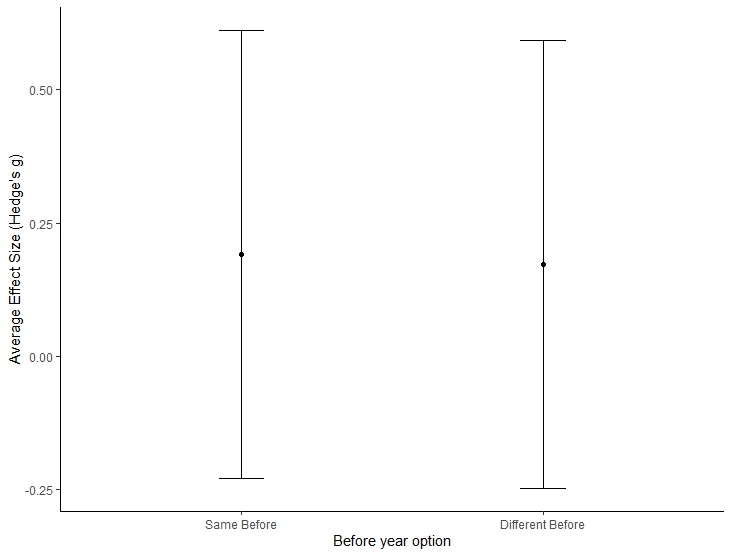


Fig. S20. Comparison of interannual *Before/After* studies’ overall effect size when datasets from studies with more than one intervention are compared to a single pre-trial *Before* period (Same *Before*) or when each subsequent intervention is compared to the previous period (i.e., Trial 2 is compared to Trial 1, rather than to the pre-trial period).

Sensitivity analysis of studies without imputation of missing variance was not possible for interannual *BA* studies because no datasets required these calculations. All sensitivity analyses applicable to interannual *BA* study designs had similar results to the overall meta-analysis (Table S4) indicating that the results of the overall meta-analysis were robust to the inclusion of: (i) studies with low validity; (ii) studies where stocking may have impacted fish outcomes; (iii) studies with multiple interventions and a single comparator; (iv) studies that did not report flow magnitude components (i.e., *BACI* studies that compare before and after the start of regulation, in a regulated and unregulated stream, but do not state what alterations to flow magnitude occurred); (v) studies with deficient *BA* or *BACI* study designs; (vi) studies that averaged yearly averages in the *Before* and *After* periods (Fig S21-S23 for comparisons of the effect sizes for different types of temporal data); and (vii) outfall zones.

Table S4. Summary statistics of applicable sensitivity analyses for interannual *BA* study designs and abundance. Statistical significance at *p*<0.05.

| **Analysis** | **Standardized mean difference (Hedge’s *g*)** |
| --- | --- |
| Global analysis (*k* = 112) | 0.19 (95% CI -0.23, 0.61; *p* = 0.374), |
| Medium Validity (*k* = 40) | 0.11 (95% CI -0.32, 0.54; *p* = 0.602) |
| Without imputation (*k* = 112) | N/A |
| Without stocking (*k* = 70) | 0.19 (95% CI -0.41, 0.79; *p* = 0.535) |
| Single comparator/single intervention (*k* = 101) | 0.17 (95% CI -0.29, 0.64; *p* = 0.464) |
| Reported flow magnitude components (*k* = 82) | 0.09 (95% CI -0.37, 0.56; *p* = 0.694) |
| Without deficient BA or BACI (*k* = 108) | 0.17 (95% CI -0.27, 0.61; *p* = 0.453) |
| Without averages of averages (*k* = 91) | 0.15 (95% CI -0.41, 0.71; *p* = 0.595) |
| Inclusion of outfall zone (*k* = 103) | 0.18 (95% CI -0.27, 0.62; *p* = 0.439) |

Note, a decrease in the abundance of fish from alterations to flow magnitude due to HPP compared to control groups is indicated by a value <0 for Hedges’ *g*. CI: 95% confidence interval. *k*: number of effect sizes. N/A: unable to assess moderator due to insufficient sample size or lack of variation.

*
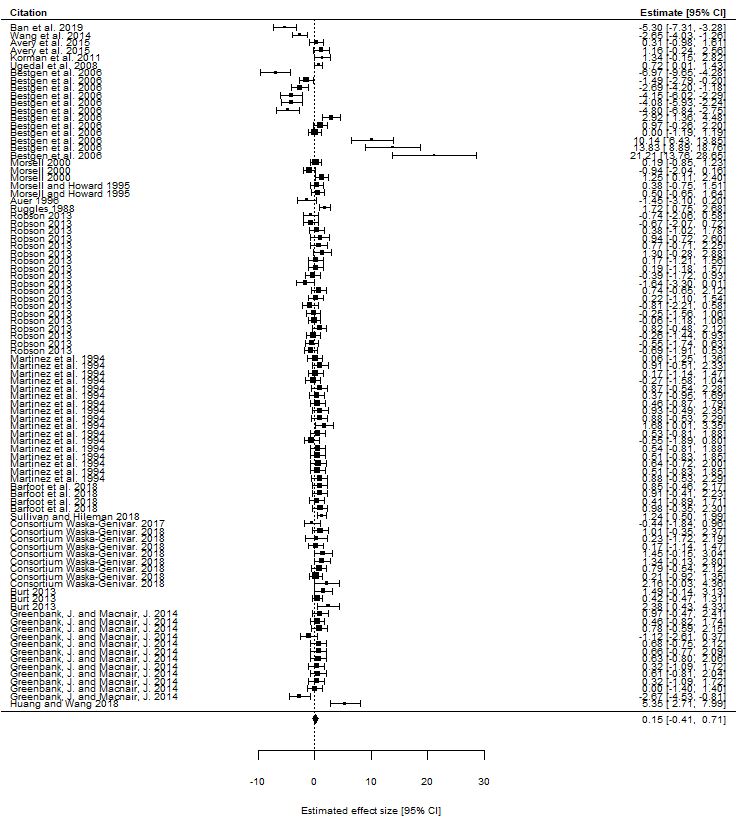
* Fig. S21. Summary plot of all effect size estimates from interannual *Before/After* evaluations of the impact of flow magnitude alterations on fish abundance that reported fish outcomes as either single datapoints per year, or sums per year which were then averaged in the *Before* and *After* periods (*k*=91). Error bars indicate 95% confidence intervals. A positive mean value (right of dashed zero line) indicates that the abundance was higher in the *After* period than in the *Before* period (no intervention). Diamond: overall mean effect size of random-effects model.

*
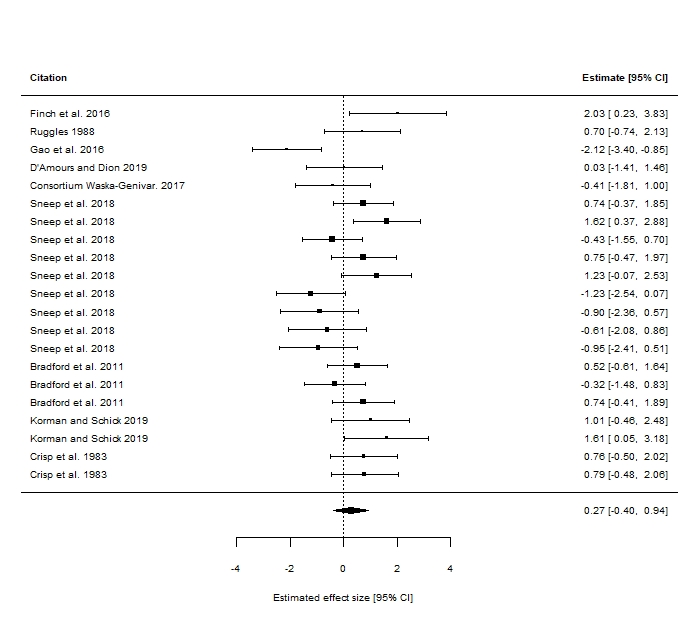
*Fig. S22. Summary plot of all effect size estimates from interannual *Before/After* evaluations of the impact of flow magnitude alterations on fish abundance that reported fish outcomes as averages of averages for the *Before* and *After* periods (*k*=21). Error bars indicate 95% confidence intervals. A positive mean value (right of dashed zero line) indicates that the abundance was higher in the *After* period than in the *Before* period (no intervention). Diamond: overall mean effect size of random-effects model.


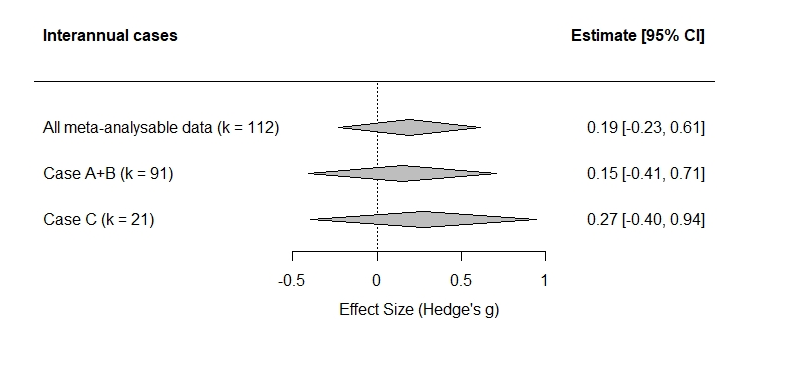


Fig. S23. Comparison of effect sizes for three types of interannual *Before/After* data, for the impact of flow magnitude alterations on fish abundance. *Case A*: fish sampled only once per year; *Case B*: studies only report total fish abundance from multiple sampling seasons within a given year; *Case C*: fish abundances sampled/reported more than once per year, averaged per year and then averaged across all *Before* year and all *After* years (averages of averages). Cases are compared to all meta-analysable data, which includes all cases of *A*, *B* and *C*.

***Biomass***


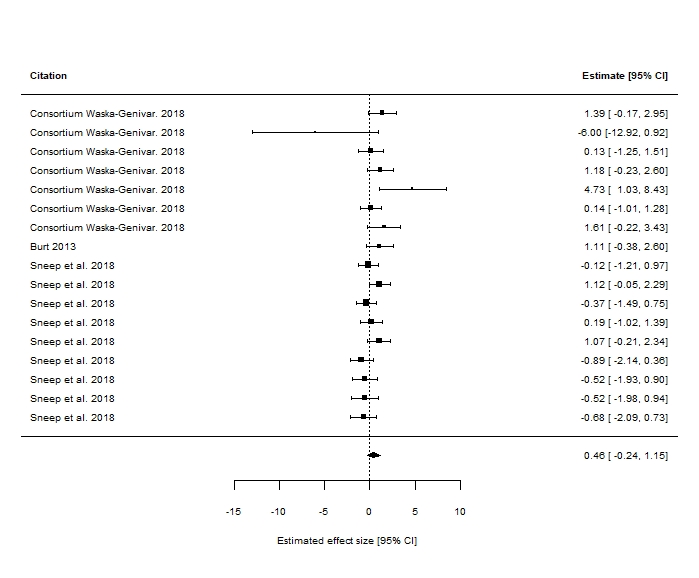


Fig. S24. Summary plot of all effect size estimates from interannual *Before/After* evaluations of the impact of flow magnitude alterations on fish biomass (*k*=17). Error bars indicate 95% confidence intervals. A positive mean value (right of dashed zero line) indicates that the biomass was higher in the *After* period than in the *Before* period (no intervention). Diamond: overall mean effect size of random-effects model.


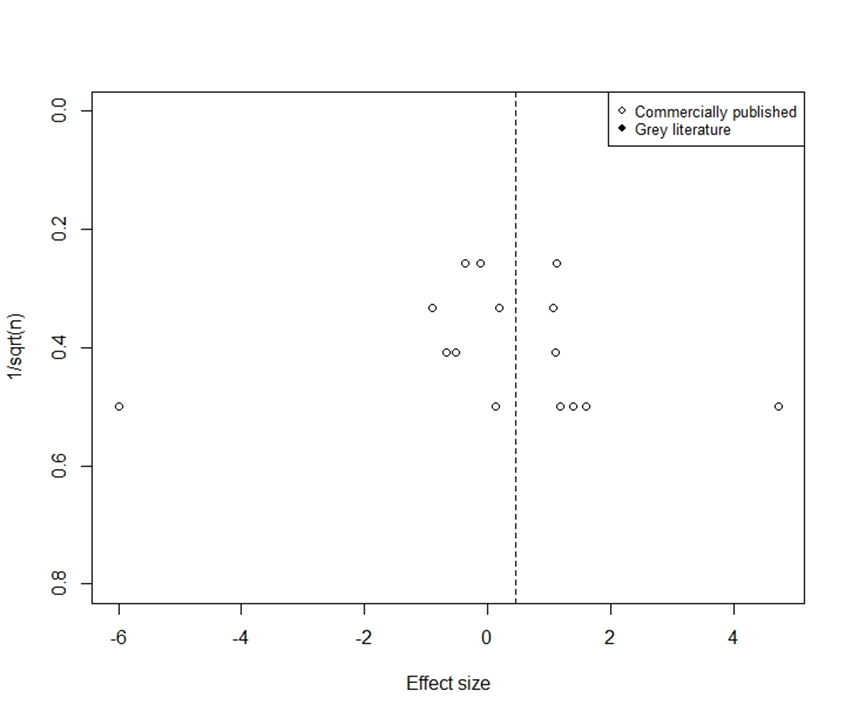


Fig. S25. Funnel plot showing *Control/Impact* studies of abundance (*k* = 77). Open circles indicate datasets from commercially published articles and filled circles indicate datasets from grey literature. Summary effect is indicated by the dashed line.

A single effect size was significant and positive, but no points were highly influential (Fig. S26).


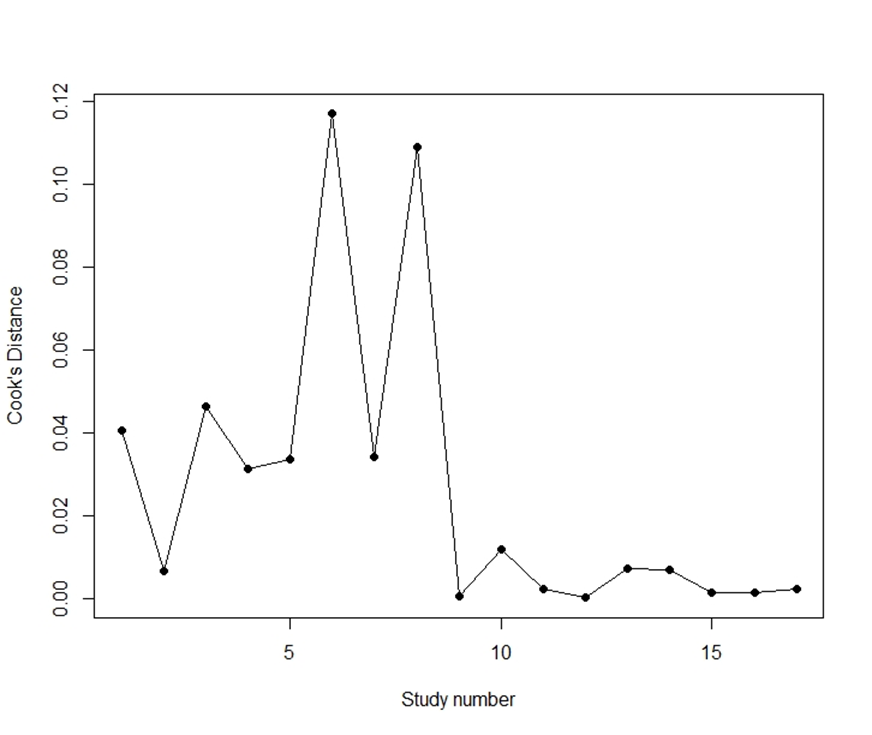


Fig. S26. Cook’s distance plot indicating influence of effect size for biomass interannual *Before/After* studies.

As with abundance, there was one study with multiple interventions that could have been compared to either the same comparator, or different comparator periods. Changing the comparator had little impact on the mean effect size (Fig. S27).


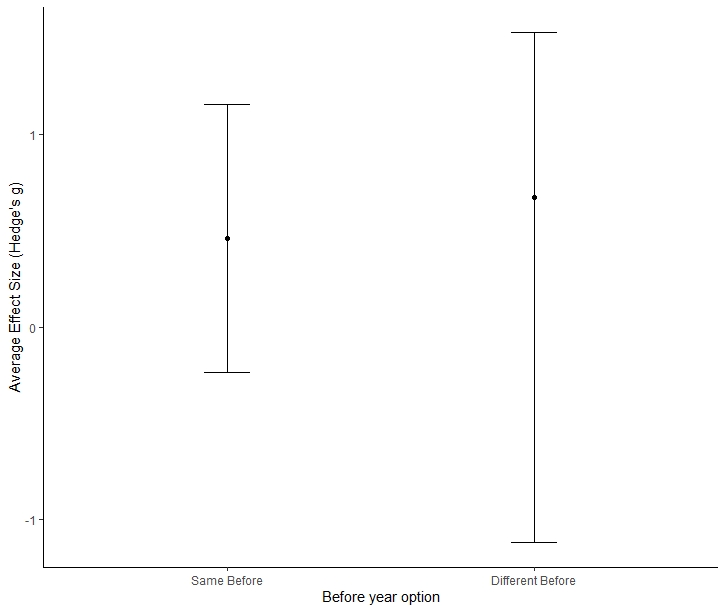


Fig. S27. Comparison of interannual *Before/After* studies overall biomass effect size when datasets from studies with more than one intervention are compared to a single pre-trial *Before* period (Same *Before*) or when each subsequent intervention is compared to the previous period (i.e., Trial 2 is compared to Trial 1, rather than to the pre-trial period).

Sensitivity analysis was not conducted to assess the impact of including studies with imputation, studies that did not report flow magnitude components, deficient *BA* or *BACI* designs, or the inclusion of outflow regions because no datasets had these features. Sensitivity analysis of medium validity studies showed a slightly smaller, but still positive, effect of flow magnitude alterations on fish biomass compared to the overall meta-analysis, as did the sensitivity analysis of studies where no influence of stocking was present (Table S5). The effect sizes for these sensitivity analyses were not significant, indicating that the results of the random effects model may be robust to the inclusion of low validity studies or studies that may have been influenced by stocking. Sensitivity analysis of studies with only a single intervention (i.e., one alteration to flow during the study, rather than several alterations being compared to a single *Before* period) showed a larger, more positive effect size compared to the overall meta-analysis. Sensitivity analysis of studies that reported single data points or summed abundance per year, showed a larger, more positive effect size compared to the overall meta-analysis which included studies where yearly averages, averaged for the *Before* or *After* period (i.e., averages of averages) were used (Table S5). In both cases, the effect size for these sensitivity analyses were significant, indicating that the results of the random effects model may not be robust to the inclusion of studies with more than one intervention, or that report averages of averages.

Table S5. Summary statistics of applicable sensitivity analyses for interannual *BA* study designs and biomass. Statistical significance at *p*<0.05.

| **Analysis** | **Standardized mean difference (Hedge’s *g*)** |
| --- | --- |
| Global analysis (*k* = 17) | 0.46 (95% CI -0.24, 1.15; *p* = 0.196) |
| Medium Validity (*k* = 16) | 0.34 (95% CI -0.46, 1.14; *p* = 0.403) |
| Without imputation (*k* = 17) | N/A |
| Without stocking (*k* = 16) | 0.34 (95% CI -0.46, 1.14; *p* = 0.403) |
| Single comparator/single intervention (*k* = 8) | 0.83 (95% CI 0.26, 1.41; *p* = 0.004) |
| Reported flow magnitude components (*k* = 17) | N/A |
| Without deficient BA or BACI (*k* = 17) | N/A |
| Without averages of averages (*k* = 7) | 0.79 (95% CI 0.17, 1.41; *p* = 0.013) |
| Inclusion of outflow regions (*k* = 17) | N/A |

Note, a decrease in the abundance of fish from alterations to flow magnitude due to HPP compared to control groups is indicated by a value <0 for Hedges’ *g*. CI: 95% confidence interval. *k*: number of effect sizes. N/A: unable to assess moderator due to insufficient sample size or lack of variation

### Effects of moderators – Interannual Before/After studies

**Abundance**

##### *Monitoring duration*

Due to two extreme outliers in effect sizes (*Ictalurus punctatus* and *Micropterus dolomieu*; (Bestgen et al. 2006), we were unable to achieve normality through transformation for the continuous moderator ‘monitoring duration’. We therefore conducted meta-regression with and without these outliers and present results for both analyses below (Fig. S28 and S29). We found no significant relationship of fish abundance and monitoring duration in either instance and the results of the two models did not differ greatly. We report results of *Q_M_* for the models in Table S6. Potential reasons for the occurrence of these outliers for *Ictalurus punctatus* (ES = 14) and *Micropterus dolomieu* (ES = 21), may be due to factors other than flow magnitude alterations that occurred in the *Before* and *After* periods of the study (Bestgen et al. 2006). During the *Before* sampling period, *I. punctatus* had the lowest numbers ever recorded in the history of sampling in the system; this results in a comparison between the *After* period and a non-representative *Before* period which might inflate the effect size. *Micropterus dolomieu* is an established invader with an active removal program, conducted throughout the *Before* and *After* periods. This species had a very successful spawning year during the *After* sampling period, probably due to warm water temperatures, which led to an extreme number of age-1 fish (Bestgen et al. 2006). The *Before* period may have had a depressed number of fish due to active removal and the *After* period had an unexpectedly high number of age-1 fish, resulting in an inflated effect size.


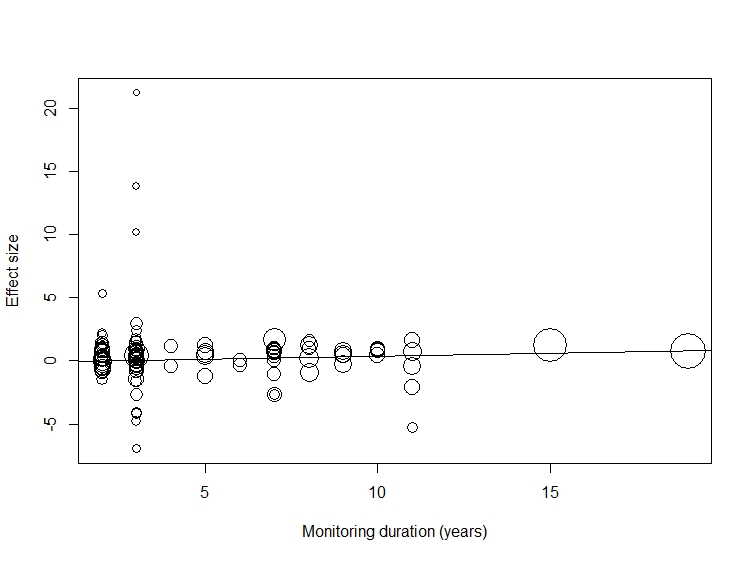


Fig. S28. Meta-regression of effect sizes (Hedge’s *g*) against monitoring duration (years) for interannual *Before/After* studies and abundance. Two extreme effect sizes were retained in this model to compare to model without outliers.


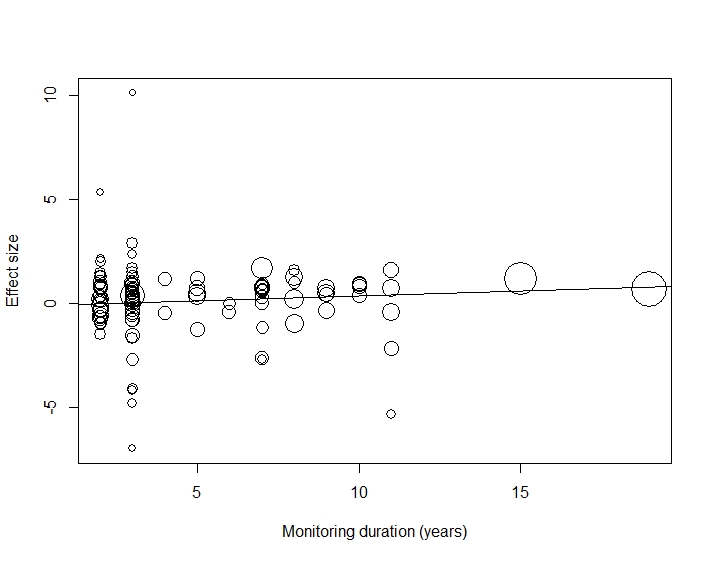


Fig. S29. Meta-regression of effect sizes (Hedge’s *g*) against monitoring duration (years) for interannual *Before/After* studies and abundance. Two extreme effect sizes were removed to improve model fit.

Table S6. Summary results of meta-regression using subsets of fish abundance effect sizes for interannual *Before/After* studies, testing the influence of monitoring duration with and without extreme outliers.

| **Moderator** | ***k*** | ***Q* statistic (p-value)** | ***Q_M_* (*p*-value)** | ***Q_E_* (*p*-value)** |
| --- | --- | --- | --- | --- |
| Monitoring duration (with outliers)  Unmoderated model  Monitoring duration | 112  112 | **421.12 (*p*<0.0001)**  - | -  1.32 (*p*=0.252) | -  **417.43 (*p*<0.0001)** |
| Monitoring duration (without outliers)  Unmoderated model  Monitoring duration | 110  110 | **361.71 (*p*<0.0001)**  - | -  1.39 (*p*=0.239) | -  **357.43 (*p*<0.0001)** |

Unmoderated model: random-effects model; *k*: number of effect sizes; *Q* statistic: value of homogeneity test; *Q_m_*: omnibus test statistic of moderators; *Q_E_*: unexplained heterogeneity. Significance at *p* < 0.05; * Significance at *p*

<0.1.


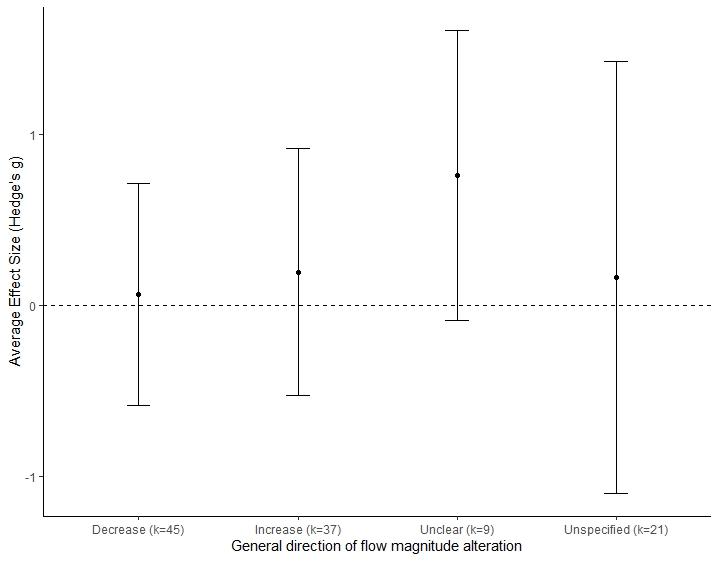


Fig. S30. Average effect size by intervention for fish abundance responses when considering interannual *BA* studies. Value in parentheses (*k*) is the number of effect sizes. Error bars indicate 95% confidence intervals. A positive mean value (above the dashed zero line) indicates that the abundance was higher in the *After* period (intervention) than in the *Before* period (no intervention). 95% confidence intervals that do not overlap with the dashed line indicate a significant effect (at the *p<*0.05 level). *Decrease*: flow magnitude in *After* period decreases in relation to *Before* period; *Increase*: flow magnitude in *After* period increases in relation to the *Before* period; *Unclear*: unclear description of flow magnitude alteration; *Unspecified*: no specified alteration to flow magnitude but change is assumed due to presence of hydropower facility (i.e., before and after the closure of a dam).


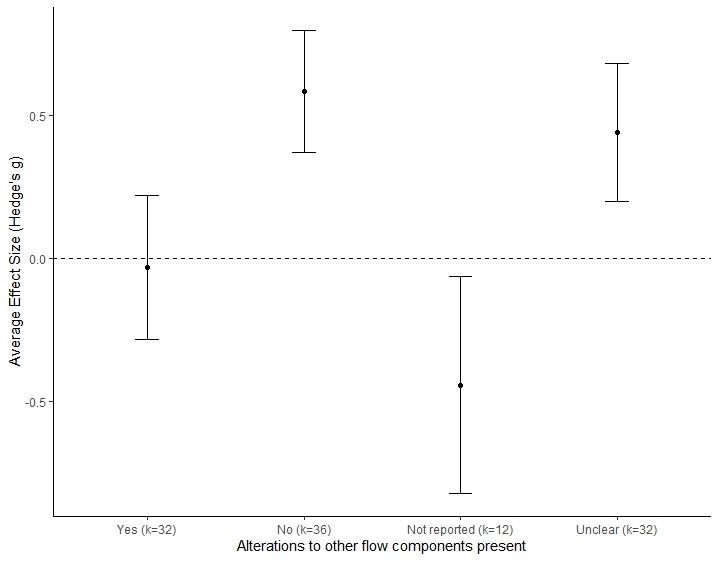


Fig. S31. Average effect size by the presence alterations to other flow components for fish abundance responses when considering interannual *BA* studies. Value in parentheses (*k*) is the number of effect sizes. Error bars indicate 95% confidence intervals. A positive mean value (above the dashed zero line) indicates that the abundance was higher in the *After* period (intervention) than in the *Before* period (no intervention). 95% confidence intervals that do not overlap with the dashed line indicate a significant effect (at the *p<*0.05 level). *Yes*: alterations to other flow components were present; *No*: no alterations to other flow components were present; *Not reported*: no mention of alteration to other flow components; *Unclear*: unclear whether another flow component was altered but other flow components are mentioned by authors.


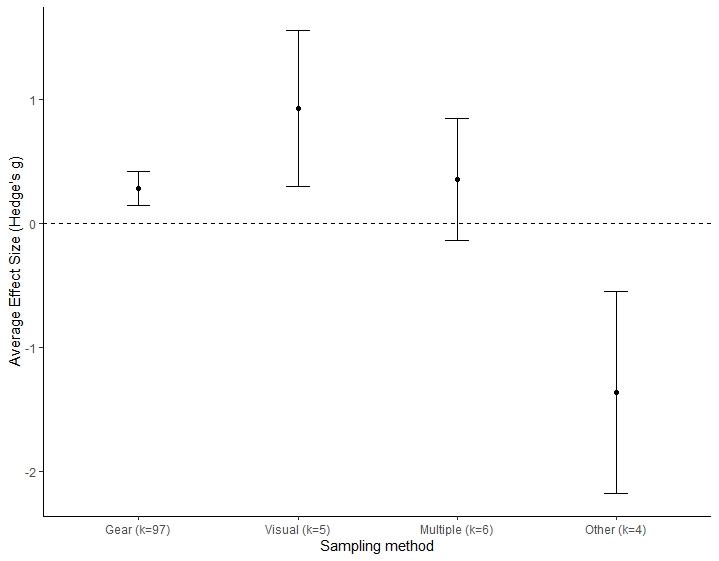


Fig. S32. Average effect size by sampling method for fish abundance responses when considering interannual *BA* studies. Value in parentheses (*k*) is the number of effect sizes. Error bars indicate 95% confidence intervals. A positive mean value (above the dashed zero line) indicates that the abundance was higher in the *After* period (intervention) than in the *Before* period (no intervention). 95% confidence intervals that do not overlap with the dashed line indicate a significant effect (at the *p<*0.05 level). *Multiple*: two or more different sampling methods used; *Othe*r: any other methods not previously mentioned (i.e., historical catch data or hydroacoustics).


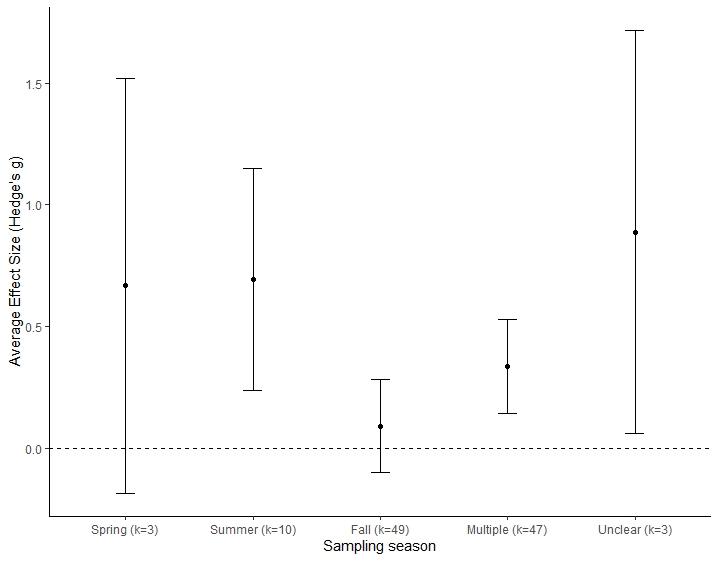


Fig. S33. Average effect size by sampling season for fish abundance responses when considering interannual *BA* studies. Value in parentheses (*k*) is the number of effect sizes. Error bars indicate 95% confidence intervals. A positive mean value (above the dashed zero line) indicates that the abundance was higher in the *After* period (intervention) than in the *Before* period (no intervention). 95% confidence intervals that do not overlap with the dashed line indicate a significant effect (at the *p<*0.05 level). *Multiple*: two or more different sampling seasons used; *Unclear*: no clear description of sampling season included.


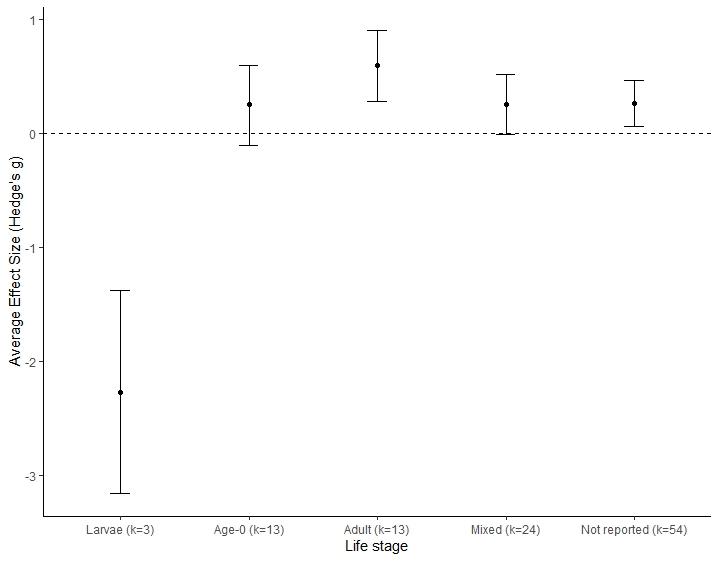


Fig. S34. Average effect size by fish life stage for fish abundance responses when considering interannual *BA* studies. Value in parentheses (*k*) is the number of effect sizes. Error bars indicate 95% confidence intervals. A positive mean value (above the dashed zero line) indicates that the abundance was higher in the *After* period (intervention) than in the *Before* period (no intervention). 95% confidence intervals that do not overlap with the dashed line indicate a significant effect (at the *p*<0.05 level). *Mixed*: two or more life stages not reported separately; *Not reported*: no life stage specified.

**References**

Bestgen, K. R., K. A. Zelasko, R. I. Compton, and T. Chart. 2006. Responses of the Green River fish community to changes in flow and temperature regimes from Flaming Gorge Dam since 1996 based on sampling conducted from 2002 to 2004. Larval Fish Laboratory, Colorado State University, Final Report to the Colorado River Recovery Implementation Program, Project 115, Denver.
