# Supplementary material for: How do changes in flow magnitude due to hydropower operations affect fish abundance and biomass in temperate regions? A systematic review
Source: Environ Evid. 2022 Feb 4;11:3. doi: 10.1186/s13750-021-00254-8 (PMC8813579; doi:10.1186/s13750-021-00254-8)
Supplement: Supplementary file 13 — Additional file 13. Taxonomic analysis. Includes forest plots for all families with sufficient sample sizes and for, families with significant heterogeneity, genera therein with sufficient sample size for further analysis (i.e., ≥ 3 datasets from ≥ 2 independent studies. [file 13750_2021_254_MOESM13_ESM.docx]

**Additional File 13. Taxonomic analysis**

Description: Includes forest plots for all families with sufficient sample sizes and for, families with significant heterogeneity, genera therein with sufficient sample size for further analysis (i.e., ≥3 datasets from ≥2 independent studies.

***Control/Impact* Studies: Family**

***Abundance***

*
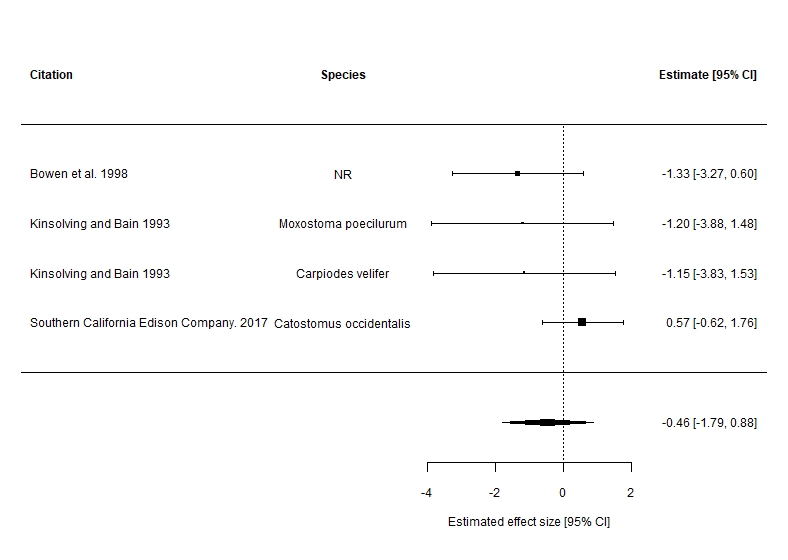
* Fig. S1. Summary plot of all effect size estimates from *Control/Impact* evaluations of the impact of flow magnitude alterations on the abundance of the family Catostomidae (*k*=4). Error bars indicate 95% confidence intervals. A positive mean value (right of dashed zero line) indicates that the abundance was higher in treatment areas than in comparator areas (no intervention). NR: species not reported. Diamond: overall mean effect size of random-effects model.


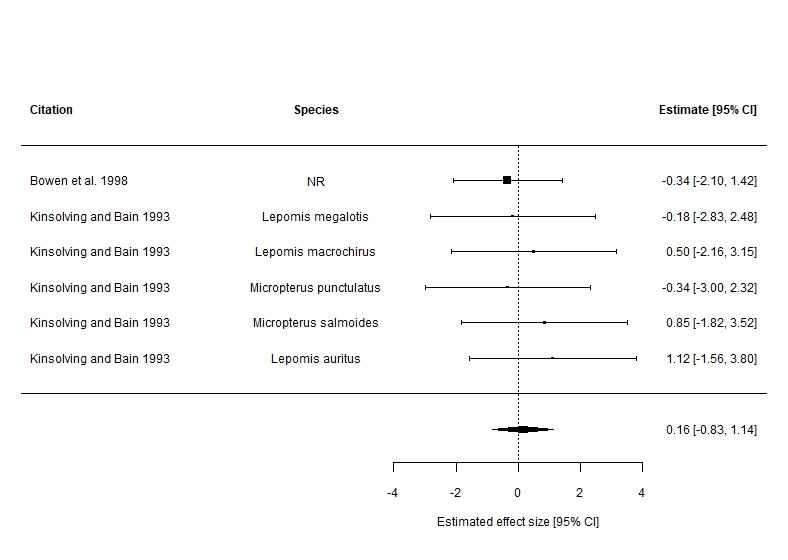


Fig. S2. Summary plot of all effect size estimates from *Control/Impact* evaluations of the impact of flow magnitude alterations on the abundance of the family Centrarchidae (*k*=6). Error bars indicate 95% confidence intervals. A positive mean value (right of dashed zero line) indicates that the abundance was higher in treatment areas than in comparator areas (no intervention). NR: species not reported. Diamond: overall mean effect size of random-effects model.


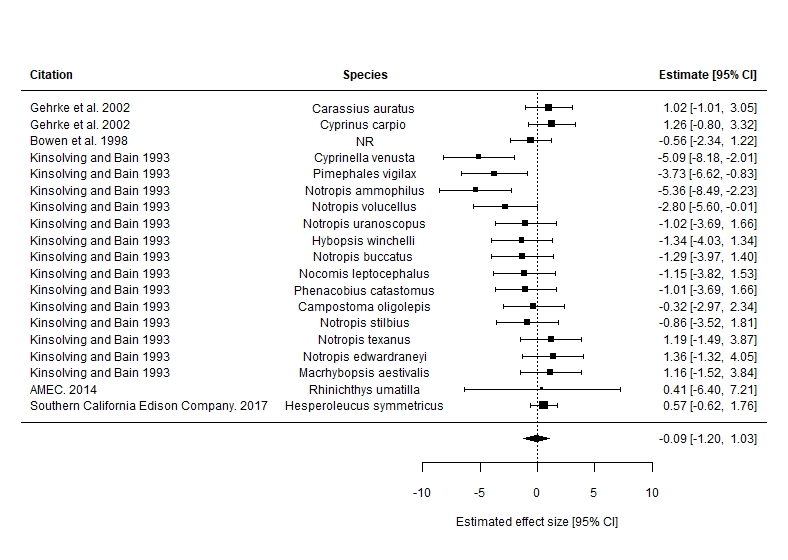


Fig. S3. Summary plot of all effect size estimates from *Control/Impact* evaluations of the impact of flow magnitude alterations on the abundance of the family Cyprinidae (*k*=19). Error bars indicate 95% confidence intervals. A positive mean value (right of dashed zero line) indicates that the abundance was higher in treatment areas than in comparator areas (no intervention). NR: species not reported. Diamond: overall mean effect size of random-effects model.

*
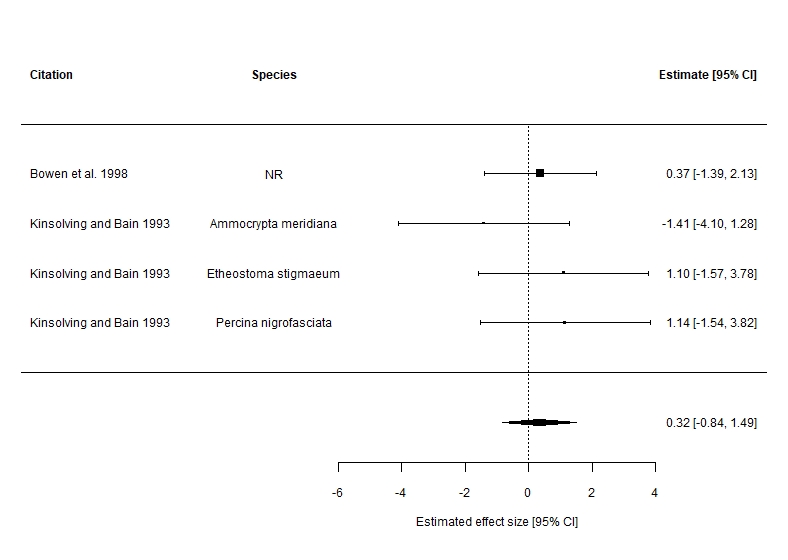
*Fig. S4. Summary plot of all effect size estimates from *Control/Impact* evaluations of the impact of flow magnitude alterations on the abundance of the family Percidae (*k*=4). Error bars indicate 95% confidence intervals. A positive mean value (right of dashed zero line) indicates that the abundance was higher in treatment areas than in comparator areas (no intervention). NR: species not reported. Diamond: overall mean effect size of random-effects model.


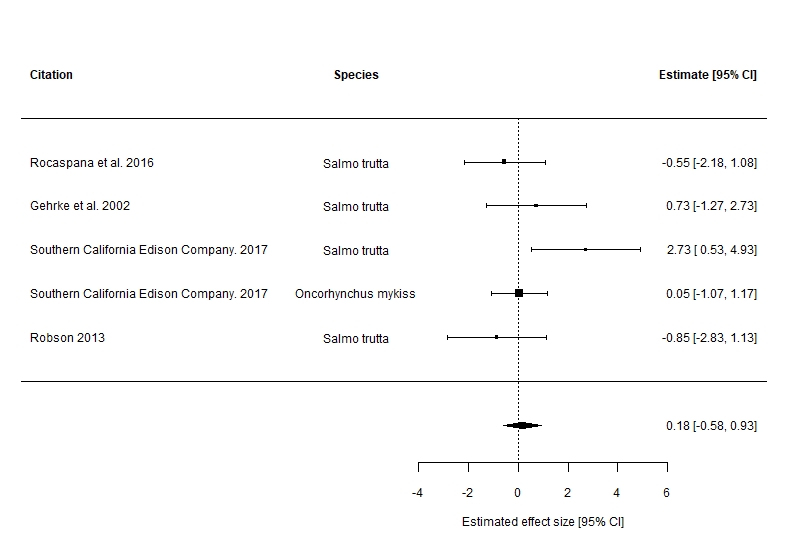


Fig. S5. Summary plot of all effect size estimates from *Control/Impact* evaluations of the impact of flow magnitude alterations on the abundance of the family Salmonidae (*k*=5). Error bars indicate 95% confidence intervals. A positive mean value (right of dashed zero line) indicates that the abundance was higher in treatment areas than in comparator areas (no intervention). Diamond: overall mean effect size of random-effects model.

Based on the *Q* test of heterogeneity, heterogeneity among effect sizes for the Catostomidae family was not significant (*Q*=3.95, *p* = 0.266). Three species (*Moxostoma poecilurum*, *Carpiodes velifer*, and *Catostomus occidentalis*) and one unknown species were present in this group. Similarly, there was no statistically significant heterogeneity among effect sizes for species of Centrarchidae family (*Q* = 1.32, *p* = 0.93), which was represented by five species from two known genera [*Lepomis* (*k*= 2) and *Micropterus* (*k*=3)] and one unknown genera, Percidae family [represented by three known and one unknown genera (Q = 2.2745, p = 0.5174)] or Salmonidae family (*Q* = 7.33, *p* = 0.119) which was primarily *Salmo trutta* (5 of 6 datasets). In contrast, the *Q* test for heterogeneity indicated that there was significant heterogeneity among species effect sizes for Cyprinidae (*Q* = 39.41, *p* = 0.003) which could be explored through moderator analysis, however 14 of 19 effect sizes were from a single study and there was insufficient variation in moderators to allow effective evaluation of the influence of moderator variables within the outcome subgroup. Sample sizes were too small for moderator analysis of other families and abundance, or for analyzing biomass responses by taxa.

**Within-year *Before/After* Studies: Family**

***Abundance***

*
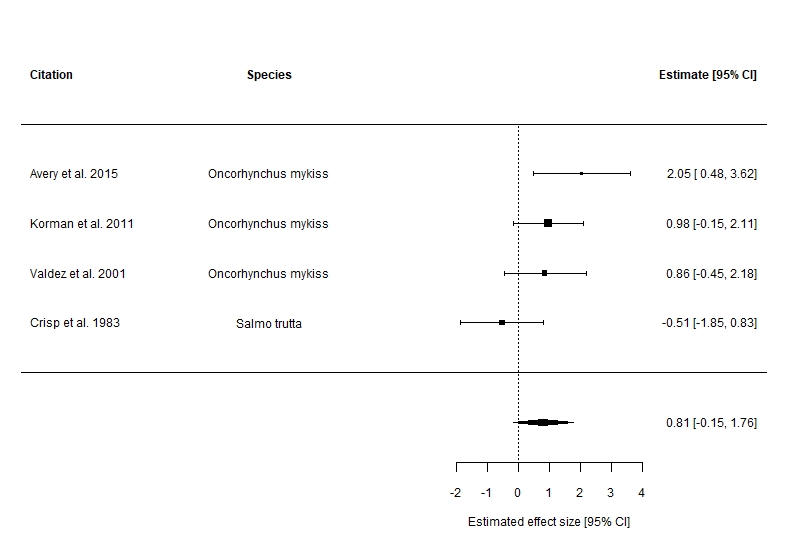
* Fig. S6. Summary plot of all effect size estimates from with-in year *Before/After* studies considering post-intervention year-1 evaluations of the impact of flow magnitude alterations on the abundance of the family Salmonidae (*k*=4). Error bars indicate 95% confidence intervals. A positive mean value (right of dashed zero line) indicates that the abundance was higher in *Afte*r period than in *Before* period (no intervention). Diamond: overall mean effect size of random-effects model.

**Interannual *Before/After* Studies: Family**

***Abundance***

*
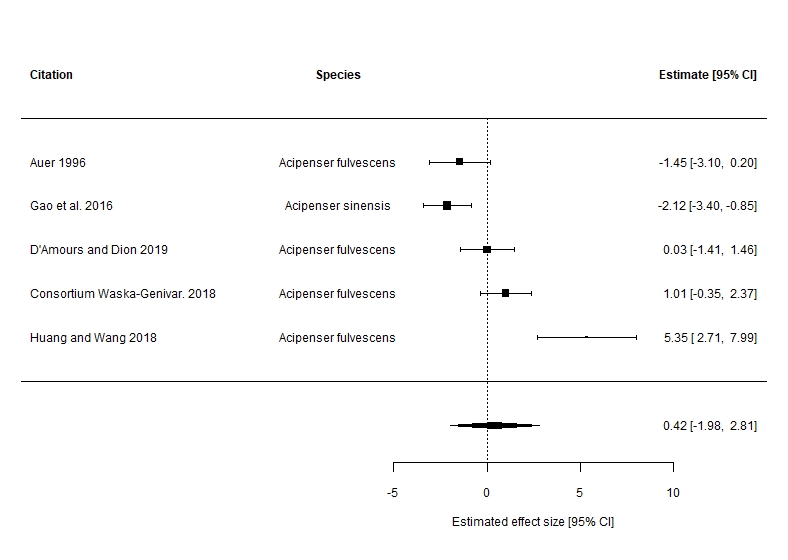
* Fig. S7. Summary plot of all effect size estimates from interannual *Before/After* studies considering evaluations of the impact of flow magnitude alterations on the abundance of the family Acipenseridae (*k*=5). Error bars indicate 95% confidence intervals. A positive mean value (right of dashed zero line) indicates that the abundance was higher in *Afte*r period than in *Before* period (no intervention). Diamond: overall mean effect size of random-effects model.

*
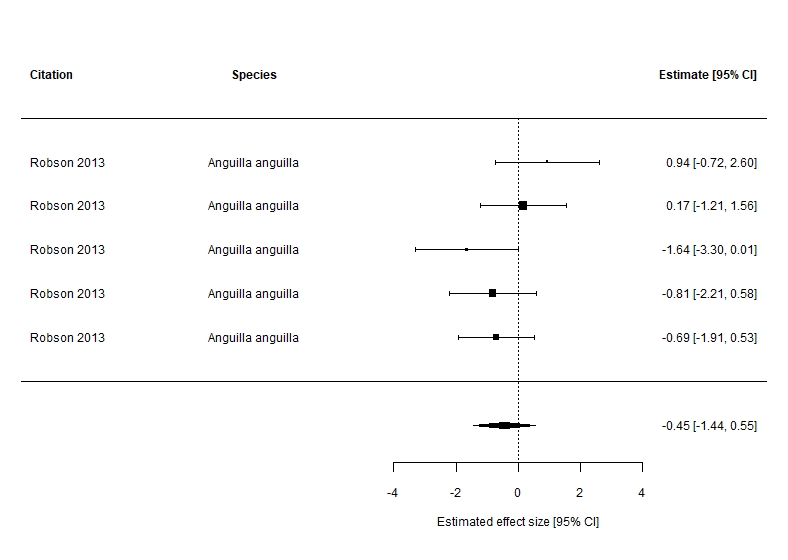
* Fig. S8. Summary plot of all effect size estimates from interannual *Before/After* studies considering evaluations of the impact of flow magnitude alterations on the abundance of the family Anguillidae (*k*=5). Error bars indicate 95% confidence intervals. A positive mean value (right of dashed zero line) indicates that the abundance was higher in *Afte*r period than in *Before* period (no intervention). Diamond: overall mean effect size of random-effects model.


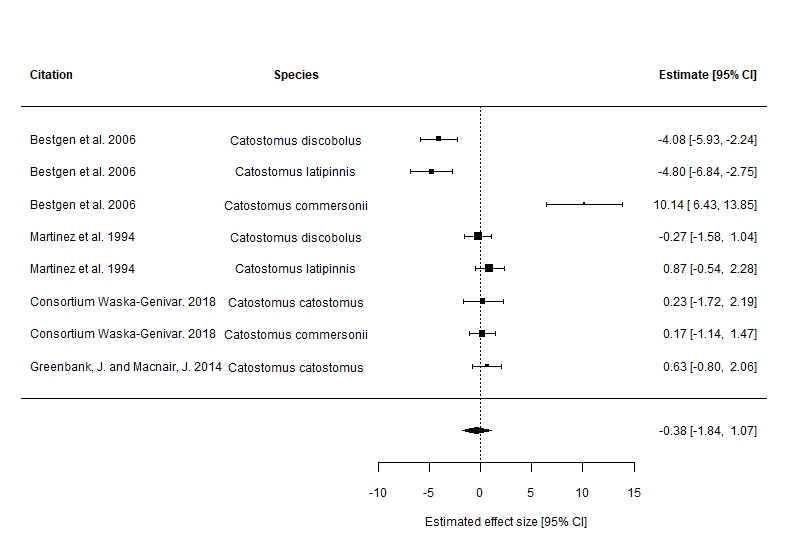


Fig. S9. Summary plot of all effect size estimates from interannual *Before/After* studies considering the impact of flow magnitude alterations on abundance of the family Catostomidae (*k*=8). Error bars indicate 95% confidence intervals. A positive mean value (right of dashed zero line) indicates that the abundance was higher in *Afte*r period than in *Before* period (no intervention). Diamond: overall mean effect size of random-effects model.

*
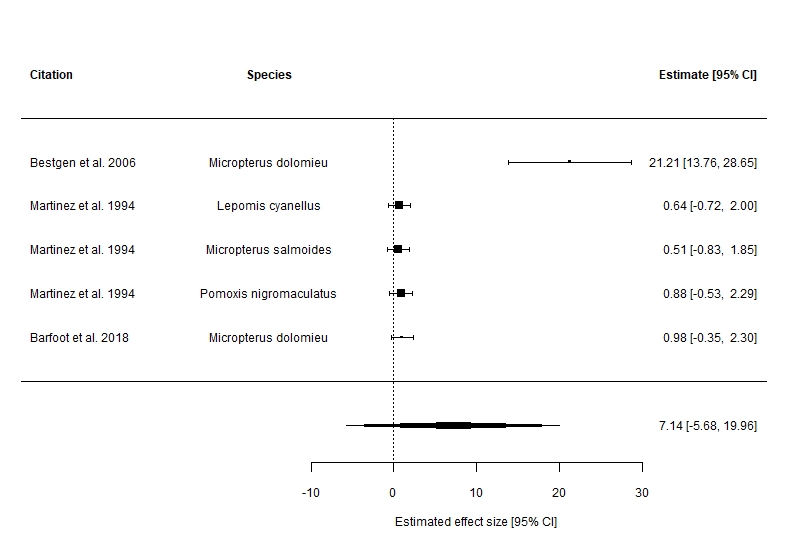
* Fig. S10. Summary plot of all effect size estimates from interannual *Before/After* studies considering the impact of flow magnitude alterations on abundance of the family Centrarchidae (*k*=5). Error bars indicate 95% confidence intervals. A positive mean value (right of dashed zero line) indicates that the abundance was higher in *Afte*r period than in *Before* period (no intervention). Diamond: overall mean effect size of random-effects model.

*
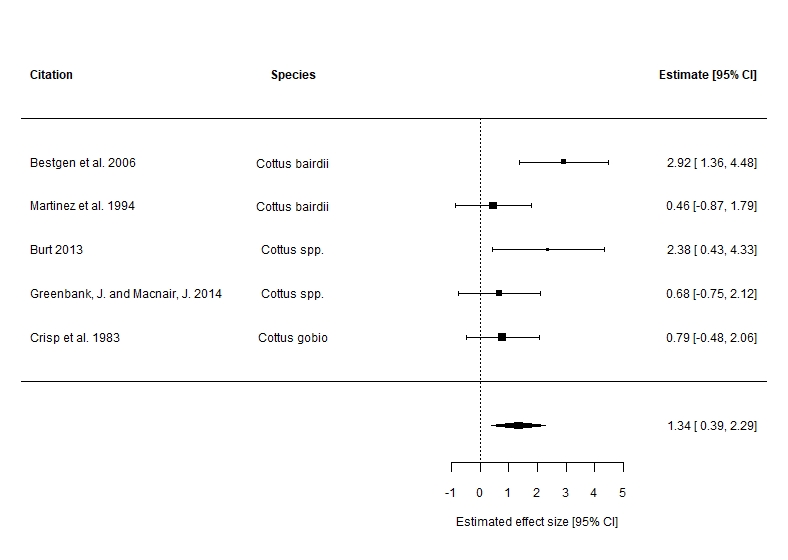
* Fig. S11. Summary plot of all effect size estimates from interannual *Before/After* studies considering the impact of flow magnitude alterations on abundance of the family Cottidae (*k*=5). Error bars indicate 95% confidence intervals. A positive mean value (right of dashed zero line) indicates that the abundance was higher in *Afte*r period than in *Before* period (no intervention). Diamond: overall mean effect size of random-effects model.


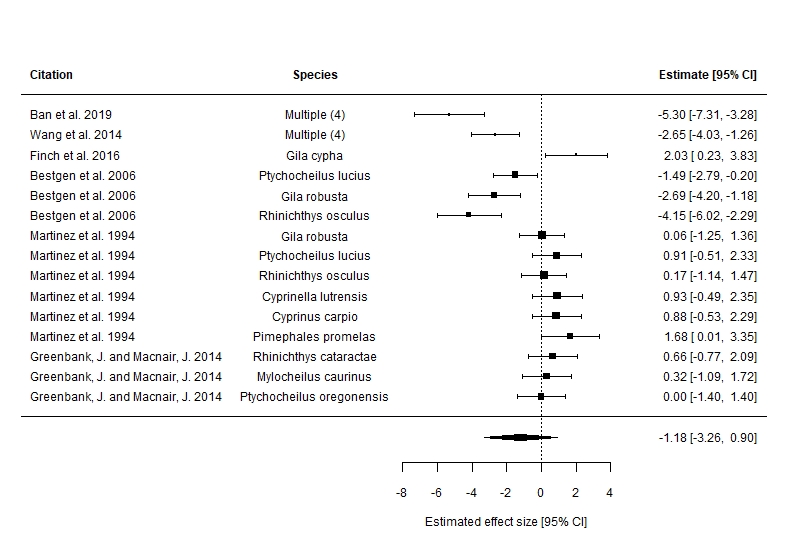


Fig. S12. Summary plot of all effect size estimates from interannual *Before/After* studies considering the impact of flow magnitude alterations on abundance of the family Cyprinidae (*k*=5). Error bars indicate 95% confidence intervals. A positive mean value (right of dashed zero line) indicates that the abundance was higher in *Afte*r period than in *Before* period (no intervention). Diamond: overall mean effect size of random-effects model. *Multiple*: more than one species included in pooled data (number in brackets indicates number of species included).

*
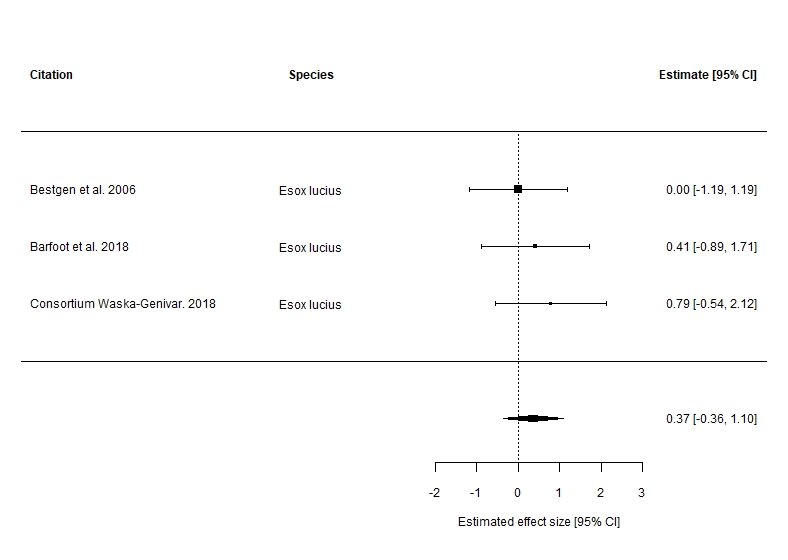
* Fig. S13. Summary plot of all effect size estimates from interannual *Before/After* studies considering the impact of flow magnitude alterations on abundance of the family Esocidae (*k*=3). Error bars indicate 95% confidence intervals. A positive mean value (right of dashed zero line) indicates that the abundance was higher in *Afte*r period than in *Before* period (no intervention). Diamond: overall mean effect size of random-effects model.

*
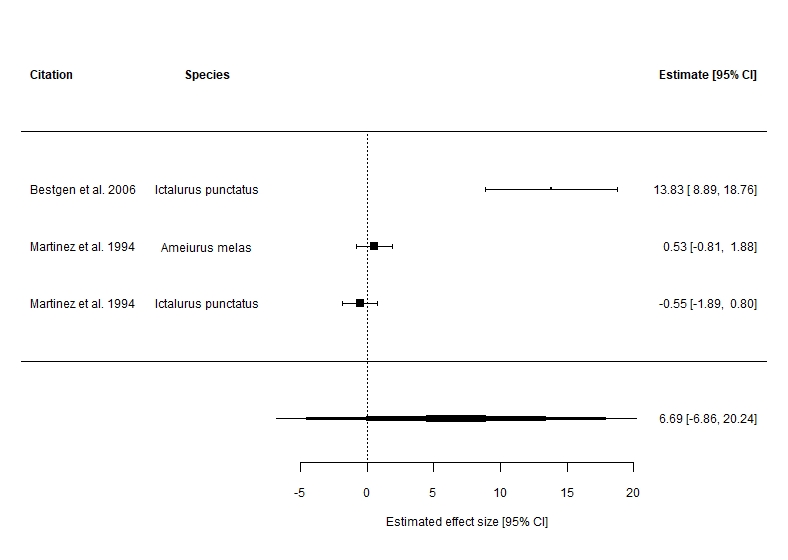
* Fig. S14. Summary plot of all effect size estimates from interannual *Before/After* studies considering the impact of flow magnitude alterations on abundance of the family Ictaluridae (*k*=3). Error bars indicate 95% confidence intervals. A positive mean value (right of dashed zero line) indicates that the abundance was higher in *Afte*r period than in *Before* period (no intervention). Diamond: overall mean effect size of random-effects model.

*
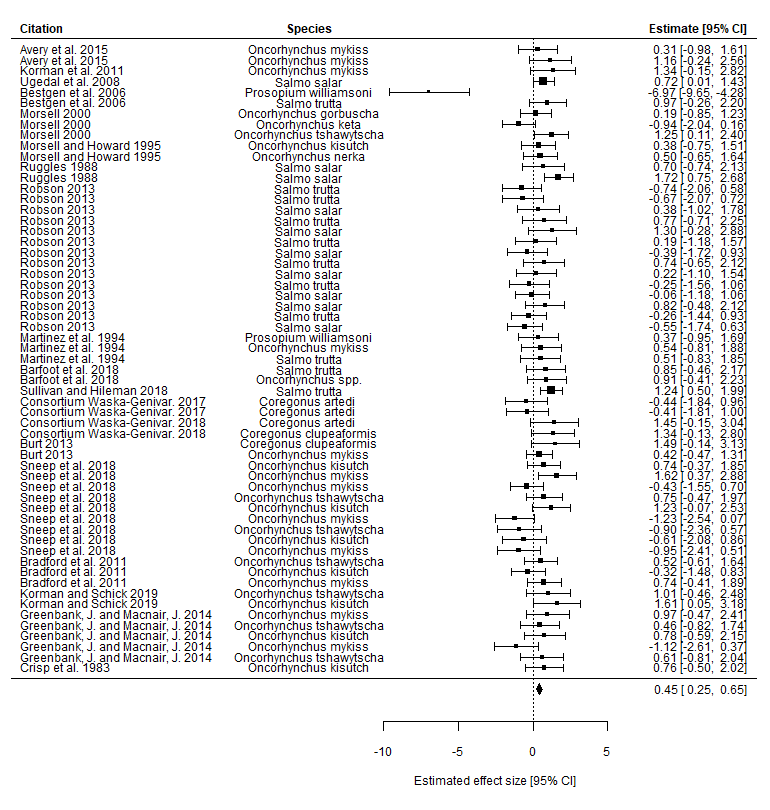
* Fig. S15. Summary plot of all effect size estimates from interannual *Before/After* studies considering the impact of flow magnitude alterations on abundance of the family Salmonidae (*k*=59). Error bars indicate 95% confidence intervals. A positive mean value (right of dashed zero line) indicates that the abundance was higher in *Afte*r period than in *Before* period (no intervention). Diamond: overall mean effect size of random-effects model.

**Biomass**

*
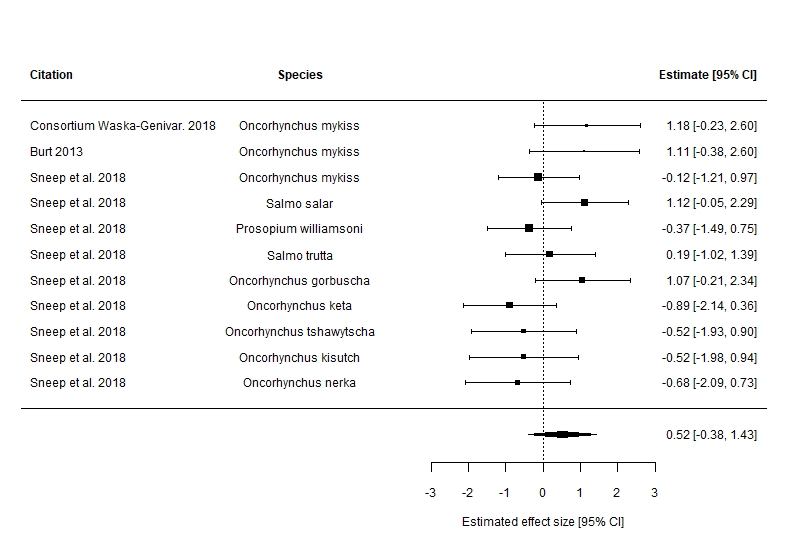
* Fig. S16. Summary plot of all effect size estimates from interannual *Before/After* studies considering the impact of flow magnitude alterations on biomass of the family Salmonidae (*k*=59). Error bars indicate 95% confidence intervals. A positive mean value (right of dashed zero line) indicates that the abundance was higher in *Afte*r period than in *Before* period (no intervention). Diamond: overall mean effect size of random-effects model.

**Interannual *Before/After Studies*: Genera**

***Abundance***

Based on the *Q* test of heterogeneity, there was also significant heterogeneity among effect sizes for Acipenseridae (*Q* = 31.09, *p* <0.0001) which included only two species (*Acipenser fulvescens*, *Acipenser sinensis*), Catostomidae (*Q* = 70.26, *p* <0.0001) which included five species from one genus, Cyprinidae [15 species from 11 genera due to combined species outcomes; (*Q* = 87.3019; *p* <0.0001)], and Salmonidae [13 species from five genera (*Q* = 108.13; *p*<0.0001)]. Although heterogeneity was present within these families, we only conducted analyses at the genera level when more than one genus with sufficient sample sizes were present (i.e., Cyprinidae and Salmonidae). Anguillidae (one species; *Q* = 5.88, *p* =0.21), Cottidae (one genus; *Q* = 8.15, *p* = 0.086) and Esocidae (one species; *Q* = 0.76; *p* = 0.685) did not have statistically significant heterogeneity.

For families with statistically significant heterogeneity, we analyzed the response of genera therein, with sufficient sample size (i.e., ≥3 datasets from ≥2 independent studies) and variability to investigate responses in fish abundance to alterations in flow magnitude. Methods follow those used for other taxonomic analyses (see section in main text “Data synthesis and presentation – Quantitative synthesis”).

There were only sufficient sample sizes to investigate genera within Cyprinidae and Salmonidae. Within Cyprinidae, there was sufficient sample size to investigate variation among three genera: (i) *Gila*; (ii) *Ptychocheilus*; and (iii) *Rhinichthys* (Fig S17-S19).

*Gila:*

- Average Hedge's *g* = -0.2311 (95% CI -2.8760, 2.4138; *k* = 3, *p* = 0.8640)
- (*Q* = 16.3347, *p*=0.0003)

*
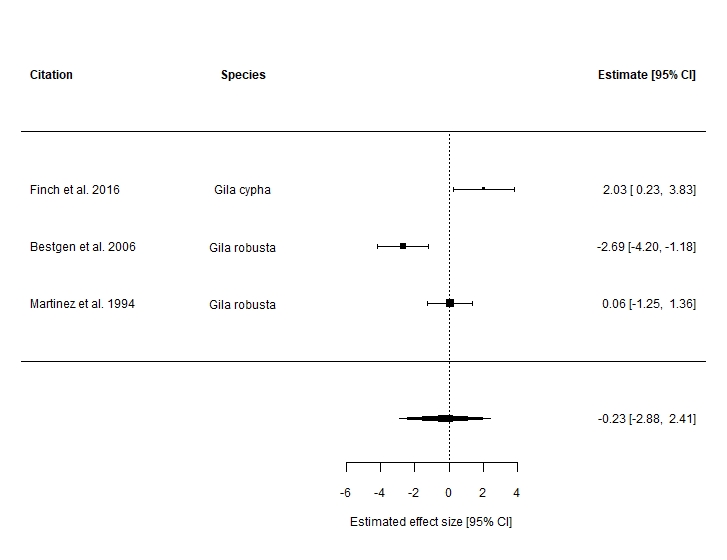
* Fig. S17. Summary plot of all effect size estimates from interannual *Before/After* studies considering the impact of flow magnitude alterations on abundance of the genus *Gila* (*k*= 3). Error bars indicate 95% confidence intervals. A positive mean value (right of dashed zero line) indicates that the abundance was higher in *Afte*r period than in *Before* period (no intervention). Diamond: overall mean effect size of random-effects model.

*Ptychocheilus:*

- Average Hedge's *g* = -0.2197 (95% CI -1.6077, 1.1682; *k* = 3, *p* = 0.7564)
- (*Q* = 6.2363, *p*=0.0442)


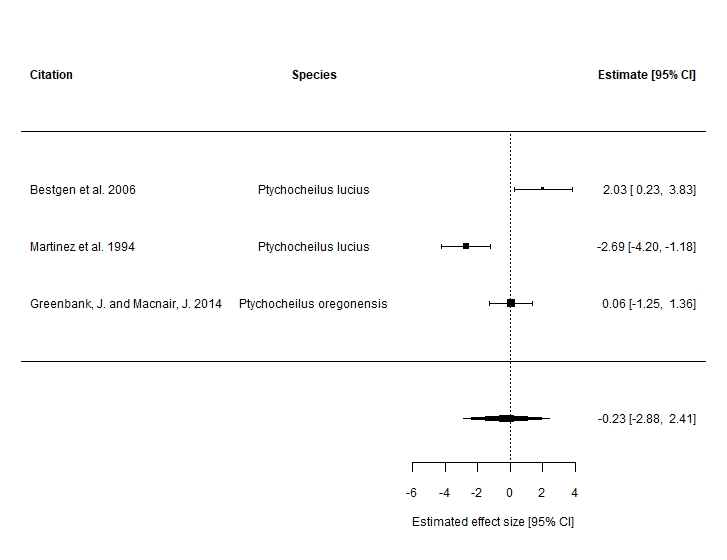


Fig. S18. Summary plot of all effect size estimates from interannual *Before/After* studies considering the impact of flow magnitude alterations on abundance of the genus *Ptychocheilus* (*k*= 3). Error bars indicate 95% confidence intervals. A positive mean value (right of dashed zero line) indicates that the abundance was higher in *Afte*r period than in *Before* period (no intervention). Diamond: overall mean effect size of random-effects model.

*Rhinichthys*

- Average Hedge's *g* = -1.0482 (95% CI -3.9785, 1.8821; *k* = 3, *p* = 0.4833)
- (*Q* = 18.2227, *p*=0.0001)


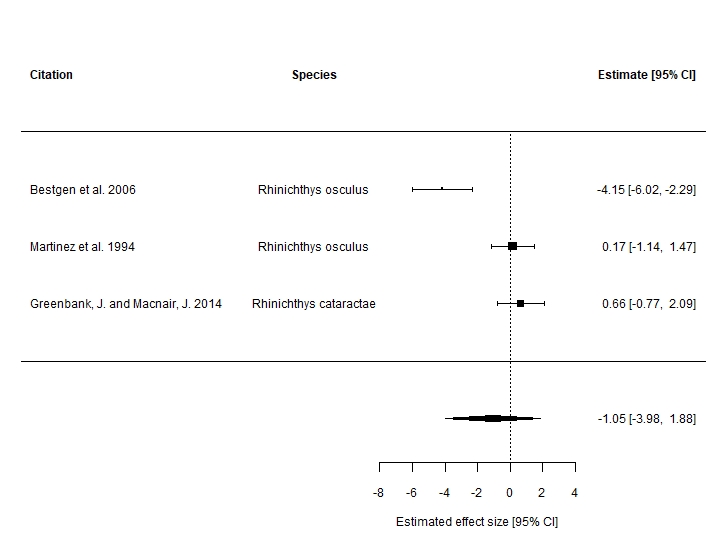


Fig. S19. Summary plot of all effect size estimates from interannual *Before/After* studies considering the impact of flow magnitude alterations on abundance of the genus *Rhinichthys* (*k*= 3). Error bars indicate 95% confidence intervals. A positive mean value (right of dashed zero line) indicates that the abundance was higher in *Afte*r period than in *Before* period (no intervention). Diamond: overall mean effect size of random-effects model.

Within Salmonidae, there was sufficient samples size to investigate variation among four genera: (i) *Coregonus*; (ii) *Oncorhynchus*; and (iii) *Prosopium*; and (iv) *Salmo* (Fig S20-S23).

*Coregonus*

- Average Hedge's *g* = 0.2526 (95% CI -0.9963, 1.5014; *k* = 4, *p* = 0.2526)
- (*Q* = 5.8771, *p*=0.1177)


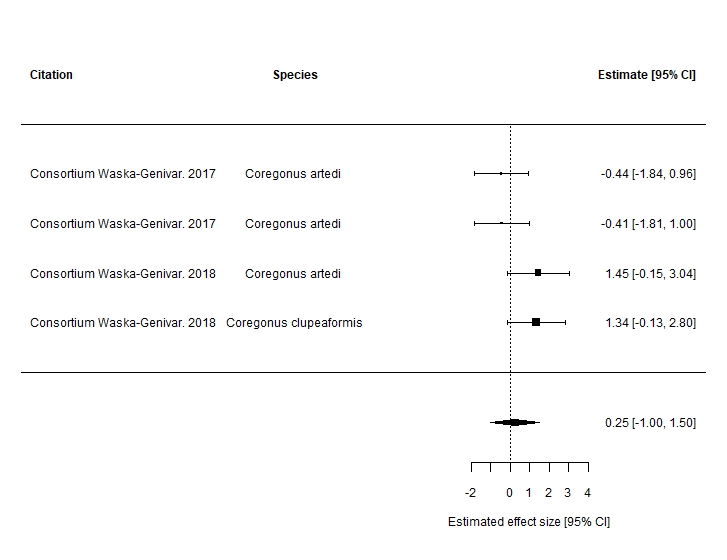


Fig. S20. Summary plot of all effect size estimates from interannual *Before/After* studies considering the impact of flow magnitude alterations on abundance of the genus *Coregonus* (*k*= 4). Error bars indicate 95% confidence intervals. A positive mean value (right of dashed zero line) indicates that the abundance was higher in *Afte*r period than in *Before* period (no intervention). Diamond: overall mean effect size of random-effects model.

*Oncorhynchus*

- Average Hedge's *g* = 0.3649 (95% CI 0.1335, 0.5962; *k* = 29, *p* = 0.0020)
- (*Q* = 42.4169, *p*=0.0396)


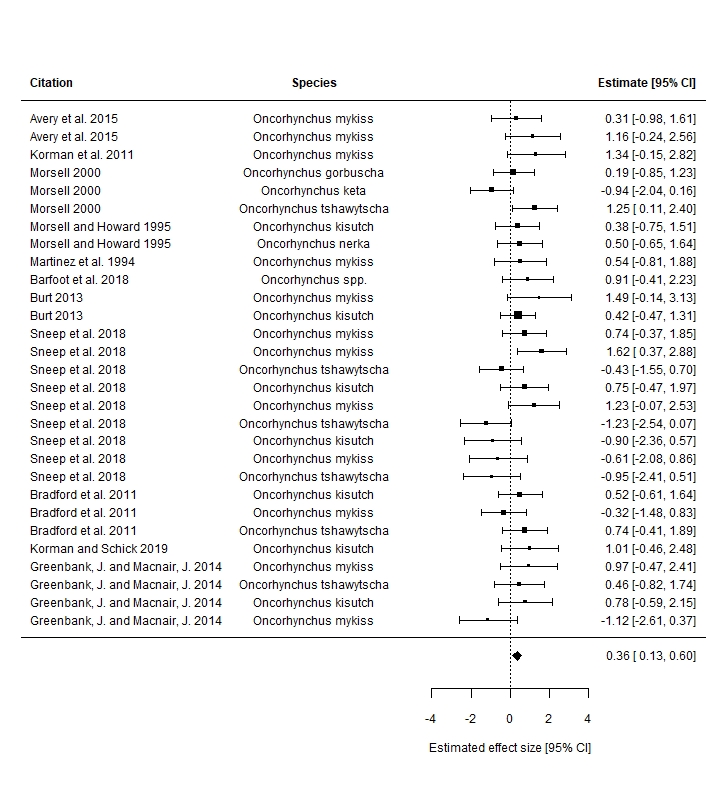


Fig. S21. Summary plot of all effect size estimates from interannual *Before/After* studies considering the impact of flow magnitude alterations on abundance of the genus *Oncorhynchus* (*k*= 29). Error bars indicate 95% confidence intervals. A positive mean value (right of dashed zero line) indicates that the abundance was higher in *Afte*r period than in *Before* period (no intervention). Diamond: overall mean effect size of random-effects model.

*Salmo*

- Average Hedge's *g* = 0.5305 (95% CI 0.1777, 0.8834; *k* = 22, *p* = 0.0032)
- (*Q* = 27.8024, *p*=0.1458)

*
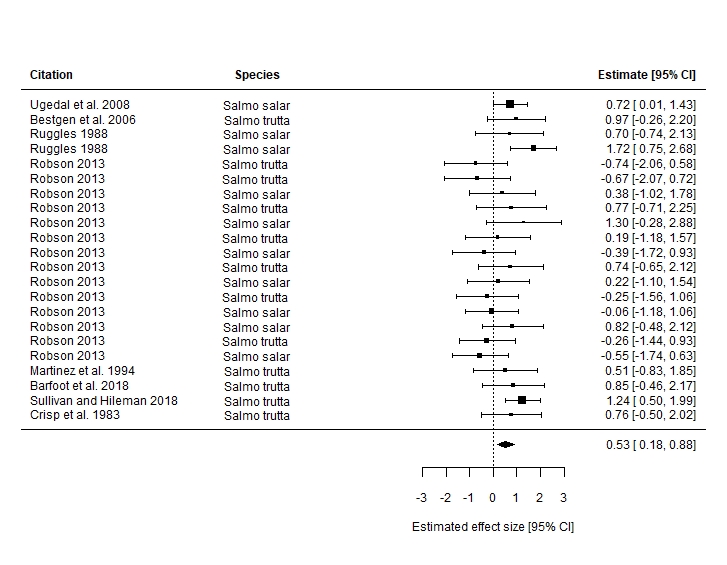
* Fig. S22. Summary plot of all effect size estimates from interannual *Before/After* studies considering the impact of flow magnitude alterations on abundance of the genus *Salmo* (*k*= 22). Error bars indicate 95% confidence intervals. A positive mean value (right of dashed zero line) indicates that the abundance was higher in *Afte*r period than in *Before* period (no intervention). Diamond: overall mean effect size of random-effects model.

**Interannual *Before/After* Studies: Genera**

***Biomass***

For families with statistically significant heterogeneity, we analyzed the response of genera therein, with sufficient sample size (i.e., ≥3 datasets from ≥2 independent studies) and variability to investigate responses in fish abundance to alterations in flow magnitude. Methods follow those used for other taxonomic analyses (see section in main text “Data synthesis and presentation – Quantitative synthesis”).

There were only sufficient sample sizes to investigate genera within Salmonidae for the genus *Oncorhynchus.*

*Oncorhynhcus*

- Average Hedge's *g* = 0.3068 (95% CI -0.7191, 1.3326; *k* = 10, *p* = 0.5578)
- (*Q* = 12.7253, *p*=0.1754)


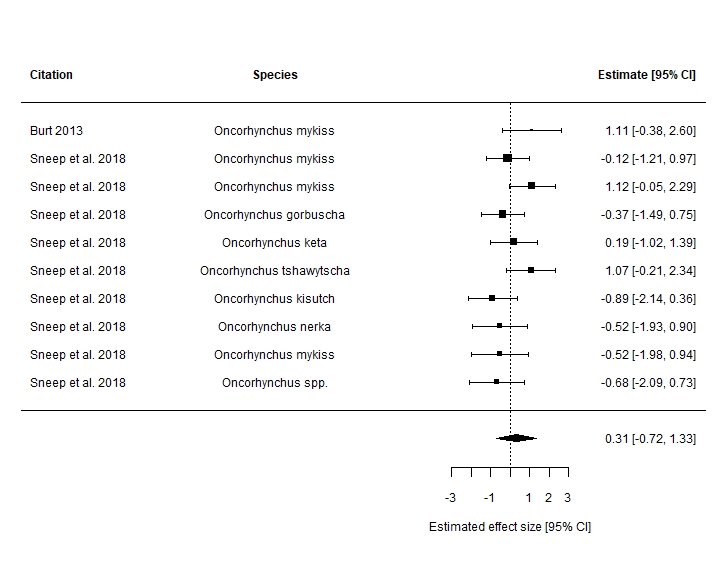


Fig. S23. Summary plot of all effect size estimates from interannual *Before/After* studies considering the impact of flow magnitude alterations on abundance of the genus *Oncorhynchus* (*k*= 10). Error bars indicated 95% confidence intervals. A positive mean value (right of dashed zero line) indicates that the abundance was higher in *Afte*r period than in *Before* period (no intervention). Diamond: overall mean effect size of random-effects model.
