# Supplementary material for: How do changes in flow magnitude due to hydropower operations affect fish abundance and biomass in temperate regions? A systematic review
Source: Environ Evid. 2022 Feb 4;11:3. doi: 10.1186/s13750-021-00254-8 (PMC8813579; doi:10.1186/s13750-021-00254-8)
Supplement: Supplementary file 1 — Additional file 1. ROSES systematic review report. [file 13750_2021_254_MOESM1_ESM.pdf]

<u>Additional File 1</u>. ROSES systematic review checklist (version 1.0).

Haddaway NR, Macura B, Whaley P, and Pullin AS. 2017. ROSES for Systematic Review Reports. Version 1.0. DOI: 10.6084/m9.figshare.5897272.

Section/sub-section	Topic	Description	Further explanation	Checklist/meta-data	Author response	Comments
Title	Title	The title must indicate that it is a systematic review, and should indicate if it is an update/amendment: e.g. "...A systematic review update."	The title should normally be the same or very similar to the review question.	Meta-data	How do changes in flow magnitude due to hydropower operations affect fish abundance and biomass in temperate regions? A systematic review	
Type of review	Type of review	Select one of the following types of review: systematic review, systematic review update, systematic review amendment, systematic review from a systematic map	See CEE Guidance on amendments and updates [1]	Meta-data	systematic review from a systematic map	
Authors' contacts	Authors' contacts	The full names, institutional addresses and email addresses for all authors must be provided.		Checklist	Yes	
Abstract	Structured summary	The abstract of the manuscript must not exceed 500 words and must be structured into separate sections: Background, the context and purpose of the review, including the review question; Methods, how the review was performed and statistical tests used (specifically mention search strategy, inclusion criteria, critical appraisal, data extraction and synthesis); Results, the main findings, including results of search and assessment of evidence base; Conclusions, brief summary and potential implications for policy/management and research.		Checklist	Yes	
Background	Background	Describe the rationale for the review in the context of what is already known. Reviews must indicate why this study was necessary and what it aims to contribute to the field.	A theory of change and/or conceptual model should be presented that links the intervention or exposure to the outcome.	Checklist	Yes	

Stakeholder engagement	Stakeholder engagement	The actual role of stakeholders throughout the review process (e.g. in the formulation of the question) must be described and explained (using a broad definition of 'stakeholder', including e.g. researchers, funders and other decision-makers; see [2])		Checklist	Yes	
Objective of the review	Objective	Describe the primary question and secondary questions (when applicable).	The primary question is the main question of the review. The secondary questions are usually linked to sources of heterogeneity (effect modifiers).	Checklist	Yes	
	Definition of the question components	Provide reference to the question key elements, e.g. population(s), intervention(s)/exposure(s), comparator(s), and outcome(s).	For other question types see [3,4]	Meta-data	Population: fish (resident and migrator) in temperate regions; Intervention: alterations to flow magnitude due to hydropower production and operation; Comparator: No intevention or alternative intervention; Oucomes: Abundance and biomass	
Methods	Protocol	Provide citation, DOI or open-access link to published protocol.	The protocol should be peer-reviewed and publicly available online (open access).	Meta-data	https://environmentalevidencejournal.biomedcentral.com/articles/10.1186/s13750-020-00198-5	
	Deviations from protocol	Describe any ways in which the final methods of the review deviate from those set out in the protocol along with a justification.		Checklist	Yes	

Searches	Search strategy	Detail the search strategy used, including: database names accessed, dates of searching, institutional subscriptions (or date ranges subscribed for each database), search options (e.g. ‘topic words’ or ‘full text’ search facility), efforts to source grey literature, other sources of evidence (e.g. hand searching, calls for evidence/submission of evidence by stakeholders).		Checklist	Yes	
	Search string	Provide Boolean-style full search string and state the platform for which the string is formatted (e.g. Web of Science format)		Meta-data	((Fish*) AND (“Fresh water” OR Freshwater OR Stream$ OR Water$ OR River$ OR Fluvial OR Estuar* OR Reservoir$ OR Impoundment$ OR "Hydro electric*" OR Hydroelectric* OR "Hydro dam*" OR Hydrodam* OR "Hydro power" OR Hydropower OR "Hydro" OR Dam$)) AND (Flow* OR Discharg*) AND (Productivity OR Biomass OR Abundance$ OR Densit* OR Yield$ OR “Ecological response” OR “Ecosystem response” OR “Biotic response”) NOT (Mining OR "Mine site" OR Aquaculture OR "Wastewater treatment" OR Carbon)	
	Languages - bibliographic databases	List languages used in bibliographic database searches		Meta-data	English	
	Languages – grey literature	List languages used in organisational website searches and web-based search engines		Meta-data	English	

	Bibliographic databases	Provide the number of bibliographic databases searched		Meta-data	8	Including two suggested by experts/practitioners
	Web-based search engines	Provide the number of web-based search engines searched		Meta-data	1	
	Organisational websites	Provide the number of organisational websites searched		Meta-data	1	
	Estimating comprehensiveness of the search	Describe the process by which the comprehensiveness of the search strategy was assessed (i.e. list of benchmark articles)		Checklist	Yes	
	Search update	Describe any update to searches undertaken during the conduct of the review	Compulsory (if update performed). A search update is good practice if original searches were performed more than two years prior to review completion.	Checklist	n/a	
Article screening and study inclusion criteria	Screening strategy	Describe the methodology for screening articles/studies for relevance. Methods for consistency of screening decisions (at title, abstract, and full texts levels) checking must be described.		Checklist	Yes	
	Inclusion criteria	Describe the inclusion criteria used to assess relevance of identified articles/studies. These must be broken down into the question key elements (e.g. relevant subject(s), intervention(s)/exposure(s), comparator(s), outcome(s), study design(s)) and any other restrictions (e.g. date ranges or languages).		Checklist	Yes	
Critical appraisal	Critical appraisal strategy	Describe here the method used for critical appraisal of study validity (including assessment of individual studies and the evidence base as a whole). Describe how repeatability of critical appraisal of study validity was tested.		Checklist	Yes	
	Critical appraisal used in synthesis	Describe how the information from critical appraisal was used in synthesis.		Checklist	Yes	

Data extraction	Meta-data extraction and coding strategy	Describe the method for meta-data extraction and coding for studies, providing lists of variables that will be extracted as meta-data and those that will be coded. Describe how repeatability of meta-data/data extraction and coding was tested.	Optional, a map database can be included within a systematic review	Checklist	Yes	
	Data extraction strategy	Describe the method for extraction of qualitative and/or quantitative study findings. Describe how repeatability of data extraction was tested.		Checklist	Yes	
	Approaches to missing data	Describe any process for obtaining and confirming missing or unclear information or data from authors.		Checklist	Yes	
Potential effect modifiers/reasons for heterogeneity	Potential effect modifiers/reasons for heterogeneity	Provide a list of and justification for the effect modifiers/reasons for heterogeneity that will be considered in the review. Also provide details of how the list was compiled (including consultation of external experts).		Checklist	Yes	
Data synthesis and presentation	Type of synthesis	State the type of synthesis conducted as part of the systematic review (narrative only, narrative and quantitative, narrative and qualitative, narrative, qualitative and quantitative, narrative and mixed-methods)		Meta-data	Narrative and Quantitative	
	Narrative synthesis strategy	Describe methods used for narratively synthesising the evidence base in the form of descriptive statistics, tables (including SM database) and figures. Study findings must only be narratively synthesised and vote-counting must be avoided.		Checklist	Yes	

	Quantitative synthesis strategy	If data are appropriate for quantitative synthesis, describe methods for calculating effect sizes, methods for handling complex data, statistical methods for combining data from individual studies, and any exploration of heterogeneity and publication bias. If all studies were not selected for synthesis explain criteria for selection (e.g. incomplete or missing information).	Compulsory (if quantitative synthesis performed)	Checklist	Yes	
	Qualitative synthesis strategy	Describe methods used for synthesising qualitative data and justify your methodological choices. Describe if and how you plan to analyse subgroups/subsets of data. If all studies were not selected for synthesis explain criteria for selection (e.g. incomplete or missing information).	Compulsory (if qualitative synthesis performed)	Checklist	N/A	
	Other synthesis strategies	Describe any other approaches used for synthesising data or combining qualitative and quantitative syntheses (e.g. mixed methods) and justify your choice of methodology.	Compulsory (if other synthesis performed)	Checklist	N/A	
	Assessment of risk of publication bias	Describe methods for examining the possible influence of publication bias on the synthesis.	This may be done for quantitative syntheses using diagnostic plots or statistical tests.	Checklist	Yes	
	Knowledge gap and cluster identification strategy	Describe the methods used to identify and/or prioritise key knowledge gaps (unrepresented or underrepresented subtopics that warrant further primary research) and knowledge clusters (well-represented subtopics that are amenable to full synthesis via systematic review).	Optional	Checklist	Yes	Identified knowledge gaps through descriptive statistics (geographical, taxonomic, seasonal)
	Demonstrating procedural independence	Describe the role of systematic reviewers (who have also authored articles to be considered within the review) in decisions regarding inclusion or critical appraisal of their own work.	Reviewers who have authored articles to be considered within the review should be prevented from unduly influencing inclusion decisions, for example by delegating tasks appropriately.	Checklist	Yes	

Results	Description of review process	Describe the review process including the volume of evidence identified from all sources and retained through each stage of the review. Must also display the number of articles/studies included at all stages of the review in a flow diagram, including the number of articles/studies excluded at each stage.		Checklist	Yes	
	Number of search results	Provide the number of search results from bibliographic databases (including updates if conducted) prior to duplicate removal.	This number should not include web-based search engine or organisational website searches: this will help assessment of the efficiency of the primary search string.	Meta-data	2966	This number does include results from Google Scholar, but does not include results from sources suggested by experts/practitioners (which account for an additional 1695)
	Number of search results after duplicate removal	Provide the total number of search results from bibliographic database searches following duplicate removal.	This number should not include web-based search engine or organisational website searches: this will help assessment of the efficiency of the primary search string.	Meta-data	3940	This number is the number removed after duplicates from database and records identified from other sources
	Full text screening excludes	Additional file containing list of and reasons for full text exclusions.		Checklist	Yes	
	Title screening results	Provide the number of articles retained following title screening.	Optional if screening titles and abstracts together	Meta-data	N/A	
	Abstract screening results	Provide the number of articles retained following abstract screening.	Optional if screening titles and abstracts together	Meta-data	N/A	
	Title and abstract screening results	Provide the number of articles retained following title and abstract screening.	Optional if screening titles and abstracts separately	Meta-data	1055	
	Retrieval results	Provide the number of articles retrieved at full text.		Meta-data	1038	plus an additional 95 from other sources, and 107 from the systematic map)
	Unobtainable articles	Additional file containing list of unobtainable articles.		Checklist	Yes	Included as a tab in Additional file 3

	Full text screening results	Provide the number of articles retained following full text screening.		Meta-data	211	
	Consistency checking: screening	Results of consistency checking at all stages (screening, data extraction, critical appraisal) must be provided. Provide the number of titles, abstracts and full texts screened and checked for consistency by two or more reviewers as a fraction of the total (e.g. Title: 2000/20000; Abstract: 500/5000: Full text: 10/100).		Checklist	Yes	
	Critical appraisal exclusions	If any studies are excluded due to low validity, provide the number of studies excluded from further synthesis during critical appraisal.	Compulsory for any studies not included in synthesis due to validity. Reviews authors may prefer to perform a sensitivity analysis (repeating analyses to examine the influence of validity) rather than excluding studies from synthesis.	Meta-data	N/A	
	Narrative synthesis	Describe the body of evidence identified using figures and tables, avoiding vote-counting (tallying of studies based on results; direction or significance). Each must be presented with descriptive information (meta-data) and extracted study findings. Describe the validity of individual studies and the evidence base as a whole.		Checklist	Yes	
	Extracted data	Additional file containing extracted quantitative or qualitative data (study findings) from included studies.		Checklist	Yes	
	Systematic map database	Additional file containing meta-data and coding for included studies.	Optional, a map database can be included within a systematic review	Checklist	Yes	
	Quantitative synthesis	Present results of quantitative synthesis of study findings (e.g. meta-analysis).	Compulsory (if quantitative synthesis performed)	Checklist	Yes	
	Qualitative synthesis	Present results of qualitative analysis of study findings (e.g. summaries of identified themes or categories). Also provide additional file with the identified themes or categories for each study.	Compulsory (if qualitative synthesis performed)	Checklist	n/a	

	Other synthesis	Present results of any other synthesis methods used.	Compulsory (if other synthesis performed)	Checklist	n/a	
	Risk of publication bias	Describe the results of assessments for the possible influence of publication bias on the synthesis.	For quantitative syntheses this may be done using diagnostic plots or statistical tests	Checklist	Yes	
Discussion	Discussion	Discuss the review results and suggest further enquiry or analysis (e.g. potential reasons for heterogeneity in outcome). Authors may draw attention to specific knowledge gaps.		Checklist	Yes	
	Limitations of the review	Discuss possible limitations in the methods used.		Checklist	Yes	
	Limitations of the evidence base	Discuss possible limitations in the evidence base.		Checklist	Yes	
Conclusions	Implications for policy/management	Summarise the state of the evidence base and discuss the way in which the identified evidence may inform policy/practice decision making in relation to the review question. Provide any measure of the uncertainty surrounding the outcome.	Reviews must not include practical environmental management recommendations or advocacy.	Checklist	Yes	
	Implications for research	Discuss the way in which the identified evidence may inform research including options for increasing the reliability of study design that could improve future research.	In this section some advocacy for future research on the reviewed topic is permissible provided it is clearly justified by the review outcome/critical appraisal of study validity.	Checklist	Yes	
Declarations	Competing interests	Describe of any financial or non-financial competing interests that the review authors may have.		Checklist	Yes	

References

[1] Bayliss, H.R., Haddaway, N.R., Eales, J., Frampton, G.K. and James, K.L., 2016. Updating and amending systematic reviews and systematic maps in environmental management. Environmental Evidence,
[2] Haddaway, N.R., Kohl, C., da Silva, N.R., Schiemann, J., Spök, A., Stewart, R., Sweet, J.B. and Wilhelm, R., 2017. A framework for stakeholder engagement during systematic reviews and maps in
[3] Collaboration for Environmental Evidence. 2018. Guidelines and Standards for Evidence synthesis in Environmental Management. Version 5.0. www.environmentalevidence.org/information-for-
[4] Leeds Institute of Health Sciences. https://medhealth.leeds.ac.uk/info/639/information_specialists/1500/search_concept_tools. Accessed 12/11/2017.
